# Probing Electrode–Electrolyte Synergy and Bottleneck Breakthrough of Zinc-Ion Capacitors from Two Key Configurations

**DOI:** 10.1007/s40820-025-02063-x

**Published:** 2026-02-17

**Authors:** Yudan Zhang, Hengyuan Hu, Yufeng Yan, Yuankai Huang, Yongbiao Mu, Zhiyu Zou, Kunxiong Zheng, Zhaoyang Yi, Yang Zhao, Miao Zhang, Lin Zeng, Meisheng Han

**Affiliations:** 1 School of Chemistry, Xi’an Jiaotong University, Xi’an, 710049 People’s Republic of China; 2 Shenzhen Key Laboratory of Advanced Energy Storage, Department of Mechanical and Energy Engineering, Southern University of Science and Technology, Shenzhen, 518055 People’s Republic of China; 3 College of Water Conservancy and Hydropower Engineering, Sichuan Agricultural University, Ya’an, 625014 People’s Republic of China

**Keywords:** Zinc-ion capacitors, Electrolyte, Pseudocapacitance electrodes, Battery-type electrodes

## Abstract

In response to the demanding requirements of next-generation energy storage systems for high-energy density, high-power density, and ultra-long-cycle life, the academic community has continued to focus on coupled devices that combine battery-level energy and capacitor-level power characteristics. Zinc-ion capacitors (ZICs) have become the most promising strategic candidate system for energy storage technology due to their high-energy/power characteristics, excellent intrinsic safety, and significant cost advantages. In this review, the latest research progress of ZICs is reviewed from the perspective of system. Firstly, ZICs are divided into zinc metal anode//capacitive cathode ZICs (ZC-ZICs) and capacitive anode//battery-type cathode ZICs (CB-ZICs) according to the device configuration, and the energy storage mechanisms are analyzed in depth. At the same time, focusing on the two configurations of ZC-ZICs and CB-ZICs and their electrolyte systems, problem-oriented the key puzzles and corresponding solutions are sorted out one by one. Finally, based on the above discussion, this review proposes forward-looking suggestions for material modifications of ZICs, including pulse voltage activation, application of high-entropy materials, and the development of stable and multi-functional electrolytes, aiming to provide scientific guidance for the practical application of high-performance ZICs and promote the in-depth development of high-performance ZICs research.

## Highlights

- This review provides a comprehensive discussion of the energy storage mechanisms, electrode materials, and electrolyte-related challenges in two key configurations: zinc metal anode//capacitive cathode zinc-ion capacitors (ZC-ZICs) and capacitive anode//battery-type cathode ZICs (CB-ZICs).
- This review provides comprehensive and effective solutions to the core bottleneck issues in the two key configurations.
- This review proposes forward-looking development roadmap of the ZICs, including pulse voltage activation on carbon electrodes, application of high-entropy materials as electrodes, and the development of stable and multifunctional electrolytes.

## Graphical abstract

**Figure:**
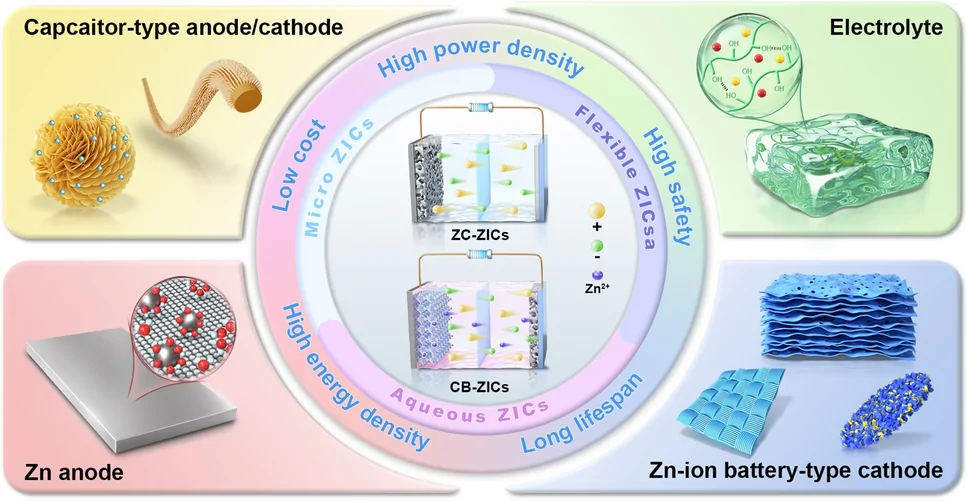

## Introduction

The excessive consumption of fossil energy (coal, oil, natural gas) by human society is accelerating the depletion of global energy. According to the existing mining intensity, fossil energy reserves are projected to face depletion risks within the coming decades. More critically, the huge amount of greenhouse gases (such as CO_2_, CH_4_) and pollutants (such as SO_X_, NO_X_, PM) released by its combustion lead to catastrophic crises such as global warming, extreme weather, acid rain, haze, and ecological degradation. This “lose-lose” situation of resource depletion and environmental degradation has forced the world to upgrade energy transformation to a national strategic level. Therefore, the large-scale development of green renewable energy has become a global consensus. Wind energy, tidal energy, hydropower, and solar energy have become an important part of the global energy system due to their advantages of inexhaustible and low carbon emissions. Globally, large-scale wind farms, photovoltaic power stations, hydropower stations, and tidal energy installations have been rapidly constructed, and installed capacity has continued to rise, reshaping the energy supply pattern.

However, the inherent “intermittency” and “fluctuation” of renewable energy are core challenges to its large-scale, efficient grid integration. Wind energy depends on wind speed, and output drops sharply when there is no wind. Photovoltaic energy is affected by day and night, seasons, and weather, and cannot generate electricity at night or in cloudy or rainy weather. Tidal energy has regular ebb and flow intervals. Hydropower is affected by seasonal precipitation. This unstable output, which is caused by natural factors and is difficult to control, is in sharp contrast to the core requirement of continuous, stable, and reliable power demand, posing a serious challenge to the stable, efficient, and safe operation of the power grid. Fortunately, the combination of energy storage devices with large-capacity energy storage characteristic and fast charging and discharging with renewable energy systems can effectively solve this problem [1–3]. At the same time, the explosive popularity of small portable electronic devices (smart phones, laptops, wearable devices) and the rapid rise of the new energy electric vehicle industry have promoted the energy storage system with excellent electrochemical characteristics to become the “energy heart” of modern society [4]. Currently, batteries are widely utilized globally as an optimal option for integrating renewable energy, primarily owing to their high-energy density, mature technology, and high-energy conversion efficiency. However, batteries still suffer from notable drawbacks, as their redox reactions are often accompanied by an unstable electrode–electrolyte interface structure (causing side reactions and solid electrolyte interphase (SEI) growth), concentration polarization and electrochemical polarization during charging and discharging, and slow reaction kinetics [5–7]. These factors lead to a short cycle life, and it is difficult to achieve real high-rate charge and discharge. In contrast, supercapacitors exhibit distinct characteristics: their energy storage relies on the physical adsorption of the double-layer at the electrode/interface (EDLC) or rapid reversible Faradaic reactions at the surface (pseudocapacitance). This energy storage mechanism endows them with extremely high-power density, ultra-long-cycle life, and fast charging/discharging capabilities. This makes supercapacitors indispensable in scenarios requiring instantaneous high-power bursts (such as electric vehicle startup/braking energy recovery) or frequent charging/discharging cycles. Nevertheless, their inherently low-energy density prevents supercapacitors from meeting long-duration energy storage requirements [8].

To bridge the “performance gap” between batteries and supercapacitors and achieve synergistic optimization of energy, power, and lifespan characteristics, hybrid supercapacitors (HSCs) have seen rapid development. These devices combine battery-type electrodes with the Faradaic process and capacitor-type electrodes based on physical energy storage mechanisms, forming an asymmetric structure [9, 10]. HSCs achieve complementary advantages and synergistic enhancements of the two mechanisms through ingenious design: combining the high-energy storage capacity of batteries with the fast response/long lifespan of supercapacitors. This provides a more balanced and competitive solution for scenarios requiring both high-energy reserves and fast charging/discharging/long lifespan, demonstrating significant application potential [11, 12].

HSCs can be classified into various systems based on the charge carrier types, primarily including lithium-ion (Li^+^), sodium-ion (Na^+^), potassium-ion (K^+^), and zinc-ion (Zn^2+^) HSCs. The core difference between these systems lies in the active cations in the electrolyte and their corresponding electrochemical behaviors. Compared with other metal ion systems, zinc-ion hybrid supercapacitors (ZICs) demonstrate unique advantages in many aspects due to the unique physicochemical properties of zinc metal and zinc ions: (1) Zinc has a relatively low redox potential (− 0.76 V vs. standard hydrogen electrode, SHE). Its two-electron transfer reaction (Zn^2+^/Zn) can store more charge than the one-electron transfer of monovalent ions (Li^+^, Na^+^, K^+^) at the same mass or volume, significantly improving the theoretical energy density of the device (up to 820 mAh g^−1^, 5855 mAh cm^−3^). (2) Rapid reaction kinetics and excellent cycle stability, zinc ions usually exhibit rapid deintercalation/intercalation or deposition/dissolution reaction kinetics in aqueous electrolytes. At the same time, the zinc anode can exhibit excellent electrochemical reversibility and long-cycle stability in the appropriate electrolyte system. (3) Zinc has a significantly higher crustal abundance than elements such as lithium, cobalt, and nickel, with abundant reserves, widespread distribution, and low-cost (costs far below those of lithium), significantly reducing raw material costs and supply chain risks. Its mining, processing, and recycling processes are also relatively mature and environmentally friendly. (4) Zinc metal reacts much less violently in aqueous electrolytes than highly reactive alkali metals, making it less prone to violent thermal runaway or combustion and explosion, thereby conferring greater safety to ZICs [13–16]. The synergy of these advantages promotes ZICs to become a promising sustainable energy storage system.

The core structure of ZICs is composed of battery electrode, capacitive electrode, and electrolyte. The battery-type electrode mainly uses zinc metal foil, and the energy storage is based on the reversible electrodeposition/dissolution of Zn^2+^ on the electrode surface (Zn ↔ Zn^2+^ + 2e^−^). Materials based on Zn^2+^ insertion/conversion reactions can also be used. Its core role is to provide a high specific capacity and stable discharge platform, contributing to the main energy storage [17]. The capacitive electrode uses a high specific surface area (SSA) and high-conductivity material, and the energy storage is based on EDLC and surface/near-surface fast reversible Faradaic reactions (pseudocapacitance). Commonly employed materials consist of graphene, carbon nanotubes, activated carbon (AC), as well as transition-metal compounds (TMCs). Its advantage lies in rapid charge storage kinetics, yielding exceptional power density coupled with remarkable cycle longevity. Electrolytes serve as the “bridge” connecting the two electrodes and the “highway” for ion transport, making their function critically important. Electrolytes achieve rapid charge balance by efficiently transporting Zn^2+^, thereby synergistically optimizing the potential difference between the two electrodes. Additionally, the wide electrochemical window of electrolytes can accommodate the high-voltage Faradaic reactions and stabilize the EDL of capacitive electrodes. Common electrolyte systems include aqueous electrolytes, non-aqueous electrolytes, and solid/quasi-solid electrolytes.

Although ZICs have great development prospects and are ideal candidates for battery alternatives, they still face key challenges. The uneven deposition of zinc anode leads to the generation of dendrites, which may penetrate the separator and cause short circuits, cause safety hazards, and shorten the service life. Zinc is thermodynamically unstable in the aqueous system, self-corrosion, or hydrogen evolution reaction (HER) occurs, consuming active material, resulting in a decrease in efficiency [18]. The corrosion products of zinc anode (such as ZnO, Zn(OH)_2_) form a low conductive layer, which increases the interface impedance and accelerates the capacity decay. In addition, traditional carbon-based cathode materials have relatively low specific capacity (often < 200 F g^−1^). Although pseudocapacitive materials (such as MnO_2_, V_2_O_5_) have high capacity, they face the problems of poor intrinsic conductivity and unstable structure. The large ionic radius and high charge density of Zn^2+^ lead to its slow diffusion in the inserted cathode material, which limits the rate performance. Moreover, some materials (such as vanadium-based and manganese-based) are easy to dissolve or undergo irreversible phase transition during the cycle, resulting in continuous capacity decay. For electrolytes, the operating voltage window of traditional aqueous electrolytes is relatively narrow (< 2.0 V). Although it can be broadened by high-concentration electrolytes, deep eutectic solvents, etc., it is often accompanied by problems such as increased costs and increased viscosity. At present, many researchers have focused on the core strategies to deal with the challenges of ZICs, but not limited to inhibiting dendrite/corrosion through negative interface modification and three-dimensional structure design, developing high-capacity cathode materials and optimizing their structure/interface stability, using high-concentration electrolytes and new electrolyte systems to broaden the voltage window and inhibit side reactions.

This review provides a systematic and comprehensive overview of the key challenges and latest developments in ZICs. First, ZICs are categorized into two main configurations ZC-ZICs and CB-ZICs based on their energy storage mechanisms. The energy storage mechanisms and typical electrode material systems of each configuration are analyzed in depth, and the core challenges faced by each configuration are summarized, along with corresponding strategies for addressing them. Subsequently, the article focuses on the deep-seated electrode and electrolyte scientific challenges in the development of ZICs, specifically within ZC-ZICs and CB-ZICs. It thoroughly elucidates the bottlenecks, systematically reviews the various innovative solutions proposed globally to address these challenges, and objectively analyzes their mechanisms of action, implementation effects, and advantages and disadvantages. It also systematically reviews solutions proposed to date. Finally, based on the latest international trends and research developments, this paper provides a forward-looking outlook on the future development of ZICs. It is hoped that this review will provide detailed references for research on next-generation zinc-ion energy storage systems, promote the deep integration and large-scale application of high-performance, low-cost, high-safety, and environmentally friendly ZICs technology in energy systems, and contribute to addressing energy and environmental challenges. It is also hoped that this review will inject vigorous momentum into research on next-generation zinc-ion energy storage systems, driving their deep integration and large-scale application in modern society.

## Compositions of ZICs

As an integrated device combining zinc-ion batteries and supercapacitors, ZICs naturally combine the advantages of both. Compared to zinc-ion batteries, ZICs not only enable fast charging and discharging but also exhibit excellent cycle stability. Compared to supercapacitors, ZICs provide high-energy storage capabilities alongside excellent power delivery, achieving high-power output without sacrificing capacity. Additionally, the low-cost and high safety of ZICs further enhance their development potential [19–21]. For example, ZICs are often used as flexible solid-state devices and microdevices in wearable and miniaturized electronic devices [4, 22–25]. They can also be connected to other energy harvesting systems. Therefore, to facilitate the large-scale implementation of ZICs, comprehensive investigation into the correlation between their key components (including battery-type electrodes, capacitor-type electrodes, electrolytes, and separators) and overall device performance remains essential.

### Electrodes

As the core component of ZICs, the electrode material directly determines its key performance such as energy density, power density, and cycle life. According to the different energy storage mechanisms, the electrode materials of ZICs can be divided into battery-type electrodes and capacitor-type electrodes [26–28]. The battery-type electrode enables energy storage via the deposition/dissolution or insertion/extraction process of zinc ions, providing high theoretical capacity and a wide-voltage window for ZICs [29]. The corresponding electrode materials include zinc metal, vanadium oxides, and manganese oxides. In addition, capacitor-type electrodes store charge through the adsorption/desorption process of zinc ions on the surface, providing high-rate capability and exceptional longevity for ZICs. The representative electrode materials are carbon materials and pseudocapacitive materials. Based on the use of electrodes in the cathode or anode, ZICs can be divided into two configurations. The first is a zinc-ion capacitor (ZC-ZIC) composed of a zinc metal anode and a capacitor-type cathode. The second is a zinc-ion capacitor (CB-ZIC) that couples a capacitor-type electrode (as the anode) with a battery-type electrode (as the cathode) [28].

#### ZC-ZICs

**Fig. 1:**
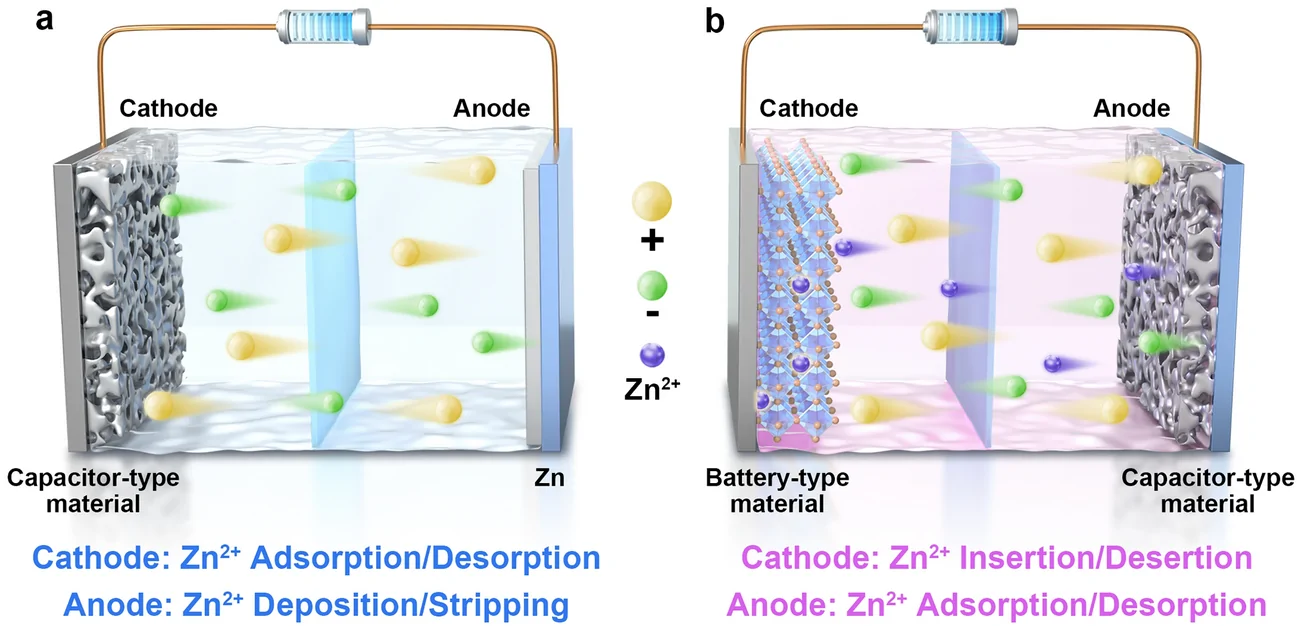
Schematic diagram of the operating principle of ZICs

The zinc metal anode//capacitive cathode zinc-ion capacitors (ZC-ZICs) are constructed by a metal zinc anode and a capacitive (mainly carbon materials) cathode, and the energy storage mechanism is shown in Fig. 1a. During charging, anions in the electrolyte migrate to the cathode and are adsorbed, while zinc ions in the electrolyte migrate and deposit on the surface of the metallic zinc anode [21, 30]. During discharging, both anions and zinc ions leave the electrodes and migrate back into the electrolyte [31, 32]. Importantly, the deposition and stripping of zinc on the foil electrode can be summarized as a reversible redox reaction between the zinc foil and the electrolyte, i.e., the Faradaic process, expressed by Eq. (1) [30, 33]:

1 $${\mathrm{Zn}} \leftrightarrow {\mathrm{Zn}}^{{2 + }} + 2{\mathrm{e}}^{ - }$$

**Fig. 2:**
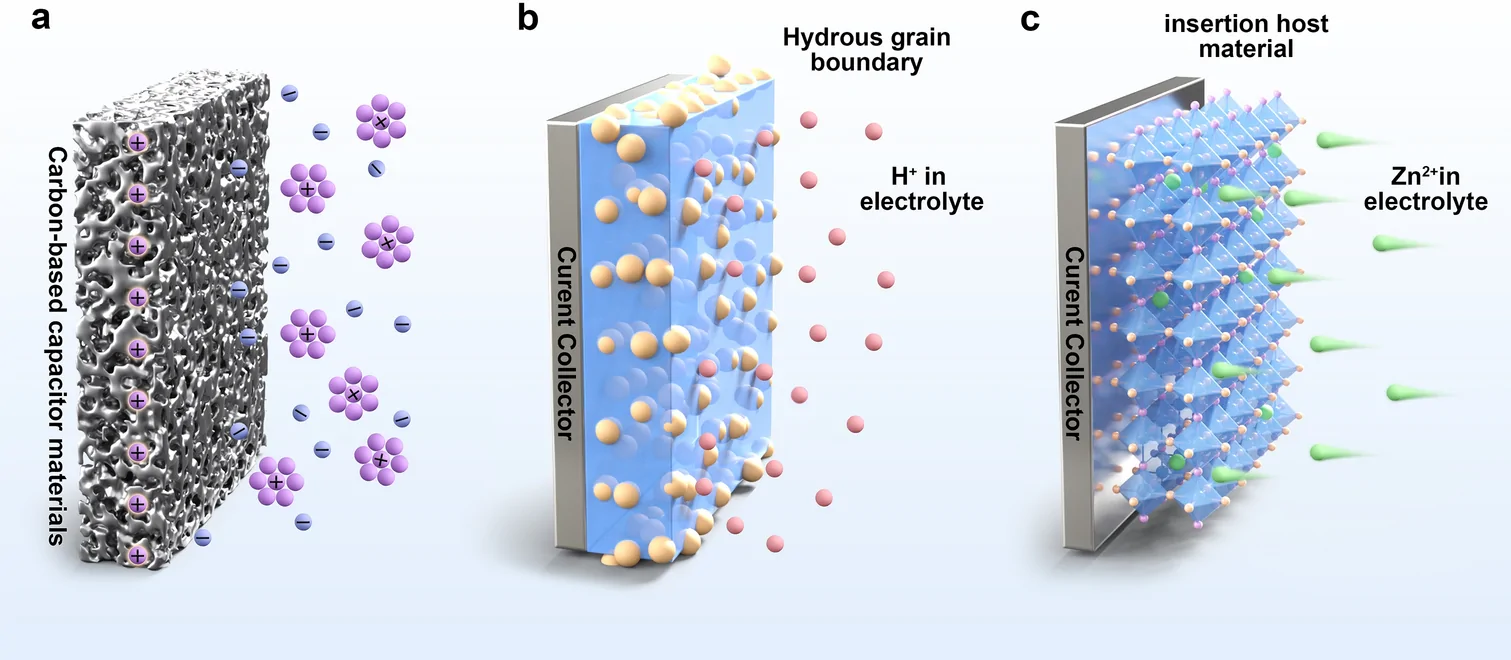
**a** EDLC energy storage mechanism. Pseudocapacitance energy storage mechanism** b** oxidative-reducing type; **c** intercalated type

Due to the adsorption/desorption process of anions on the cathode surface, there is an accumulation of electrostatic energy between the cathode surface and the electrolyte interface, establishing an EDLC (Fig. 2a) [34]. The interaction equation between the anion X^−^ and the carbon electrode material is as follows [29]:

2 $$C + X^{ - } \leftrightarrow C//X^{ - }$$

It is worth noting that the Helmholtz double-layer capacitance storage mechanism of carbon electrodes is closely related to the SSA of carbon materials [28]. Limited SSA makes it difficult for carbon electrodes to match the excellent performance of zinc anodes in terms of reversible capacity during charging and discharging. In-depth analysis shows that the fundamental reason is that the adsorption/desorption rate of ions in the capacitive cathode is significantly lower than the deposition/stripping rate of zinc ions in the zinc anode, which results in the kinetic imbalance between the two and leads to the limited electrochemical performance of ZC-ZICs [35–37]. Fortunately, the defect of double-layer capacitors can currently be remedied by introducing pseudocapacitive storage mechanisms. For example, strategies such as doping modification and surface functional group adjustment can be used on carbon materials, or more suitable pseudocapacitive materials can be explored, so that capacitive materials can simultaneously possess double-layer capacitance and pseudocapacitive storage mechanisms to store more charge [38]. It is important to note that pseudocapacitive effects can be divided into surface redox reactions and ion insertion/extraction reactions. The main difference is that the surface redox reaction is the redox reaction of the bond of the compound on the electrode surface, while the ion insertion/extraction reaction is the ion insertion in the electrode material (Fig. 2b, c) [3, 39].

Although the synergistic mechanism between the electric double-layer, pseudocapacitive effect, and battery-type zinc anode Faradaic process in carbon electrode materials can to some extent enhance the power density and energy density of ZC-ZICs, there are still many challenges in the development of ZC-ZICs [31, 40–42]. In particular, zinc anodes exhibit limited cycle life due to dendrite growth, HER and corrosion reactions. The main mechanism is that the non-atomically smooth zinc foil leads to uneven deposition of zinc ions at higher current densities, which eventually promotes the accumulation of zinc dendrites [32, 43, 44]. The growth of zinc dendrites can indirectly accelerate the HER rate and consume the protons in the aqueous electrolyte. The equation is expressed as [45]:

3 $$2{\mathrm{H}}_{2} {\mathrm{O}}\left( {\mathrm{l}} \right) + 2{\mathrm{e}}^{ - } \to H_{2} \left( {\mathrm{g}} \right) + 2{\mathrm{OH}}^{ - } ({\mathrm{aq}})$$

Subsequently, the hydroxide accumulated on the surface of the zinc anode reacts with the zinc anode to cause corrosion reaction. The consumption of the zinc anode greatly reduces the utilization efficiency and adversely affects the cycling stability of the device. In addition, the main by-product ZnO generated by the corrosion reaction will reduce the conductivity of ZC-ZICs, making the electric field more disordered and promoting the further growth of zinc dendrites [46–48]. The equation of the formation of the main by-product ZnO in the local alkaline environment is expressed as [45]:

4 $${\mathrm{Zn}}^{{2 + }} \left( {{\mathrm{aq}}} \right) + 2{\mathrm{OH}}^{ - } \left( {{\mathrm{aq}}} \right) \to {\mathrm{Zn}}\left( {{\mathrm{OH}}} \right)_{2} \left( {\mathrm{s}} \right) \to {\mathrm{ZnO}}\left( {\mathrm{s}} \right) + {\mathrm{H}}_{2} {\mathrm{O}}({\mathrm{l}})$$

In practical applications, zinc dendrites may pierce the separator, causing short circuits and posing safety hazards [28, 49]. In addition, the interaction between water molecules and water-soluble zinc salts can significantly affect ion adsorption/desorption or deposition/stripping, resulting in a deterioration of the device’s electrochemical performance. It should be noted that most ZC-ZICs currently under study typically consist of zinc foil weighing tens of milligrams to several hundred milligrams matched with several milligrams of AC material. In contrast to kinetic equilibrium, the mass of zinc foil with high theoretical specific capacity (820 mAh g^−1^) is much higher than that of AC materials with low reversible capacity. The reason lies in the fact that zinc anodes often fail to utilize zinc ions efficiently during charging and discharging, necessitating the use of more zinc foil to supply zinc ions. The low utilization rate of zinc foil is also a key factor limiting the large-scale application of ZC-ZICs. Therefore, addressing these challenging issues is indispensable for the practical application of ZC-ZICs in the future [28, 32, 50, 51].

#### CB-ZICs

Capacitive anode//battery-type cathode zinc-ion capacitors (CB-ZICs) are mainly composed of battery-type cathodes and capacitor-type anodes. The electrode materials of battery-type cathodes are mainly manganese-based oxides or vanadium-based oxides, while capacitor-type anodes still use carbon or pseudocapacitive materials. The corresponding energy storage mechanisms are shown in Fig. 1b. Specifically, during the first charge (activation) process, the Zn^2+^ in the electrolyte is inserted in the cathode lattice to provide it with the initial Zn^2+^ (or pre-embedded in battery-type material). During subsequent charging, zinc ions dissociated from the cathode migrate through the electrolyte to be electrodeposited onto or incorporated into the anode, while anions migrate from the electrolyte to the cathode [52, 53]. During discharging, zinc ions desorb or deintercalate from the surface of the anode, traverse the electrolyte, and are intercalated into the cathode, while anions are released from the cathode surface and diffuse into the electrolyte. As a battery-type cathode, the charge storage mechanisms of manganese-based oxides or vanadium-based oxides are different from that of zinc anodes. The charge storage of manganese-based oxides or vanadium-based oxides mainly depends on the insertion/extraction reaction of zinc ions and the reversible redox reaction of metal oxides, while zinc ions are inserted and deintercalated in the tunnel structure or layered structure of manganese-based and vanadium-based oxides. It should be noted that the oxidation reaction occurs with the electrode material during the insertion process, and the reduction reaction occurs with the electrode material during the deinsertion process [28].

The manganese-based/vanadium-based oxides replace the metal zinc as the battery-type electrode, which optimizes the device design of ZICs, avoids the zinc dendrite phenomenon, and makes the device exhibit better cycle performance. Unfortunately, CB-ZICs still face challenges in their development. For example, low-cost, diverse, and highly safe manganese-based oxides have structural instability, which causes device capacity degradation, and trivalent manganese ions are prone to disproportionation reactions in non-alkaline environments [54, 55]. Vanadium-based oxides, which have low redox potential and are abundant in resources, also face the problem of layer structure deformation and dissolution after multiple charge–discharge cycles. In addition, vanadium-based oxides have potential toxicity and slow zinc-ion diffusion kinetics [56].

### Electrolytes

Electrolytes are an important component of ZICs. They not only provide an ionic transport medium for electrode reactions, but also directly govern the establishment of the electric double-layer at the electrode–electrolyte boundary. More importantly, they determine the operating voltage window of ZICs, which can affect the energy-power characteristics of ZICs. In general, electrolytes can be divided into the following three categories: (1) aqueous electrolytes, (2) non-aqueous electrolytes (organic electrolytes, ionic liquid electrolytes, polymer electrolytes), (3) solid/quasi-solid electrolytes (hydrogel electrolytes, polymer solid electrolytes). Although aqueous electrolytes have significant advantages such as high-ionic conductivity, low-cost, and environmental friendliness, they are limited by the formation of Zn dendrites and parasitic reactions such as HER, resulting in a narrow voltage window that restricts the improvement of ZICs cycle life. Meanwhile, the mismatch between the radius of the solvated zinc composite ions in the electrolyte and the pore structure of the material also represents a key constraint on the achievable performance of ZICs. The main reason is that when the radius of the solvation zinc composite ion is greater than the pore size of the carbon electrode, the solvation ions cannot enter the micropores, resulting in a large number of effective surface areas that cannot be utilized, and the kinetics is slow. After the pore size distribution is matched with the ion size, the electrode SSA can be maximized to avoid the “dead hole” phenomenon and improve the utilization rate of effective adsorption sites. Therefore, suppressing side reactions, expanding the working voltage window, and lowering zinc ions’ solvation degree to fit material structure are key to electrolytes’ future development.

Furthermore, research has found that the pH of aqueous electrolytes plays a critical role in determining the performance of ZICs [35, 57]. Alkaline electrolytes accelerate corrosion reactions and are incompatible with cathode materials, resulting in disappointing performance of ZICs, while acidic electrolytes accelerate side reactions such as HER [58]. Therefore, neutral and weakly acidic aqueous electrolytes are often used in practical applications. In addition, some electrolyte additives can promote preferential interactions between the electrode material and zinc ions, effectively crowding out H^+^ and thereby limiting the side reaction of HER and increasing the voltage window. Moreover, adjusting the type of zinc salt and the electrolyte concentration are considered to reduce the radius of the solvated zinc complex ions and improve the performance of ZICs. The corresponding mechanism is that the mixed electrolytes with different zinc salt concentrations can alter the solvation structure of solvated zinc ions. For example, the common solvated ion in zinc sulfate is [Zn(H_2_O)_6_]^2+^ (with a radius of 8.60 Å). After the addition of ZnCl_2_, the solvated ion will be transformed into [ZnCl]^+^(H_2_O)_n−1_ (with n = 1–6) with a lower ionic radius, which contributes to the adsorption/desorption of ions. In addition, the use of non-aqueous electrolytes is also an important strategy to improve the voltage window. Among them, organic electrolytes can achieve high-voltage windows, but their low ignition points, toxicity, and volatility pose major challenges for practical applications. Secondly, ionic liquid electrolyte is a conductive system constructed by molten salt at room temperature. They not only have good chemical stability, but also have advantages of high thermal stability, wide-voltage window, and high safety. It has become an ideal choice for high-reliability energy storage systems under extreme conditions. Unfortunately, ionic liquids are expensive and industrial applications are limited [59]. In addition, polymer electrolytes are often used in ZICs as they prevent liquid electrolyte leakage in flexible ZICs and have exceptional ionic transport and robust mechanical strength. However, organic electrolytes’ toxicity and flammability limit their large-scale use [3].

For the application requirements of ZICs, solid/quasi-solid electrolyte systems show unique potential. For example, hydrogel electrolytes are considered to be an ideal choice for flexible and wearable ZICs devices due to their good flexibility and moderate ionic conductivity, but their problems of easy freezing at low temperature and decreased ionic conductivity still need to be overcome. As an important subclass of quasi-solid electrolytes, hydrogel electrolytes exhibit the solid-like mechanical stability of quasi-solid systems. However, due to their relatively high water content, while they provide higher flexibility and faster ion transport, they compromise low-temperature tolerance and long-term structural stability [60]. Although polymer solid electrolytes have high mechanical strength and good environmental stability, they can effectively inhibit zinc dendrites and avoid electrolyte leakage, improving device safety. However, their room temperature ionic conductivity is usually low, and the interface impedance is large, which limits the rate performance of ZICs. Therefore, the development of solid electrolytes with high-ionic conductivity, good interfacial compatibility, and wide electrochemical window in ZICs is still an important direction to improve their comprehensive performance.

### Separators

The separator is a key component in ZICs. The presence of a separator between the two electrodes mitigates the risk of internal short circuits by preventing direct physical contact, while allowing the free transport of zinc ions in the electrolyte. However, the insertion of the separator occupies the space inside ZICs and increases the internal resistance of the ZICs. Therefore, the ideal separator should have the characteristics of low thickness, high porosity, low resistivity, and good chemical stability. Depending on the material type, membranes can be classified into glass fiber membranes, polymer membranes (such as polypropylene and polyethylene), cellulose-based membranes, and composite membranes. Each type of membrane has its own characteristics. Among them, composite membranes, which are made by combining multiple materials, have high porosity, high mechanical strength, and chemical stability, and have attracted widespread attention from researchers.

## Bottlenecks and Modification Strategies of ZC-ZICs

The ZC-ZICs work together through the redox reaction of the zinc anode and the ion adsorption/desorption mechanism of the capacitive cathode to achieve the balance of energy density and power density. The capacitive cathode relies on the ion adsorption/desorption mechanism of porous carbon-based materials to achieve high-power output. In addition, pseudocapacitive materials have gradually become a research hotspot. This kind of material realizes charge storage through fast reversible redox reactions on or near the surface, which has the advantages of high specific capacity and fast reaction kinetics. Zinc anode provides energy advantages for the system with high theoretical capacity and low redox potential, but its dendrite growth and side reaction problems still need to be suppressed by means of surface engineering and electrolyte optimization. In this review, the problems faced by carbon materials and pseudocapacitive materials are classified and the corresponding solutions are proposed.

### Capacitive Cathode

In ZC-ZICs, capacitor-type electrodes as core components can be divided into two categories: carbon-based materials and pseudocapacitive materials. Compared with the battery-type electrodes based on redox reactions, the capacitive electrode achieves energy storage through reversible surface adsorption/desorption (carbon material) or intercalation/deintercalation (pseudocapacitive material) of Zn^2+^, showing better rate performance and cycle stability. However, the low specific capacity of the capacitive electrodes directly leads to the low overall energy density of the device. Therefore, the development of new capacitor-type electrodes with high capacity and fast dynamic characteristics has become a key direction to break through the energy density bottleneck of ZICs. In this review, the carbon-based and pseudocapacitive materials are classified according to the problems, and the corresponding solutions are proposed, and the corresponding performance comparison tables are summarized as shown in Tables 1 and 2.

**Table 1:** Comparative summary of the challenges, regulation strategies, and electrochemical performance of porous carbon materials for ZICs

Problem	Strategy	Cathode	Anode	SSA	*P*_v_	Electrolyte	Capacity	*E*_d_	*P*_d_	*C*_R_ (*C*_n_; *C*_d_)	References
Lack of specific surface area	Ice-templating-assisted activation	PCC-3	Zn foil	1508 m^2^ g^−1^	NA	Zn(CF_3_SO_3_)_2_	344 F g^−1^ at 0.5A g^−1^	119.02 Wh kg^−1^	13.2 kW kg^−1^	99% (10,000; 5 A g^−1^)	[64]
	Metal salt template	ENHPC-0.5	Zn foil	3122 ± 25 m^2^ g^−1^	1.57 ± 0.02 cm^3^ g^−1^	ZnSO_4_	350 F g^−1^ at 0.1 A g^−1^	NA	NA	NA	[65]
	Hydrogen bond micelle templating	TB-DA-80	Zn foil	2370 m^2^ g^−1^	2.18 cm^3^ g^−1^	Zn(CF_3_SO_3_)_2_	250 mAh g^−1^ at 0.2 A g^−1^	163 Wh kg^−1^	12.1 kW kg^−1^	90.5% (400,000; 20 A g^−1^)	[66]
	Hydrogen bond micelle self-assembly	CNS-2	Zn foil	2416 m^2^ g^−1^	1.4 cm^3^ g^−1^	Zn(CF_3_SO_3_)_2_	263 mAh g^−1^ at 0.2 A g^−1^	163 Wh kg^−1^	12.7 kW kg^−1^	93% (200,000; 20 A g^−1^)	[67]
	Self-assembly	rGO/75%FRGO	Zn foil	72.84 m^2^ g^−1^	0.82 cm^3^ g^−1^	ZnSO_4_	NA	113.1 Wh L^−1^	21.2 kW L^−1^	96.4% (8,000; 0.5 A g^−1^)	[68]
	Biomass template	CB-3–850	Zn foil	1967.3 m^2^ g^−1^	0.86 cm^3^ g^−1^	ZnSO_4_	239.1 mAh g^−1^ at 0.5A g^−1^	213.4 Wh kg^−1^	14.8 kW kg^−1^	90% (10,000;20 A g^−1^)	[69]
	Doping modification	HC-0.2	Zn foil	1902.9 m^2^ g^−1^	NA	ZnSO_4_	245.8 mAh g^−1^ at 0.2 A g^−1^	164.1 Wh kg^−1^	30.1 kW kg^−1^	NA	[70]
Limited energy storage mechanisms	Nanoconfined carbonization	HDPC-1	Zn foil	591.0 m^2^ g^−1^	0.78 cm^3^ g^−1^	NA	453 F g^−1^ at 0.1A g^−1^	204.0 Wh kg^−1^	45.0 kW kg^−1^	NA	[75]
	Metal single-atom anchoring	MnSA/NCN	Zn foil	1309.0 m^2^ g^−1^	NA	Zn(CF_3_SO_3_)_2_	203.0 mAh g^−1^ at 0.1 A g^−1^	138.0 Wh kg^−1^	15.65 kW kg^−1^	91.0% (10,000; 5 A g^−1^)	[78]
	Doping modification	B/N@AC	Zn foil	2366.0 m^2^ g^−1^	1.42 cm^3^ g^−1^	ZnSO_4_	330.0 F g^−1^ at 0.2A g^−1^	126.4 Wh kg^−1^	17.04 kW kg^−1^	87.5% (7000; 5 A g^−1^)	[74]
		rGOSH	Zn foil	78.96 m^2^ g^−1^	NA	Zn(CF_3_SO_3_)_2_	540.0 F g^−1^ at 0.1A g^−1^	NA	NA	NA	[77]
		Zn-MET-800	Zn foil	525.6 m^2^ g^−1^	NA	ZnSO_4_	361.6 F g^−1^ at 0.2 A g^−1^	128.5 Wh kg^−1^	4.7 kW kg^−1^	90.3% (30,000; 10 A g^−1^)	[72]
		ZIF-8/cel	Zn foil	1947.0 m^2^ g^−1^	NA	ZnSO_4_	16.9 F cm^−2^ at 5 mA cm^−2^	7.23 mWh cm^−2^	34.67 mW cm^−2^	NA (2000; NA)	[73]
		LEST-FG-74	Zn foil	144.0 m^2^ g^−1^	NA	Zn(CH_3_COO)_2_	19.5 μ Ah cm^−2^ at 1 mA cm^−2^	10.93 μ Wh cm^−2^	92 μ W cm^−2^	65.0% (10,000; 2 mA cm^−2^)	[76]
	Composite material	AMCTS- CC@Mo_3_	CC@N-HPC	NA	NA	PAM-2M ZnSO_4_	2.3 F cm^−2^ at 1 mA cm^−2^	321.0 μ Wh cm^−2^	8.0 mW cm^−2^	90.0% (10,000 20 mA cm^−2^)	[79]
		P-MoO_2-X_@NP-C	N–C-A	NA	NA	Zn(CF_3_SO_3_)_2_ + LiCF_3_SO_3_	369.1 mAh g^−1^ at 0.1 A g^−1^	43.8 Wh kg^−1^	NA	88.2% (32,000; 2 A g^−1^)	[80]
		C/LIG/poly(8-amino-2-naphthol)	Zn foil	NA	NA	ZnSO_4_	308.0 mAh g^−1^ at 0.1 mA cm^−2^	1.03 mWh cm^−2^	NA	99.98% (10,000; 5 mA cm^−2^)	[81]
Slow Diffusion Kinetics	Biomimetic hollow carbon nanofibers	CNF–Zn-800	Zn foil	1201.0 m^2^ g^−1^	NA	ZnSO_4_	156.0 mAh g^−1^ at 0.2 A g^−1^	132.8 Wh kg^−1^	15.0 kW kg^−1^	98.7% (80,000; 10 A g^−1^)	[84]
	Hydrothermal coupling double salt activation	ODRMCs-M1/K1	Zn foil	2271.0 m^2^ g^−1^	NA	ZnSO_4_	169.4 mAh g^−1^ at 0.3 A g^−1^	135.5 Wh kg^−1^	24.0 kW kg^−1^	108.2% (150,000; 10 A g^−1^)	[85]
	Solvent-guided heterogeneous atomic carbon superstructures	CS_DMF_	Zn foil	1780 m^2^ g^−1^	NA	Zn(OTF)_2_	240.0 mAh g^−1^ at 0.5 A g^−1^	145.0 Wh kg^−1^	32.0 kW kg^−1^	90.9% (300,000; 50 A g^−1^)	[86]
	Hierarchically ordered porous carbon	PN-HOPC	Zn foil	1701.8 m^2^ g^−1^	NA	ZnSO_4_	211.9 mAh g^−1^ at 0.2 A g^−1^	169.5 Wh kg^−1^	64.0 kW kg^−1^	99.3% (60,000; 10 A g^−1^)	[87]
	N/S codoped porous hollow carbon nanofibers	S-HPCNFs	Zn foil	220.34 m^2^ g^−1^	NA	ZnSO_4_	221.5 mAh g^−1^ at 0.1 A g^−1^	74.7 Wh kg^−1^	0.184 kW kg^−1^	NA	[88]

SSA, specific surface area;* P*_v_, pore volume;* E*_d_, energy density; * P*_d_, power density; * C*_R_, capacity retention (%);* C*_n_, cycle number; * C*_d_, current density; NA, not available

**Table 2:** Comparison of organic metal-framework and transition-metal-based pseudocapacitive materials: challenges, strategies, and their electrochemical performance in ZICs

Classification	Problem	Strategy	Material	Anode	Capacity	SSA	* P*_v_	Electrolyte	*E*_d_	* P*_d_	* C*_R_ (* C*_n_; * C*_d_)	Refs
Organic metal framework	Poor intrinsic conductivity	Metal doping	Zn_1_Cu_X_MC	Zn NFAs	134 mAh g^−1^ at 0.5 A g^−1^	910 m^2^ g^−1^	NA	ZnSO_4_	100 Wh kg^−1^	0.38 kW kg^−1^	91% (40,000; 32 A g^−1^)	[89]
		Build a heterojunction	COF-5/Ti_3_C_2_T_X_	Zn foil	952 mF cm^−2^ at 0.1 mA cm^−2^	12.8 m^2^ g^−1^	NA	SPZHE	160 mWh cm^−2^	1709 mW cm^−2^	80.2% (2000; 1 mA cm^−2^)	[90]
	Structural collapse	Construction of core–shell structure	CM-P	Zn foil	445 F g^−1^ at 0.5 A g^−1^	1594 m^2^ g^−1^	NA	Zn(CF_3_SO_3_)_2_	217.1 Wh kg^−1^	22.3 kW kg^−1^	90% (3,000; 0.1 A g^−1^)	[91]
		Self-assembly strategy of electrostatic interaction	ZDC/BDC/rGO	Zn foil	236 F g^−1^ at 0.5 A g^−1^	633 m^2^ g^−1^	NA	ZnSO_4_	83.8 Wh kg^−1^	80.7 kW kg^−1^	102% (20,000; 10 A g^−1^)	[92]
	Pore structure optimization	KOH-assisted pyrolysis	MDPC-2	Zn foil	NA	1272.4 m^2^ g^−1^	1.44 cm^3^ g^−1^	Zn(CF_3_SO_3_)_2_	145.5 Wh kg^−1^	45 kW kg^−1^	96.5% (10,000; 10 A g^−1^)	[93]
TMCs	Low intrinsic conductivity	Functional nanomaterial intercalation	BP-Zn-MXene	Zn metal	2.11 F cm^−2^ at 1 mA cm^−2^	NA	NA	PAM-ZnSO_4_	426.3 µWh cm^−2^	NA	94.8% (1,000; NA)	[94]
		Material composite	MAC	3D-VG	633 mAh g^−1^ at 0.1 A g^−1^	NA	NA	Zn(CF_3_SO_3_)_2_	296 Wh kg^−1^	0.4 kW kg^−1^	86.7% (10,000; 10 A g^−1^)	[95]
		Constructing directional macrostructure	LH-Ti_3_C_2_T_X_	Zn foil	NA	NA	NA	Zn(OTf)_2_	NA	NA	80.1% (50,000; 5 A g^−1^)	[96]
		Synergy of doping and defect engineering	N-Ov-ZCO CC	Zn foil	2166.4 F g^−1^ at 1 A g^−1^	63.47 m^2^ g^−1^	NA	KOH + Zn(CH₃COO)_2_	155.68 Wh kg^−1^	10.01 kW kg^−1^	98% (10,000; 10 A g^−1^)	[97]
	Structural collapse	Surface engineering	O-Ti_3_C_2_	Zn/CNT	317.58 F g^−1^ at 0.1 A g^−1^	NA	NA	PAM/ZnSO_4_	384.90 µWh cm^−2^	6.0 mW cm^−2^	96.67% (10,000; 10 mA cm^−2^)	[100]
		Physical intercalation, chemical pore-forming and gas-foaming technology	PM/CNF foam	Zn foil	NA	NA	NA	PAM-ZnSO_4_	103 µWh cm^−2^	2100 µW cm^−2^	80.2% (1,000; NA)	[101]
		Interlayer insertion Material composite	MV-42	MV-42	332.1 F g^−1^ at 1 A g^−1^	16.02 m^2^ g^−1^	NA	PVA/ Zn(CF_3_SO_3_)_2_	39.88 mWh cm^−3^	13.33 W cm^−3^	88.5% (10,000; 3 A g^−1^)	[103]
			ZnF_2_-Ti_3_C_2_T_X_	Zn foil	NA	13.67 m^2^ g^−1^	NA	ZnSO_4_	NA	NA	83.2% (20,000; 4 A g^−1^)	[104]
TMCs		Return processing	ZnPc-400	Zn foil	49.1 F g^−1^ at 0.1 A g^−1^	43.15 m^2^ g^−1^	NA	PVA/ Zn(CF_3_SO_3_)_2_	86.2 Wh kg^−1^	NA	73.4% (100,000; 2 A g^−1^)	[105]
	Slow reaction kinetics	Interface regulation and spatial structure design synergy	TI_3_C_2_T_X_-PPy/Bi_2_S_3_	Zn foil	NA	NA	NA	ZnSO_4_	269.09 Wh kg^−1^	12.95 kW kg^−1^	75.44% (2,000; NA)	[102]
		Material composite	RuO_2_ QDs PCNCs	Zn foil	224 mAh g^−1^	263.4 m^2^ g^−1^	NA	ZnSO_4_	180 Wh kg^−1^	NA	NA (20,000; 10 A g^−1^)	[106]
		Structural optimization	BCNP	rGO-Zn	1601.4 F g^−1^ at 1 A g^−1^	118.55 m^2^ g^−1^	NA	ZnSO_4_	376.5 Wh kg^−1^	30.77 kW kg^−1^	76.4% (10,000; 10 A g^−1^)	[107]

SSA, specific surface area; *P*_v_, pore volume; *E*_d_, energy density; *P*_d_, power density; *C*_R_, capacity retention (%); *C*_n_, cycle number; *C*_d_, current density (A g^−1^); NA, not available

#### Carbon Materials

1. Lack of Specific Surface Area

**Fig. 3:**
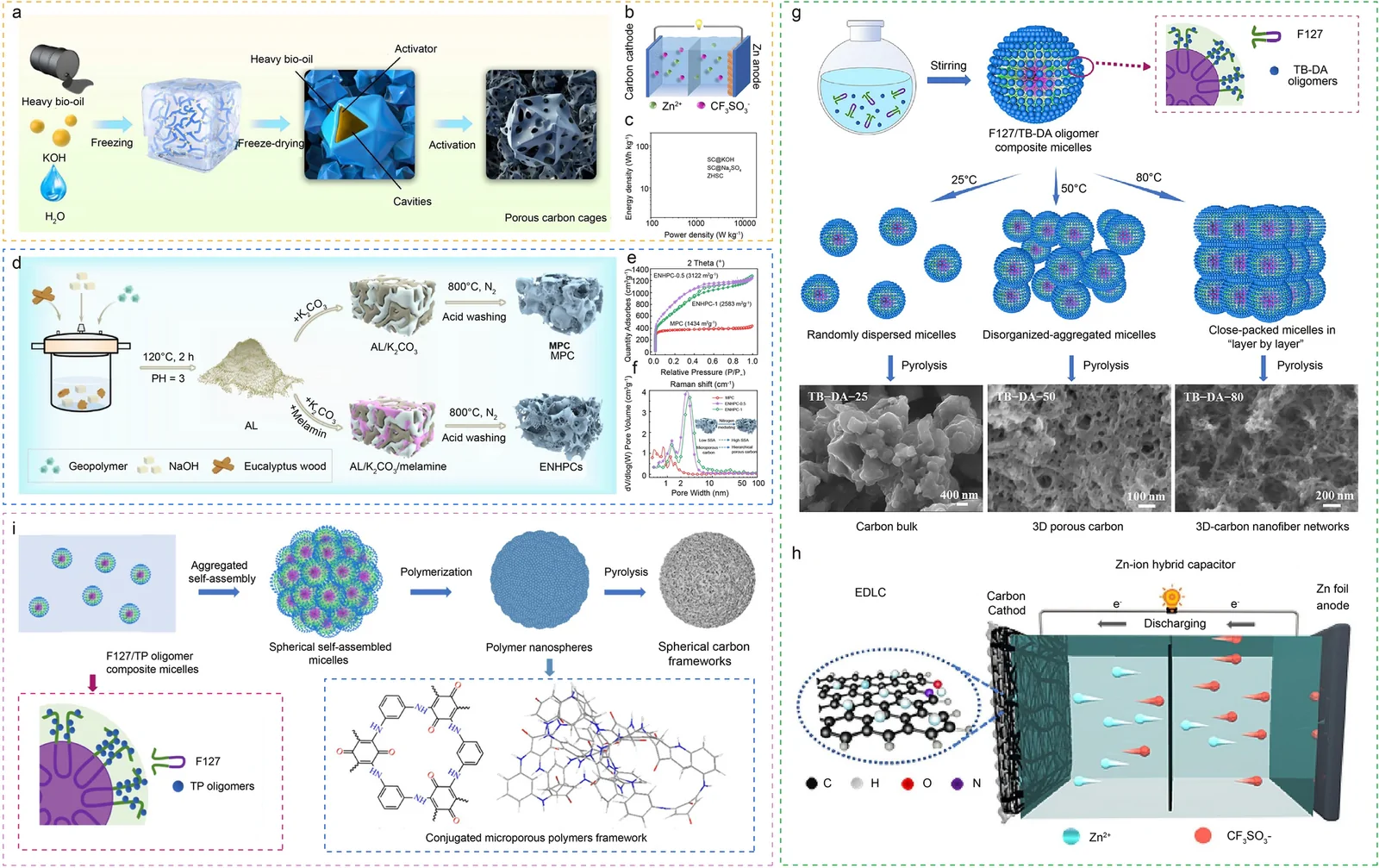
**a** Schematic illustration of the synthesis process of porous carbon (PCC-3). **b** Schematic diagram of the PCC-3//Zn(CF_3_SO_3_)_2_//Zn energy storage system. **c** Energy-power densities of ZICs and SCs assembled by PCC-3 [64]. **d** Schematic illustration of the preparation process of porous carbon materials (ENHPCs). **e** Nitrogen (N_2_) adsorption–desorption isotherms of ENHPCs and MPC. **f** Pore size distributions of ENHPCs and MPC [65]. **g** Schematic illustration of synthesis of nanofiber-interweaved networks. **h** Schematic diagram of the TB-DA-80//Zn(CF_3_SO_3_)_2_//Zn energy storage system [66]. **i** Illustration of synthesis of carbon nanospheres [67]

As an important cathode material for ZICs, the energy storage performance of carbon electrodes is directly related to its SSA. In theory, carbon materials with high SSA often have abundant ionic active sites to provide high EDLC for electrode materials. However, most carbon materials have problems such as tortuous or irregular pore channels, showing limited SSA, which leads to significant depletion of surface-active site density and limits ion storage [61–63]. At present, researchers have used ice template-assisted activation strategy, hydrogen bond micelle template method, and other methods to construct materials with high SSA. Among them, the ice template-assisted activation strategy separates the bio-oil precursor molecules through the ice crystals during the freezing process. The ice crystals sublimate to form large cavities, which opens the internal space of the carbon material and inhibits the structural shrinkage during the carbonization process, thereby constructing an open cage structure [64]. As shown in Fig. 3a, the experiment involved mixing bio-oil with KOH solution, followed by freezing to form an ice crystal framework. The ice crystals were then sublimated through freeze-drying at − 60 °C for 48 h, utilizing the ice crystal occupancy effect to create a porous structure. The material was subsequently carbonized at 700 °C in an argon atmosphere for one hour, followed by acid washing and drying to obtain porous carbon (PCC-1/2/3). Most importantly, the experiment effectively suppressed carbon skeleton shrinkage by adjusting the ice template volume, forming open hollow cavities and thin carbon shells. The hollow structure significantly enhances active site utilization by effectively increasing the SSA and shortening the ion diffusion path. Additionally, ice crystals act as a transport medium for KOH, promoting the formation of microporous structures. As shown in Fig. 3b, c, the PCC-3//Zn device exhibits excellent electrochemical performance when PCC-3 is used as the positive electrode of ZICs, and can maintain a high-energy density (119.02 Wh kg^−1^) at 355.17 W kg^−1^.

In addition, researchers have also found that metal salt hard templates can also effectively regulate the pore structure and SSA of carbon materials. Wang et al. successfully prepared a porous carbon material with a concentrated mesoporous structure, edge nitrogen doping, and high SSA, denoted as ENHPCs, by adopting a melamine-assisted K_2_CO_3_ activation strategy (Fig. 3d) [65]. As shown in Fig. 3e, f, the optimized ENHPC-0.5 material exhibits a high SSA and hierarchical pore structure of 3122 m^2^ g^−1^, and micropores and mesopores synergize to enhance the storage kinetics of Zn^2+^. Electrochemical testing revealed that the material delivered a specific capacitance of 350 F g^−1^ at a current density of 0.1 A g^−1^ and maintained 99.4% of its initial capacity after 10,000 cycles. This work offers a novel perspective for designing advanced carbon materials that balance high SSA, rapid ion transport, and surface functionalization, thereby advancing the development of zinc-ion energy storage devices. It is worth noting that melamine easily decomposes into toxic cyanide gas under high-temperature pyrolysis, posing certain safety hazards.

To reduce the safety hazards that may be caused by toxic gases, Qin et al. used the hydrogen bond micelle template method to prepare an interwoven carbon nanofiber network [66]. As shown in Fig. 3g, the experiment controlled the hydrogen bonds between solvent molecules and the hydrophilic segments of block copolymers by regulating the polymerization temperature, inducing micelle aggregation and self-assembly, and thereby guiding the intertwined formation of carbon nanofiber networks. The study found that the TB-DA-80 sample prepared at 80 °C exhibited a three-dimensional nanofiber interwoven network structure, featuring the highest SSA of 2370 m^2^ g^−1^, pore volume of 2.18 cm^3^ g^−1^, and mesopore ratio of 75.8%, providing abundant surface-active sites and rapid ion transport channels. Electrochemical tests show that TB-DA-80-based ZICs exhibit excellent performance, reaching a maximum energy density of 163 Wh kg^−1^ and a capacity of 90.5% after 400,000 cycles (Fig. 3 h). This study provides a new idea for the design of high-performance carbon-based electrode materials. Similarly, they also prepared conjugated microporous polymer-derived nanospherical carbon frameworks (CNS-2) for ZICs through a hydrogen bond micelle self-assembly strategy (Fig. 3i) [67]. CNS-2 exhibits a monodisperse spherical morphology, abundant tunable porosity, and a layered porous structure, with a mesopore volume fraction as high as 65%, a SSA of 2416 m^2^ g^−1^, and a pore volume of 1.40 cm^3^ g^−1^. These characteristics endow CNS-2 with a greater number of active sites and excellent stability. ZICs assembled with CNS-2 as the cathode exhibit extremely high-energy density (163 Wh kg^−1^) and an exceptionally long-cycle life (maintaining 93% capacity after 200,000 cycles at 20 A g^−1^).

Although the above-mentioned template strategy can significantly improve the energy storage performance of ZICs by regulating the pore distribution and SSA of porous carbon, its three-dimensional (3D) structure still faces the challenge of random and tortuous ion diffusion paths. Fortunately, two-dimensional (2D) carbon materials such as graphene films and porous carbon nanosheets, with their atomic-level thin-layer structures and high conductivity, have great application potential in breaking through the kinetic bottleneck of traditional porous carbon. Recently, Hu et al. designed a self-supporting porous graphene film through a self-assembly strategy, which cleverly broke through this bottleneck [68]. The research team optimized the interlayer pore structure and density of graphene films by modulating the mass ratio of reduced graphene oxide (RGO) to GO. The optimized graphene film exhibits a bulk density of 0.82 g cm^−3^ and a concentrated mesoporous distribution of 3.8 nm, which significantly improves the utilization of active sites. As a ZIC cathode material, the film achieves a volume energy density of 113.1 Wh L^−1^ and a volume power density of 21.2 kW L^−1^ at a thickness of 35 μm, and maintains a cycle stability of 96.4% after 8,000 cycles. Additionally, Liu et al. innovatively used bougainvillea petals as a biomass template and employed carbonization and KOH activation strategies to prepare hierarchical porous carbon nanosheets (CB-3–850) with a “surface porous” structure [69]. The unique 3D porous structure and ultra-thin 2D nanosheet layer characteristics of CB-3–850 confer significant structural advantages. By exposing abundant active sites, CB-3–850 significantly increases SSA, enabling efficient adsorption of Zn^2+^ and SO_4_^2−^. Additionally, the atomic thickness nanosheet layer structure shortens ion diffusion pathways, facilitating rapid ion storage kinetics and higher capacity. CB-3–850 exhibits outstanding electrochemical performance with an ultra-high specific capacity of 239.1 mAh g^−1^ at 0.5 A g^−1^ and an energy density of 213.4 Wh kg^−1^.

2D carbon materials provide unique lamellar structures and abundant active sites, which bring new breakthroughs in the field of electrochemical energy storage. In order to further improve the electrochemical performance of carbon materials, research efforts have focused on utilizing doping modification strategies to optimize the spatial structure and surface chemical properties of carbon materials by introducing heterogeneous atoms, thereby significantly improving their SSA and ion adsorption capacity. For example, Li’s team prepared N-S codoped porous carbon materials using a simple direct activation/annealing method [70]. The doped atoms can serve as active sites, increasing the surface activity of the material. At the same time, the introduction of N and S atoms can enhance the electrolyte wettability of the carbon electrode, leading to an enlargement of the electrochemically active surface area. The prepared carbon material exhibits a monodisperse nanosheet structure, a high SSA (1902.9 m^2^ g^−1^), an appropriate pore size distribution (microporous size approximately 0.93 nm), and abundant N and S functional groups. These characteristics enable it to demonstrate outstanding electrochemical performance (245.8 mAh g^−1^ at 0.2 A g^−1^). Research indicates that the energy storage performance of carbon materials is closely related to their SSA, pore structure, and surface chemical properties. Through innovative methods such as ice template-assisted activation and hydrogen bond micelle self-assembly strategies, researchers have successfully constructed porous carbon materials with hierarchical pore structures and high SSA. Looking ahead, the optimization of carbon-based electrode materials requires multi-dimensional collaborative innovation. Additionally, the development of green synthesis processes is urgently needed, such as replacing toxic precursors with biomass templates and utilizing low-temperature self-assembly technologies to reduce energy consumption.

(2) Limited energy storage mechanisms

Although innovative structural designs can greatly enhance the SSA utilization rate of carbon materials, the lack of active sites means that charge storage still mainly relies on physical electrostatic adsorption mechanisms, making it difficult to exceed the theoretical capacity limit of EDLC. It is worth noting that the heteroatom doping strategy has achieved a breakthrough in energy storage performance through a dual mechanism: On the one hand, doping-induced microstructure reconstruction and pore formation further expand the SSA of carbon materials; on the other hand, heteroatoms as electron donors regulate the electronic structure of the carbon matrix, generate redox-active sites on its surface, and increase the reversible Faradaic reactions on the basis of EDLC [71].

**Fig. 4:**
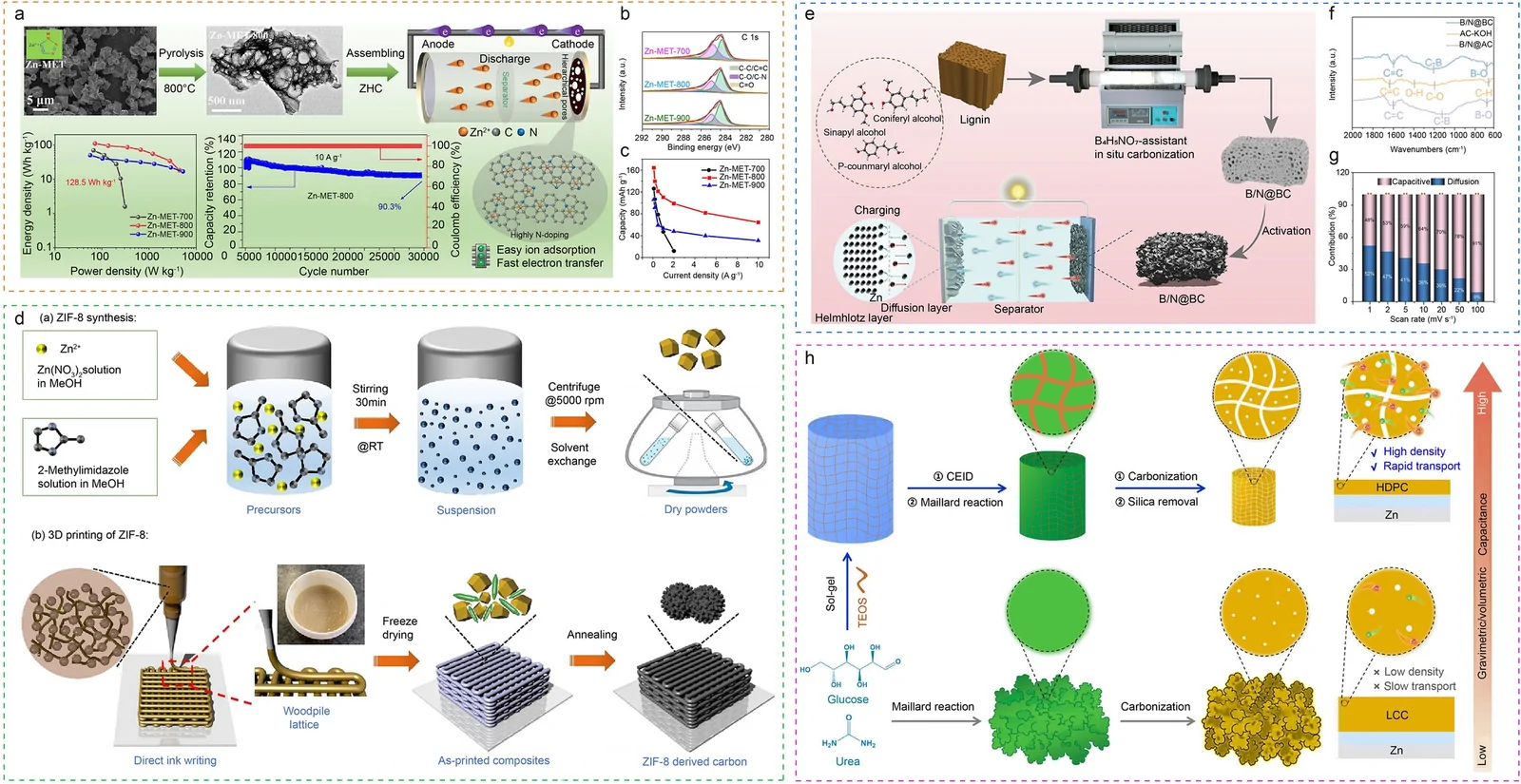
**a** Schematic illustration of the synthesis process of Zn-MET-800 and its corresponding application in ZICs. High-resolution XPS spectra of **b** C 1*s*. **c** Capacities as a function of the current density [72]. Schematic of **d** ZIF-8 powder synthesis process and 3D printing process of ZIF-8 ink [73]. **e** Schematic illustration regarding the preparation of B/N@AC. **f** FT-IR spectra of lignin-derived porous carbon materials. **g** Diffusion-capacitive contribution ration of B/N@AC [74]. **h** Schematic illustration of nanoconfined carbonization route to high-density carbon materials and normal carbonization route to loose carbonaceous char [75]

For example, Jia’s team successfully introduced the pseudocapacitive energy storage mechanism into N-doped porous carbon materials prepared by pyrolysis of metal–organic frameworks (MOF) precursors with high nitrogen content (Fig. 4a) [72]. Jia et al. used Zn-MET as a precursor to obtain Zn-MET-800 with a nitrogen content of up to 16.2 at% by direct pyrolysis, and N mainly exists in the forms of N-6 and N-5. Theoretical calculations reveal that the adsorption energy of nitrogen-doped graphene (− 1.077 eV) is notably greater in comparison to that of pure graphene (− 0.845 eV). XPS analysis showed that the presence of C–O/C–N and C=O bonds contributed additional pseudocapacitance through surface redox reactions (Fig. 4b). Electrochemical tests show that ZIC achieves a high capacity of 164.2 mAh g^−1^ at 0.1 A g^−1^, and maintains an excellent rate performance of 64.4 mAh g^−1^ at 10 A g^−1^. This can be ascribed to the synergistic effect exerted by N-doped enhanced active sites and hierarchical pores (Fig. 4c). Furthermore, as shown in Fig. 4d, Catarineu et al. successfully prepared N-doped carbon materials for high-performance ZICs through the 3D printing and pyrolysis of zinc-based metal–organic frameworks (ZIF-8) [73]. After pyrolysis, ZIF-8 was converted into N-doped graphitized carbon, and its hierarchical pores (micropore–mesoporous–macropore) and N doping synergistically improved the adsorption and diffusion efficiency of Zn^2+^. Furthermore, the doped N provides additional pseudocapacitance contribution through the coordination interaction with Zn^2+^ (Zn-N) and the reversible redox reaction (C=N ↔ C–N). This strategy of accurately regulating nitrogen doping and pore structure through MOF precursors provides a new idea for introducing pseudocapacitive energy storage mechanism, but the high cost and complex synthesis process of MOF precursors limit their large-scale application.

Biomass is an ideal substitute because of its rich heteroatom species and low-cost. Cao et al. used lignin to prepare B/N codoped hierarchical porous carbon by in situ KOH activation and carbonization assisted by NH_4_HB_4_O_7_, and successfully introduced an efficient pseudocapacitive energy storage mechanism (Fig. 4e) [74]. The introduction of B makes the existence of C–B, C=N, and B–O bonds in the material, providing additional active sites to promote the reversible adsorption/desorption reaction of Zn^2+^ (Fig. 4f). Electrochemical tests show that B/N@AC exhibits a high capacitance of 330 F g^−1^ in 2 M ZnSO_4_ electrolyte, and the pseudocapacitance ratio reaches 91% at a scan rate of 100 mV s^−1^, which is significantly better than that of single doped material (Fig. 4 g). The use of a B/N codoping strategy in biomass-based carbon materials provides a green pathway for introducing pseudocapacitive energy storage mechanisms, but due to limitations in material density, there is still room for improvement in volumetric energy density. In contrast, Lu et al. utilized nanoconfined carbonization technology to prepare highly porous carbon (HDPC-1) with a density as high as 0.78 g cm^−3^ through the synergistic action of glucose/urea precursors and SiO_2_ templates (Fig. 4h) [75]. Although HDPC-1 mainly relies on hierarchical pores to achieve efficient Zn^2+^ adsorption, the residual N forms additional active sites by changing the electronic structure of the carbon skeleton, thereby introducing pseudocapacitive contributions. This strategy of balancing porosity and density by nanoconfinement regulation not only breaks through the bottleneck of volumetric energy density (453 F g^−1^), but also optimizes the surface chemical environment through the synergistic effect of residual nitrogen, which provides a new idea for improving Zn^2+^ storage.

Furthermore, halogen element fluorine doping and sulfur doping with a large atomic radius have gradually entered the researchers’ field of vision. Recently, Samartzis et al. used laser-assisted preparation of fluorine-doped graphene (LEST-FG) to successfully introduce a pseudocapacitive energy storage mechanism enabled by the synergistic effect between C–F moieties and surface hydroxyl groups [76]. XPS analysis shows that F exists in the carbon skeleton in the form of C–F, and its high electronegativity induces local charge polarization and enhances the chemical adsorption of Zn^2+^. At the same time, density functional theory (DFT) calculations show that the hydroxyl group (-OH) near F forms a coordination structure (C–F · · Zn^2+^) with Zn^2+^ through hydrogen bonds, which significantly reduces the adsorption energy barrier compared with pure graphene (− 1.25 vs. − 0.84 eV), thereby increasing the pseudocapacitance contribution. In the experiment, the LEST-FG-74 electrode exhibits a discharge capacity of 19.5 μAh cm^−2^ and an energy density of 10.93 μWh cm^−2^ in 1 M Zn(CH_3_COO)_2_ electrolyte, which is 55% higher than that of the undoped sample. This fluorine doping strategy through laser-induced synchronous synthesis and transfer provides a new idea for the design of high-efficiency pseudocapacitive materials. In recent years, it has been found that sulfur-based functional groups provide a new reaction path for Zn^2+^ storage owing to their distinctive electronic structure and strong coordination ability. S with larger atomic radius and lower electronegativity endows the material with higher redox activity. For example, Cataldo Valentini et al. successfully introduced an efficient pseudocapacitive energy storage mechanism by doping the S element, thereby synergistically introducing the thiol group (–SH) and the oxygen functional group (OFGs) [77]. XPS analysis showed that -SH on the surface of the material accounted for 74%, and -SH formed a strong coordination bond with Zn^2+^, which provided additional Faraday capacity through a reversible adsorption/desorption reaction (–SH + Zn^2+^ ↔ -S-Zn + H^+^). In the experiment, rGOSH electrodes displayed an exceptional maximum energy density of 187.6 Wh kg^−1^ and power density of 48.6 kW kg^−1^.

**Fig. 5:**
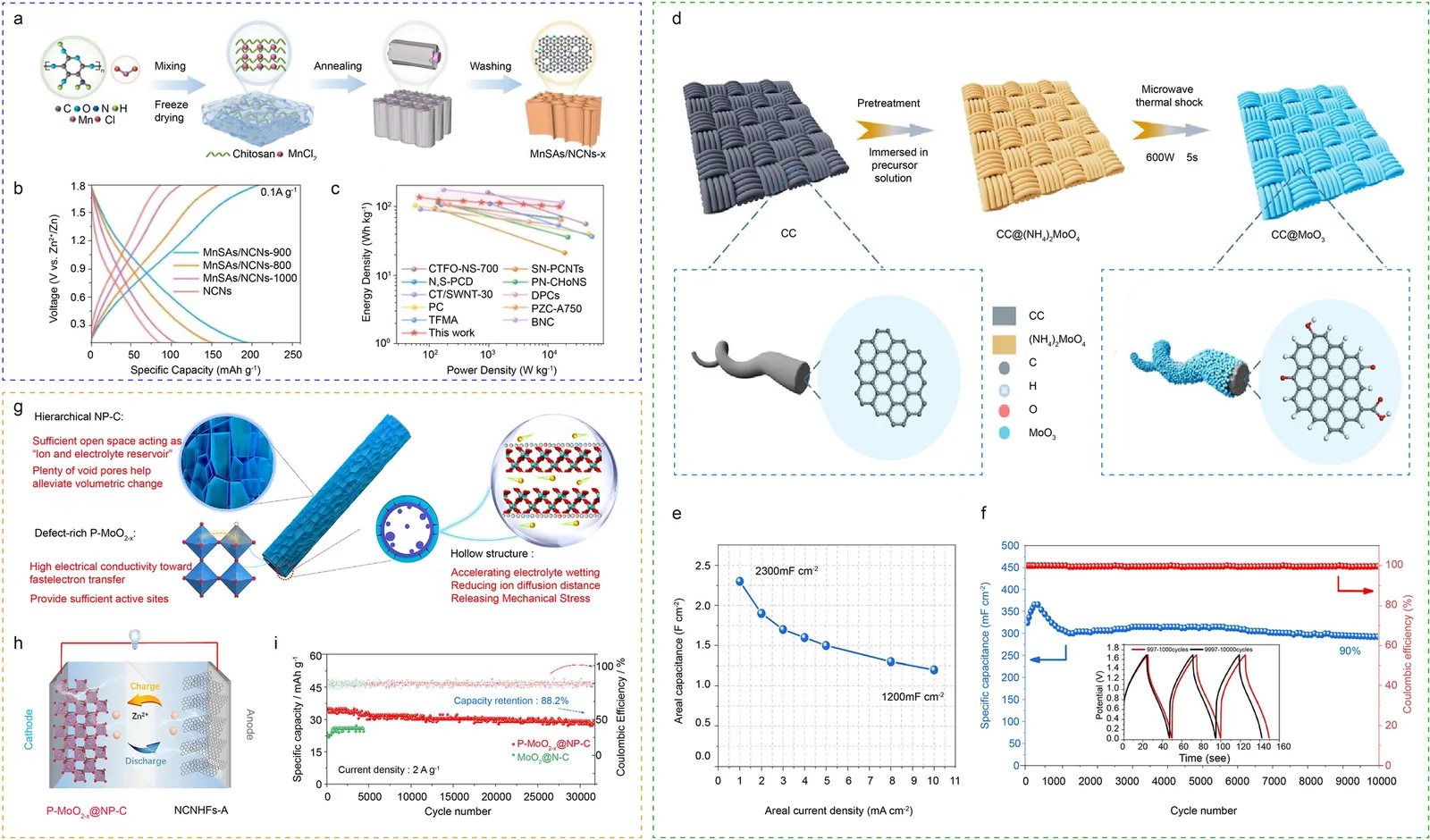
**a** Schematic illustration of the formation process of MnSAs/NCNs-x. **b** GCD curves at 0.1 A g^−1^ of MnSAs/NCNs-x and NCNs. **c** Ragone plots of MnSAs/NCNs-900-based ZICs and other ZICs [78]. **d** Schematic diagram of the preparation of AMCTS-CC@MoO_3_. **e** Rate performance of AMCTS-CC@MoO_3_ as the current density increases from 1 to 10 A cm^−2^. **f** Cyclic performance of the assembled ZIC [79]. **g** Structural advantages of P-MoO_2_@N–C for charge storage. **h** Schematic illustration of N–C–A//P-MoO_2-X_@NP-C ZICs device. **i** Relationship between specific capacity and current densities of N–C–A//P-MoO_2-X_@NP-C ZICs [80]

In addition, in ZICs, metal single-atom anchoring also provides an innovative path for the introduction of pseudocapacitance into carbon-based materials. For example, manganese atom-anchored nitrogen-doped porous carbon nanosheets (MnSAs/NCNs) have successfully introduced an efficient pseudocapacitive energy storage mechanism via the synergistic interaction between atomically dispersed active sites and the hierarchical pore structure. Zhu et al. used Mn to insert in the carbon skeleton in an atomically dispersed Mn-N configuration, and directly contributed Faradaic pseudocapacitance through a reversible Mn^2+^/Mn^3+^ redox reaction (Fig. 5a) [78]. At the same time, the strength of C–O–Zn changes during the charge–discharge process, which indicates that there is a typical chemical adsorption between the C–O/C=O group and Zn^2+^. In addition, the change in N–O bond is similar to that of C–O and C=O bonds. The above findings indicate that oxygen functional groups play an active role in Zn^2+^ storage. By comparing the manganese-free samples (MnSAs/NCNs-w), it was found that the contribution of pseudocapacitance disappeared after the removal of Mn, which strongly confirmed that the manganese single atom was the core source of pseudocapacitance. As shown in Fig. 5b, c, the material exhibits a high specific capacity reaching 203 mAh g^−1^ at 0.1 F g^−1^, and the ZIC based on the MnSAs/NCNs-900 cathode demonstrates a high-energy density of 138 Wh kg^−1^ at 68 W kg^−1^. The metal single-atom anchoring strategy opens up a multi-dimensional path for the performance improvement of ZICs through a completely different mechanism, revealing the deep coupling law of electron transfer and ion adsorption in the pseudocapacitance process.

The above strategies improve the capacitance performance by changing the intrinsic properties of the material. However, the modification of a single material is sometimes difficult to meet the needs of high-performance energy storage devices. Therefore, researchers have extended the research perspective to carbon-based composite systems, and introduced pseudocapacitive energy storage mechanisms by constructing carbon/metal oxides or carbon/conductive polymer heterogeneous interfaces to further improve electrochemical energy storage performance.

For example, in the study of Chai et al. the α-MoO_3_/carbon cloth composite (CC@MoO_3_) synthesized in situ by microwave carbon thermal shock method was used as the anode material of ZICs (Fig. 5d) [79]. The pseudocapacitive energy storage mechanism of CC@MoO_3_ is mainly due to the multivalent redox reaction of Mo, and the valence change of Mo (Mo^6+^/Mo^4+^) provides a reversible redox reaction. At the same time, the layered orthorhombic structure of α-MoO_3_ allows Zn^2+^ to rapidly intercalate/deintercalate between layers. Electrochemical testing revealed that AMCTS-CC@MoO_3_ exhibited a specific capacitance as high as 2,300 mF cm^−2^ within the potential window of − 0.8 to 0.1 V. Additionally, the assembled ZICs achieved a high areal energy density of 321 μWh cm^−2^ at an operating voltage of 1.7 V and demonstrated excellent stability after 10,000 cycles (Fig. 5e, f). Furthermore, for ZICs, the design strategy for metal oxide composite carbon materials is evolving from single-defect regulation to multi-dimensional synergistic optimization. Taking P-MoO_2-X_@NP-C as an example, Zhang’s team synthesized materials with unique structures via the Kirkendall effect and combined them with P and N-doped carbon sheet encapsulation (Fig. 5g), significantly optimizing the material’s electronic transport and reaction kinetics [80]. XPS analysis indicates that P doping converts some high-energy Mo–O bonds into low-energy Mo-P bonds, reducing the oxidation state of Mo (Mo^4+^ → Mo^3+^), thereby enhancing surface reactivity. Meanwhile, oxygen vacancies expand the interlayer spacing, providing more ion diffusion pathways, which facilitates the rapid migration of Zn^2^⁺. Electrochemical testing revealed that the P-MoO_2-X_@NP-C-assembled ZICs exhibited a capacity retention rate of up to 88.2% after 32,000 cycles at 2 A g^−1^, exhibiting an energy density as high as 43.8 Wh kg^−1^, highlighting the material’s stability and structural advantages at high rates (Fig. 5h, i). This study combines defect engineering with structural design to integrate the intrinsic pseudocapacitance of P-MoO_2-X_ with the conductivity of the carbon layer, providing an efficient energy storage strategy for ZICs.

Although composite strategies combining metal oxides with carbon materials have significantly enhanced the performance of ZICs through defect engineering and structural design, polymers also represent a highly promising class of materials. Li et al. achieved this by in situ electropolymerizing organic compounds (poly-8-amino-2-naphthol) on laser-induced graphene, resulting in ZICs with outstanding electrochemical performance [81]. Specifically, the C=N and C=O groups in poly-8-amino-2-naphthol can undergo coordination reactions with Zn^2+^ and H^+^, and these functional groups can undergo reversible Faradaic reactions during charging and discharging. Additionally, the hydrogen bond network between polymer chains facilitates proton conduction, further enhancing the performance of ZICs. Electrochemical testing shows that the composite electrode exhibits a high specific capacity of 308 mAh g^−1^ at a current density of 0.1 mA cm^−2^, and these ZICs can stably endure 10,000 cycles at 5 mA cm^−2^. This study successfully overcomes the performance limitations of traditional metal oxide-based ZICs through the synergistic design of carbon materials and polymers, providing new insights for the development of high-energy density, long-lifetime energy storage devices.

Researchers have successfully introduced pseudocapacitive energy storage mechanisms through strategies such as heteroatom doping, metal single-atom anchoring, and composite system construction, breaking through the theoretical limits of electric double-layer capacitance. The core of these strategies lies in the synergistic integration of physical adsorption and Faradaic reactions via atomic-level structural regulation and interface engineering, which significantly enhances the intrinsic activity and reaction kinetics of the materials [82]. Building on the aforementioned strategies, the targeted grafting of molecules with well-defined redox activity onto carbon scaffolds provides a novel molecular engineering pathway for capacity enhancement. This approach achieves Faradaic charge storage that surpasses traditional doping effects by introducing organic functional groups capable of undergoing highly efficient and reversible reactions. For instance, the in situ electropolymerization of poly-8-amino-2-naphthol on LIG enables reversible coordination reactions between its C=N and C=O groups with Zn^2+^/H^+^, resulting in an electrode specific capacity of 308 mAh g^−1^ [81]. Similarly, the modification of graphene with thiol groups (–SH) establishes a reversible conversion mechanism of –SH + Zn^2+^ ↔ -S-Zn + H^+^, increasing the specific capacitance to 540 F g^−1^ and the energy density to 187.6 Wh kg^−1^ [77]. Leveraging the well-defined and controllable reaction pathways of organic functional groups, such strategies open up a universal and flexible route for constructing high-capacity cathodes for ZICs [83].

Despite significant progress, issues such as material cost, long-term cycling stability, and volumetric energy density remain critical bottlenecks to be addressed for practical applications. Future research needs to integrate interdisciplinary technologies to drive the coordinated development of ZICs toward higher energy density, higher power density, and longer lifetimes.

(3) Slow Diffusion Kinetics

**Fig. 6:**
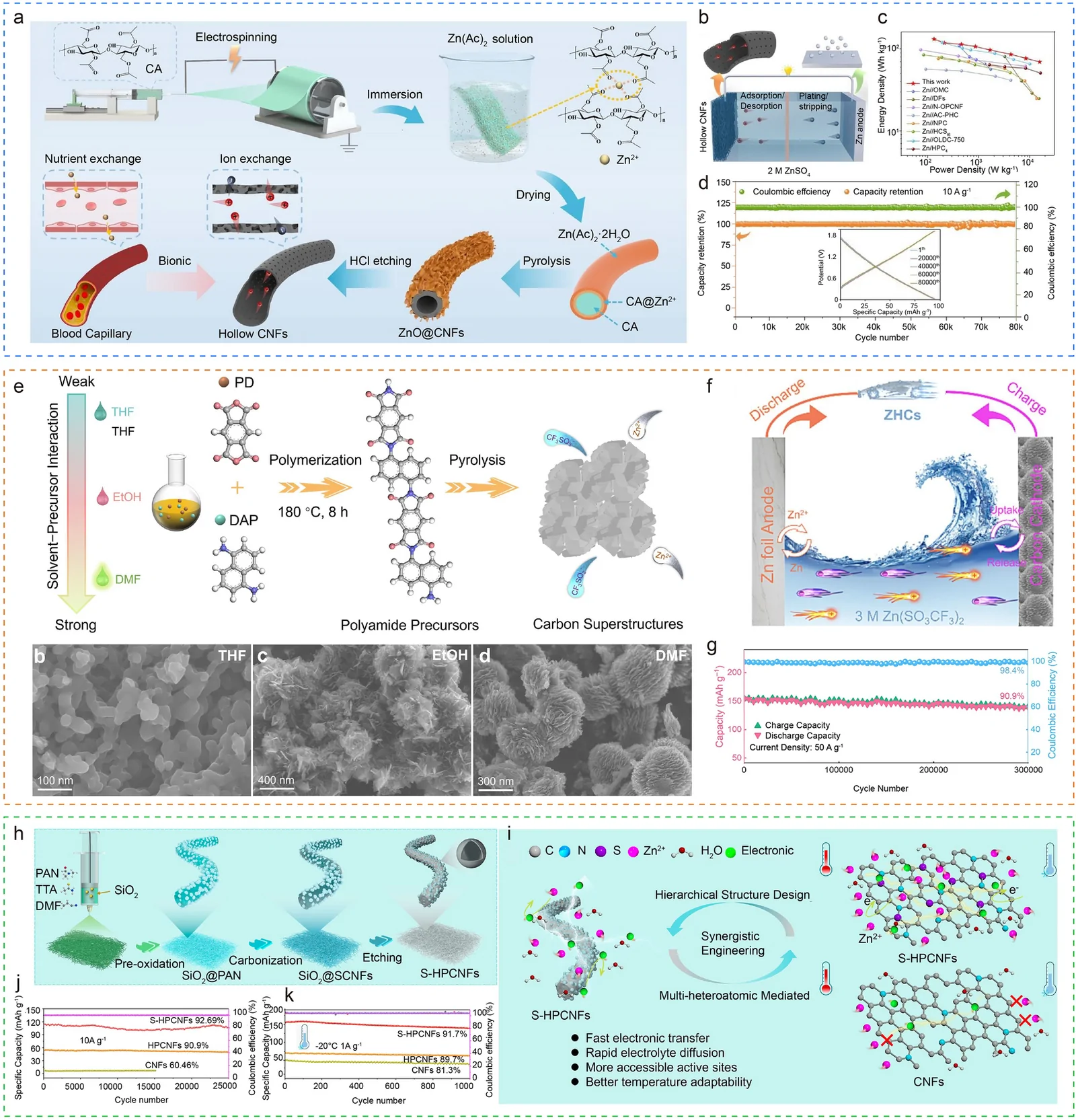
**a** Schematic illustration of the synthesis route of the CNF-Zn-800. **b** Schematic diagram of the hollow CNFs//ZnSO_4_//Zn energy storage system. **c** Ragone plot of CNF-Zn-800 based ZIC. **d** Cycle performance of CNF-Zn-800 based ZIC at 10 A g^−1^ for 80,000 cycles [84]. **e** Formation process of carbon superstructures. **f** Schematic diagram of the Carbon//Zn(SO_3_CF_3_)_2_//Zn energy storage system. **g** Cyclability and CE of ZICs [86]. **h** Schematic illustration of the synthesis process of S-HPCNFs. **i** Schematic of the merits of S-HPCNFs material compared with CNFs material. **j** Cycling stability and the CE at 10 A g^−1^ of the ZIC. **k** Cycling performance at 0.1 A g^−1^ of S-HPCNFs, HPCNFs, and CNFs electrode at a temperature of − 20 °C [88]

After the successful introduction of the pseudocapacitance mechanism by chemical modification, carbon-based materials have seen a significant improvement in their energy storage performance. However, it is not desirable to rely solely on surface functionalization. Because of the large diameter of hydrated zinc ions ([Zn(H_2_O)_6_]^2+^), the pore structure of carbon materials is often difficult to achieve effective ion transport and space adaptation, so poor space utilization hinders the porous carbon cathode to provide high specific capacitance. Therefore, by precisely regulating the pore size distribution and hierarchical structure of carbon materials, it has become a key path to break through the contradiction between current energy density and rate performance. Recently, researchers have proposed a construction strategy of thin-walled hollow carbon nanofibers (CNFs) based on the design of bionic capillary structure. This strategy employs a coordination-pyrolysis synergistic method, first preparing cellulose acetate nanofibers via electrospinning, followed by the generation of a core–shell framework through the coordination of Zn^2+^ with the oxygen functional groups of the cellulose molecular chains. After high-temperature pyrolysis, the ZnO template is removed via acid etching, ultimately yielding CNFs with hierarchical pores (Fig. 6a). Yang et al. used ZnO templates to create pores in situ, forming mesoporous structures (1.47 nm) significantly larger than [Zn(H_2_O)_6_]^2^⁺, thereby reducing desolvation resistance [84]. Additionally, DFT calculations showed that its ion insertion energy barrier was reduced by 67% compared to traditional microporous carbon, avoiding kinetic limitations during the desolvation process. Based on these advantages, ZICs assembled with CNFs as the cathode provided a battery-qualified energy density of 132.8 Wh kg^−1^ and excellent cycling stability (Fig. 6b–d). Yang et al. successfully achieved precise size matching of [Zn(H_2_O)_6_]^2+^ through a coordination-pyrolysis synergistic method to prepare thin-walled hollow carbon nanofibers, reducing the desolvation energy barrier to 0.3 eV and increasing the diffusion coefficient to 2.3 × 10^–10^ cm^2^ s^−1^. Recently, Hu et al. proposed a hydrothermally coupled double salt activation strategy to construct an oxygen-doped carbon cathode material rich in ~ 1 nm confined pores [85]. For the first time, through DFT simulation and experimental verification, it is clear that the optimal matching between the pore size of 1 nm and the size of [Zn(H_2_O)_6_]^2+^ can maximize the spatial confinement effect and achieve orderly and efficient ion transport and storage. When the pore size is greater than 1.72 nm, it will lead to excessive shielding effect. When the pore size is less than 0.86 nm, additional desolvation energy is required, which is not conducive to performance improvement. In addition, abundant oxygen functional groups and structural defects promote the reversible redox reaction of Zn^2+^, further improving the capacitance behavior. Based on the synergistic mechanism of space confinement and defect dominance, the as-prepared ZICs achieve an energy density of 135.5 Wh kg^−1^, a power density of 24.00 kW kg^−1^, and excellent performance with a capacity retention rate of 108.2% after 150,000 cycles. This work provides a clear pore size regulation strategy and mechanism understanding for the design of high-performance ZICs carbon cathodes, and emphasizes the key role of ion-pore size matching and surface chemical modification in improving the comprehensive performance of zinc-ion capacitors.

However, the design with a single pore size still has limitations when facing complex electrolyte environments, such as dynamic changes in ion size in high-concentration electrolytes, which may lead to a decrease in transport efficiency. On this basis, Huang et al. proposed a design strategy for solvent-guided heterodiatomic carbon superstructures [86]. This strategy optimizes the thermodynamic dissolution and growth kinetics of polymer intermediates by regulating the interaction between solvents (THF, ETOH, and DMF) and polyimide, and finally constructs a spherical carbon superstructure with 1–4 nm gradient pore channels (micropores 1–2 nm, mesopores 2–4 nm) (Fig. 6e). In addition, DFT calculations show that the insertion energy barrier of [Zn(H_2_O)_6_]^2+^ is significantly lower than that of the traditional 7 Å micropore, which avoids the kinetic limitation of the desolvation process. Meanwhile, the codoping of N and P enhances the chemical adsorption of [Zn(H_2_O)_6_]^2+^ through synergistic effects and contributes additional capacity by forming C-N-Zn coordination bonds. The synergistic design of gradient pore channels and doped atoms not only offers size-matching advantages but also overcomes the limitations of single pore sizes. This design enables ZICs to achieve both high-energy density (145 Wh kg^−1^) and ultra-long-cycle life (90.9% capacity retention after 300,000 cycles at 50 A g^−1^) (Fig. 6f, g). Similarly, researcher Peng proposed a synergistic design strategy for hierarchically ordered porous carbon (PN-HOPC). Peng et al. used a 3D ordered polystyrene template combined with ZIF-8 carbonization to construct a synergistic structure of macropores, mesopores, and micropores, in which mesopores accounted for 32.2%, directly matching the size of [Zn(H_2_O)_6_]^2+^ and avoiding the kinetic limitations of the desolvation process [87]. Additionally, the codoping of P and N introduces abundant active groups, which enhance capacity through reversible chemical adsorption. This design enables ZICs to achieve a high-energy density of 169.5 Wh kg^−1^, and maintains 99.3% capacity after 60,000 cycles, providing a new approach for pore-chemical synergistic optimization for [Zn(H_2_O)_6_]^2+^ adaptation.

Notably, Peng et al. achieved excellent Zn^2+^ storage performance at room temperature through the design of layered ordered porous carbon, but their low-temperature performance has not been thoroughly investigated. Therefore, Liang et al. further proposed N/S codoped porous hollow carbon nanofibers to break through the bottleneck of low temperature through a dual strategy (Fig. 6 h, i) [88]. DFT calculations confirm that the S site and N site form a synergistic adsorption center, which stabilizes the solvation layer of [Zn(H_2_O)_6_]^2+^ more effectively than single N doping. Secondly, the hollow fiber structure creates ultra-short ion diffusion paths of less than 50 nm, combined with hierarchical pores. Finally, as shown in Fig. 6j, k, the S-HPCNFs cathode exhibits a high specific capacity of 221.5 mAh g^−1^ at 0.1 A g^−1^ and maintains a capacity retention of 92.69% after 25,000 cycles. Even at the extremely low temperature of − 20 °C, its capacity retention remains at a high level of 91.7%, demonstrating excellent low-temperature adaptability. These findings on the relationship between the diameter of [Zn(H_2_O)_6_]^2+^ and the pore structure of carbon materials represent a progressive breakthrough from single-structure optimization to multi-dimensional collaborative design. At the same time, they reveal key principles in carbon material design: Precise matching of pore size is central to overcoming the transport kinetics of [Zn(H_2_O)_6_]^2+^, while synergistic doping with heteroatoms is key to enhancing chemical adsorption capacity.

Carbon-based materials hold great promise for applications in energy storage. By precisely controlling the pore structure, chemical composition, and surface properties of these materials, their energy storage performance can be significantly enhanced. Future research should continue to explore new material design strategies and gain a deeper understanding of ion transport and storage mechanisms to achieve energy storage devices with higher performance and energy density. In addition to structural energy density, superior rate performance, and longer cycle life, this requires interdisciplinary collaboration among materials science, chemistry, and physics, as well as a close integration of experimental research and theoretical calculations to drive the further development and practical application of carbon-based energy storage materials.

#### Organic Framework Materials

In recent years, with advances in materials design theory and synthesis technology, porous crystal materials represented by metal–organic frameworks (MOFs) and covalent organic frameworks (COFs) have become a hot topic of research for breaking through the performance bottleneck of carbon materials. This is due to their precisely controllable crystal topological structures, ultra-high porosity, and abundant redox-active sites. Nevertheless, due to the poor intrinsic conductivity of organic framework materials, the charge transfer efficiency is limited, which seriously restricts its large-scale application in the field of energy storage. In particular, problems such as insufficient rate performance and poor cycle stability are shown in supercapacitors and batteries that require high-power output. In recent years, researchers have provided a new way to break through the bottleneck of application through strategies such as metal doping, pore engineering and construction of heterojunctions. For instance, researchers used Cu-doped Zn-MOF precursors, which were subjected to high-temperature pyrolysis to obtain ordered mesoporous N-doped carbon nanosheets (Zn_1_Cu_X_MC) [89]. The introduction of Cu not only stabilized the MOF framework but also induced the directed dissociation of ligands to form uniform slit-like mesopores. This structural optimization strategy significantly reduces the charge transport resistance, while the open pores facilitate the rapid diffusion of Zn^2+^. Additionally, the synergistic effect of the conductive network of Cu nanoparticles and N-doped carbon plays an indispensable role in enhancing the material’s conductivity. As a cathode assembly in ZICs, Zn_1_Cu_X_MC achieves a high capacity of 134 mAh g^−1^ at 0.5 A g^−1^ and demonstrates excellent cycling stability.

**Fig. 7:**
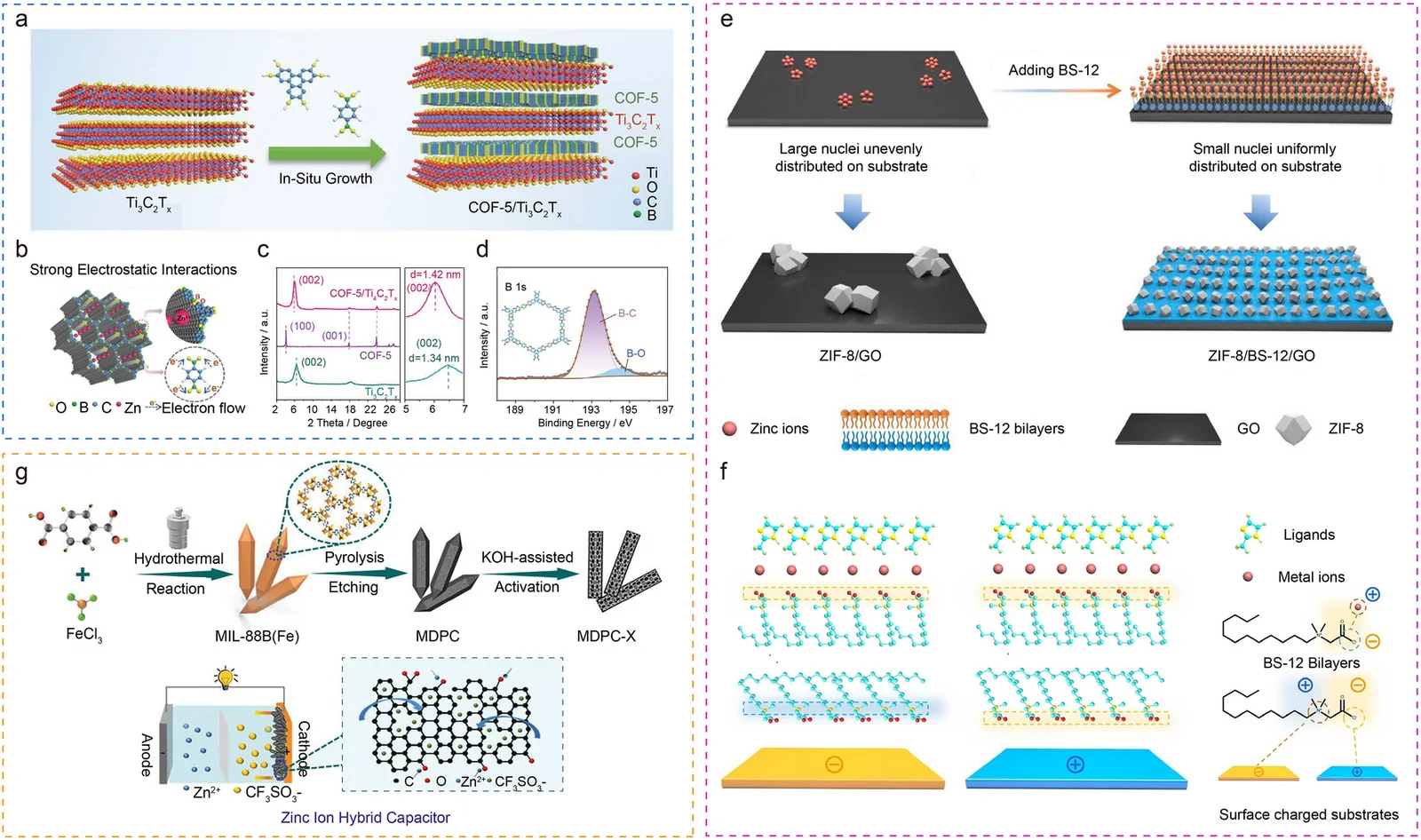
Schematic illustration of **a** preparation process and **b** strong electrostatic interactions with Zn^2+^ of COF-5/Ti_3_C_2_T_X_. **c** XRD patterns of COF-5, Ti_3_C_2_T_X_, and COF-5/Ti_3_C_2_T_X_. **d** B 1*s* XPS spectrum of COF-5/Ti_3_C_2_T_X_ [90]. **e** Schematic illustration of the bidirectional electrostatic interaction-induced coating of single-layer ZIF-8 on GO sheets. **f** Schematic illustration of the mechanism for fabricating the MOF/BS-12/substrate composites [92]. **g** Schematic illustration of fabrication process of the MDPC-X and the construction of ZIC device [93]

In addition to structural modification through metal doping and pore engineering, the formation of heterojunctions between MOF/COF and highly conductive 2D materials can further optimize charge transport kinetics through interface engineering, thereby improving charge transport efficiency. As shown in Fig. 7a, b, Xie et al. inserted porous COF-5 into the interlayer of Ti_3_C_2_T_X_ through in situ polymerization to form a heterojunction cathode material [90]. From the results of XRD and XPS analysis (Fig. 7c, d), the elements in COF-5 doped the conjugated skeleton, thus expanding the interlayer spacing of MXene. This expansion may lead to a larger exposed surface area and more interlayer nanochannels, promoting reversible Zn^2+^ storage. In addition, the O, B and cations within the material showed strong electrostatic interaction, which significantly enhanced the adsorption of Zn^2+^. The conductivity of COF is greatly improved, which is mainly attributed to the addition of highly conductive MXene. This design allows the device to exhibit a high reversible capacity of 524 mF cm^−2^.

The skeletal stability of organic framework materials depends on the strength of the coordination bonds between metal nodes and organic ligands. However, the repeated insertion/extraction of Zn^2+^ during electrochemical cycling causes significant volume expansion, leading to lattice distortion and localized stress accumulation, which in turn results in microcracks or pore collapse. Besides, MOFs are prone to ligand dissolution or metal node hydrolysis in acidic electrolyte, which further weaken the structural integrity. It is worth noting that the structural collapse of organic framework materials will lead to reduced exposure of active sites and decreased porosity, thus weakening the ion storage capacity. At present, researchers often choose strategies such as site engineering and construction of composite materials to solve this problem. For instance, Zhang et al. used a cation-driven self-assembly process to uniformly coat a small amount of Ti_3_CN nanosheets on the surface of COF, forming a stable core–shell structure [91]. The introduction of Ti_3_CN nanosheets not only enhances the material’s conductivity but also prevents structural collapse of COF during charging and discharging through the physical support provided by the 2D nanosheets. This structural design preserves the pores of the COFs, increases the SSA and exposure of active sites, thereby significantly enhancing the electrochemical performance of the ZICs. The constructed CM-P//Zn ZICs exhibits a capacity as high as 260 mAh g^−1^ (0.1 A g^−1^) in 3 M Zn(CF_3_SO_3_)_2_ solution, while achieving a power density of 22.3 kW kg^−1^.

Leng et al. proposed a self-assembly strategy through electrostatic interaction to solve the problems of easy collapse and few surface-active sites of traditional organic framework materials [92]. Specifically, by leveraging the bidirectional electrostatic interactions provided by zwitterionic surfactants, single-layer nanoscale MOFs are uniformly coated onto various substrates to form stable core–shell structures. This strategy not only ensures the oriented and uniform coating of MOFs on the substrate but also prevents self-nucleation and stacking of MOFs, thereby avoiding structural collapse. This strategy also enhances the material’s conductivity by constructing a continuous carbon network and reducing graphene oxide sheets within the material, forming a “dual-conductivity network”. Additionally, through high-temperature carbonization treatment, the ZIF-8/BS-12/GO precursor is carbonized at 800 °C, forming a carbon composite material with a hierarchical pore structure, further enhancing the material’s structural stability (Fig. 7e, f). The combined effect of these strategies makes the prepared materials exhibit high specific capacity, excellent rate performance, and ultra-long-cycling stability in ZICs. Through the core–shell structure design and electrostatic self-assembly strategy, although the stability and active site utilization of organic framework materials have been significantly improved, how to further optimize the pore structure and achieve large-scale preparation still needs to be explored. Hence, researchers proposed using KOH-assisted pyrolysis to directly convert MOF precursors into carbon-based materials (MIL-88B) with multi-level pore structures [93]. This strategy precisely controls the mixing ratio of MOF and KOH, leveraging the etching and activation effects of KOH at high temperatures to form microporous–mesoporous synergistic open channels within the carbon framework (Fig. 7g). This approach avoids the pore collapse caused by the decomposition of organic ligands during traditional pyrolysis processes. Additionally, the introduction of FeCl_3_ as a cross-linking agent enhances the mechanical strength of the carbon framework and mitigates volume expansion issues caused by the repeated insertion/extraction of Zn^2+^. This approach enhances the SSA of MIL-88B-derived carbon rods to 1272.4 m^2^ g^−1^, forming a multi-level structure with micro-, meso-, and macropores. The abundant C–O–Zn active sites on the material’s surface enhance Zn^2+^ storage capacity through chemical adsorption, enabling ZICs to achieve 323.4 F g^−1^ (0.5 A g^−1^) and an energy density of 145.5 Wh kg^−1^ in a 2 M Zn(CF_3_SO_3_)_2_ electrolyte.

In summary, the organic framework material provides an innovative path for the performance breakthrough of zinc-ion energy storage devices by virtue of its crystal engineering advantages. Through strategies such as metal doping, heterostructure construction and hierarchical pore design, the conductivity, structural stability, and active site utilization of the material have been significantly improved. Future research should focus on further optimizing the pore structure, exploring new composite materials, and achieving large-scale preparation to promote the wider utilization of organic framework materials for energy storage applications.

#### TMCs

As the core component of ZICs, the performance of the cathode material directly determines the energy storage capacity of the device. Among many candidate materials, TMCs are considered as promising capacitive cathode materials due to their high theoretical specific capacity, multivalent redox characteristics, and structural tunability. However, the intrinsic conductivity of these materials is generally low, which seriously restricts their practical application. In response to this challenge, researchers have systematically improved the charge transfer efficiency of TMCs through multi-dimensional strategies such as material hybridization strategy, structural engineering design, and doping, laying a foundation for their performance breakthroughs in ZICs.

**Fig. 8:**
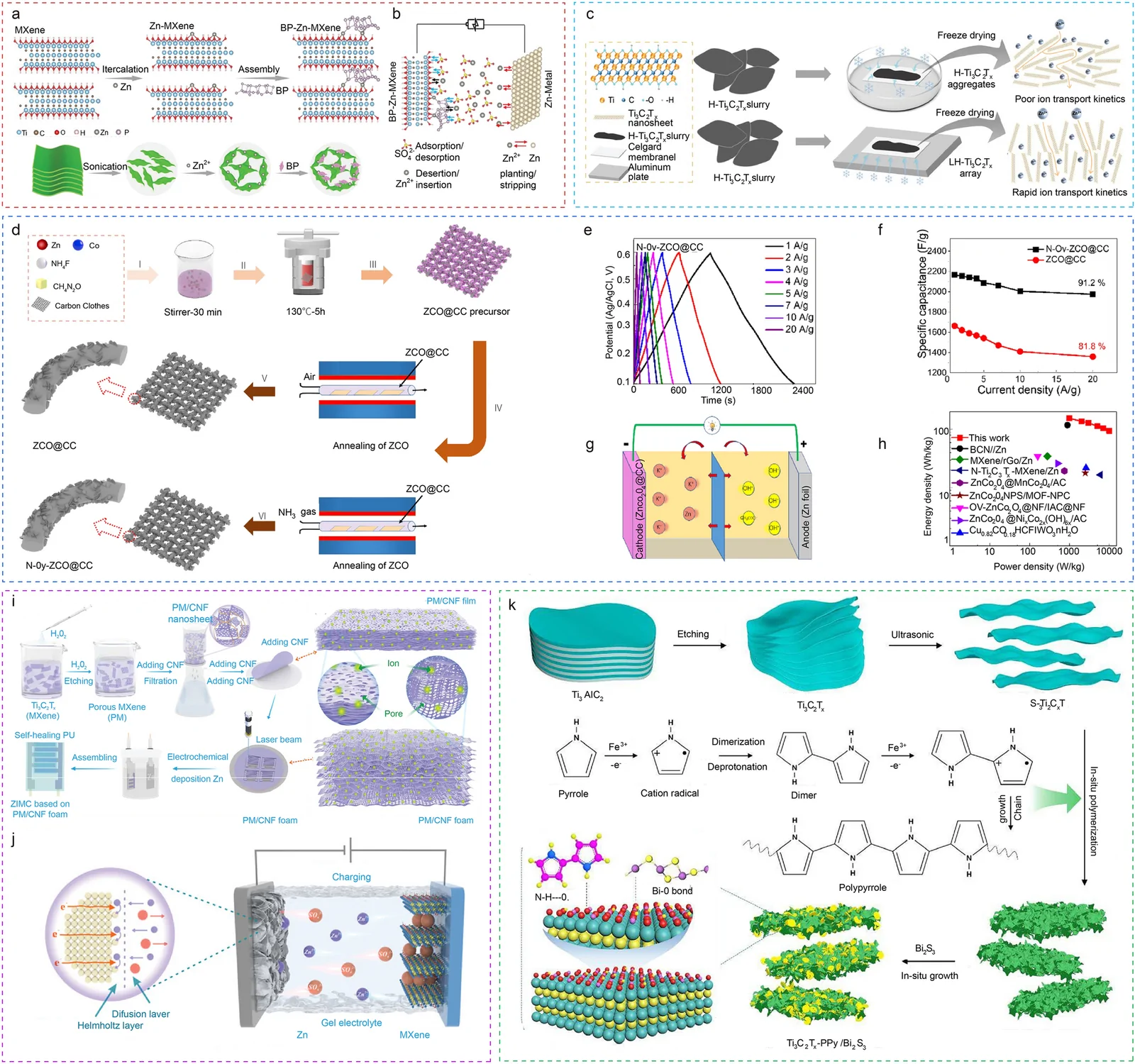
**a** Schematic illustration of synthetic process of BP-Zn-MXene nanocomposites. **b** Schematic illustration of Zn-ion storage mechanism of BP-Zn-MXene electrode [94]. **c** Schematic illustration of the preparation processes for the LH-Ti_3_C_2_T_X_ arrays and H-Ti_3_C_2_T_X_ aggregates [96]. **d** Schematic diagram for hydrothermal preparation of ZCO@CC and N-0y-ZCO@CC nanosheets. **e** GCD curves at various current densities range for N-0y-ZCO@CC. **f** Specific capacitance as a function of current density for N-0y-ZCO@CC and ZCO@CC. **g** Schematic diagram of the assembled N-0y-ZCO@CC//KOH//Zn Device. **h** A Ragone plot of the N-0y-ZCO@CC//Zn-ZIC based on the active mass on both electrodes along with a comparison with recently reported work [97]. **i** Schematic diagram of the preparation of polyimide/carbon nanofiber (PM/CNF) foam, the assembly of ZIC based on this foam, and the electrode structures of its film and foam (with corresponding local structure magnification). **j** Schematic diagram of ion transport in the ZIC during charging process [101]. **k** Schematic diagram of the formation process of high-voltage window composite Ti_3_C_2_T_X_-PPy/Bi_2_S_3_ [102]

In view of this, researchers often use MXene to achieve the improvement effect. For example, as shown in Fig. 8a, Fang et al. significantly improved the performance of ZICs assembled with MXene-based cathode materials by a two-step molecular engineering strategy of pre-inserting Zn^2+^ and black phosphorus nanosheets [94]. This method accommodates BP nanosheets by expanding the interlayer spacing of MXene as a scaffold. This not only provides sufficient accessible active sites, but also creates an efficient ion migration pathway, thereby significantly enhancing the storage capacity and electronic conductivity of Zn^2+^. The experimental results show that the BP-Zn-MXene hybrid exhibits an area capacitance of up to 2.11 F cm^−2^ and an antiself-discharge ability of 46.5 mV h^−1^. In addition, the wearable ZICs based on this hybrid have an ultra-high CE of 101.1% and a high areal energy of 426.3 μWh cm^−2^ (Fig. 8b). In addition, researchers have used a variety of high-conductivity materials to combine with metal-based compounds to significantly enhance their electron transport capacity and electrochemical performance. For instance, Zheng et al. prepared VS_4_/RGO composites (3D-VG) with high crystallinity by GO regulation and applied them to ZICs [95]. The team uniformly anchored VS_4_ nanosheets on a three-dimensional hierarchical RGO framework by a simple hydrothermal method. RGO not only promotes the uniform growth of VS_4_ as a space confinement agent, but also acts as an oxidant to reduce sulfur vacancies to optimize the diffusion kinetics of Zn^2+^. This structural design significantly enhances the conductivity and ion diffusion kinetics of VS_4_, and optimizes the insertion/extraction process of Zn^2+^. The experimental results show that the ZICs based on 3D-VG exhibit excellent rate performance and cycling stability. The device exhibits a maximum energy density reaching 296 Wh kg^−1^, a power density of 400 W kg^−1^, and an 86.7% capacity retention rate even after undergoing 10,000 cycles.

It is noteworthy that, in addition to the above hybrid strategy, regulating the intrinsic micro environment of MXene through structural engineering is also a key means to optimize its conductive network and promote efficient charge transport. Figure 8c, the vertically aligned MXene array (LH-Ti_3_C_2_T_X_) with microchannel structure, prepared by Lin et al. via the  ice-templating method and hydrochloric acid treatment, significantly improved the capacitor performance [96]. This microchannel structure directly shortens the ion transport path and improves ion transport efficiency, thereby indirectly enhancing electronic conductivity. Meanwhile, the introduction of hydrochloric acid during the preparation process causes the surface of MXene nanosheets to form a wrinkled morphology, increasing the number of active sites. These active sites can not only promote ion adsorption and desorption, but also provide more transport paths for electrons to improve electronic conductivity. The incorporation of the LH-Ti_3_C_2_T_X_ cathode enables ZICs to exhibit excellent rate performance and long-term cycling stability under high-current densities, being able to cycle stably for more than 50,000 cycles at a current density of 5 A g^−1^. At the level of regulating the intrinsic chemical properties of materials, doping and defect engineering also show strong advantages (Fig. 8d) [97]. For example, in the ZnCo_2_O (ZCO) system, nitrogen doping forms Co–O–N bonds by substituting lattice oxygen, which optimizes the electronic structure and reduces the electron transport barrier. Meanwhile, the introduced oxygen vacancies serve as additional active sites, promoting the adsorption and diffusion of Zn^2+^. In addition, the unique mesoporous structure of the material and the highly conductive carbon cloth substrate further accelerate the ion/electron transport kinetics. Figure 8e, f shows that the modified N–O–V-ZCO@CC electrode exhibits an ultra-high specific capacitance of 2166.4 F g^−1^ at 1 A g^−1^, a capacity retention rate of 91.2% at 20 A g^−1^, and 98.99% capacity retention maintained following 5,000 cycles. The ZIC device assembled based on this positive electrode achieves an energy density of 95.35 Wh kg^−1^ (with a power density of 10,008 W kg^−1^), which outperforms the majority of comparable systems (Fig. 8 g, h). This work indicates that doping and defect engineering of TMCs can synergistically improve their intrinsic conductivity and reaction activity, providing new ideas for the design of high-energy density zinc-ion energy storage devices.

Although the aforementioned strategies have effectively alleviated the conductivity bottleneck of TMCs, they still face the critical challenge of structural collapse under actual operating conditions. For instance, the 2D layered structure of MXene is bound by weak van der Waals forces, making it prone to interlayer sliding or peeling during mechanical stress or cyclic charge–discharge processes [98]. Its surface usually has terminal groups such as –OH, –O, or -F, which are prone to oxidation or hydrolysis reactions in high humidity, high temperature, or electrochemical environments, thus damaging the surface structure [99]. Fortunately, in response to the above issues, current researchers are actively adopting strategies such as surface modification, composite materials, and construction of porous structures to significantly enhance the stability of MXene. Firstly, breakthroughs have been made in research based on surface engineering strategies: KOH solution is used for alkali treatment of Ti_3_C_2_T_X_, by converting surface -F terminal groups into -OH and -O groups [100]. This not only significantly enhances the hydrophilicity of the material but also effectively weakens the interlayer van der Waals forces. The self-supporting O-Ti_3_C_2_ MXene film prepared by combining vacuum filtration and exfoliation technology has a unique three-dimensional interconnected microstructure that provides sufficient channels for electrolyte penetration. It still maintains a capacity retention rate of 96.7% after 10,000 cycles, demonstrating excellent deformation resistance.

To further improve the interface stability, researchers have developed composite material design strategies. Within the realm of flexible devices, the interlayer insertion strategy shows unique advantages. For instance, the MXene/VS_4_ composite electrode constructed by researchers exhibits excellent performance as the cathode material for ZICs [103]. VS_4_ suppresses stacking by expanding the interlayer spacing of MXene, which is confirmed by the shift of the (002) peak. The flexible device operates stably in a wide temperature range (0–75 °C), with a capacitance retention of 91.9% after 2,000 bending cycles. This performance benefits from the structural toughness of VS_4_-anchored MXene layers and the interface adaptability of PVA gel electrolyte. Such mechanical toughness stems from the synergism between the anchoring effect of VS_4_ and the PVA gel electrolyte, providing a reliable solution for wearable devices. In response to the demand for large-scale production, significant breakthroughs have been made in low-cost synthesis strategies [104]. After being compounded with nanocellulose, the formed three-dimensional network structure disperses interlayer stress through hydrogen bonding, preventing interlayer stacking or collapse. As a result, the composite film simultaneously possesses a tensile strength of 59.7 MPa and an electrical conductivity of 24,754 S m^−1^.

The optimization of the material's microstructure is equally crucial. Han et al. found that after α-ZnPc was converted to β-ZnPc through annealing treatment, the material exhibited a higher SSA and a more abundant porous structure, thereby bringing about a significant enhancement in its electrochemical behavior. The abundant porous structure not only boosts the utilization efficiency of active sites, but also provides space to accommodate the volume expansion caused by Zn^2+^ insertion, thus alleviating the problem of easy collapse of the material [105]. Meanwhile, Wang’s team skillfully combined physical intercalation, chemical pore-forming, and gas-foaming technologies to successfully construct a MXene electrode with a hierarchically interconnected porous structure spanning micro-, meso-, and macropores (Fig. 8i) [101]. This innovative design has greatly enriched the multi-level pore structure of MXene, effectively solving the problem of material collapse. In addition, its unique pore structure exposes more active sites, thereby significantly enhancing the electrochemical performance. When this porous MXene material is used as the cathode, the constructed ZICs exhibit excellent performance (Fig. 8j). Specifically, its areal specific capacitance reaches as high as 410 mF cm^−2^, and the energy density also reaches 103 µWh cm^−2^. These research cases indicate that through reasonable material design and structural optimization, the problem of material collapse in ZICs can be effectively inhibited, and the cycling stability and electrochemical performance of the devices can be significantly improved. These strategies point out the direction for the development of next-generation high-stability ZICs.

Nevertheless, beyond structural stability, the sluggish reaction kinetics of TMCs remain a critical bottleneck restricting their performance improvement. This issue stems from the fact that their pseudocapacitive energy storage mechanism primarily relies on redox reactions on or near the surface, and the rate of these reactions is generally slower than the physical adsorption process of EDLC. To address this challenge, researchers have further explored strategies to accelerate reaction kinetics through interface regulation and spatial structure design. Firstly, through constructing a 3D porous conductive framework formed by coating MXene sheets with a PPy “ear-like” network and combining it with regularly grown Bi_2_S_3_ nanosheets, researchers have not only inhibited volume expansion but also established open ion transport channels (Fig. 8k) [102]. This design achieves a cycling capacity retention rate of 75.44%. Theoretical calculations indicate that the electronic orbital hybridization between Bi_2_S_3_ and MXene forms a metallic composite, which enhances charge separation and transport capabilities by reducing the band gap. The experimentally measured low impedance slope further verifies the optimization of ion migration paths under the synergism between the 3D structure and nanomaterials, providing a dual solution of structural design and interface regulation to break through the kinetic bottleneck of electrode materials.

Furthermore, another study proposes a cathode material, RuO_2_ QDs@PCNCs, which anchors RuO_2_ quantum dots onto porous carbon nanocages (PCNCs) for ZICs [106]. This structure can effectively inhibit the agglomeration of RuO_2_ quantum dots, provide a large number of electrode–electrolyte interfaces and abundant active sites, thereby promoting ion storage and rapid electrochemical kinetics. The porous structure of PCNCs not only shortens the ion transport path but also increases the number of electrochemically active sites, which enhances the material’s conductivity and ion diffusion rate, significantly improving the reaction kinetics of the TMCs RuO_2_. Experimental results show that the RuO_2_ QDs@PCNCs cathode material exhibits a large capacity (224 mAh g^−1^), excellent rate performance, and good cycling stability within the voltage range of 0.4–1.8 V. Besides, researchers have also successfully developed a high-performance ZICs (BCNP//rGO-Zn) by constructing multi-channel basic cobalt–nickel phosphate core–shell microspheres (BCNP) and combining them with an rGO-Zn anode [107]. Through an in situ ion exchange process, the exchange of macromolecular BTC^3−^ with small molecular OH⁻ and PO_4_^3−^ not only inherits the SSA of the MOF precursor but also generates an abundant “tree-vein” like multi-channel structure. This structure significantly enhances the diffusion rate of Zn^2+^ in the electrolyte and the efficiency of ion migration. Meanwhile, the formation of amorphous cobalt–nickel phosphate provides more active centers, facilitating the progress of electrochemical reactions and thus addressing the issue of sluggish reaction kinetics in TMCs. The BCNP//rGO-Zn device achieves a high-energy density of 376.5 Wh kg^−1^ at a power density of 1.66 kW kg^−1^ and exhibits a stable output voltage.

In summary, significant progress has been made in optimizing the performance of TMCs as cathode materials for ZICs through multi-dimensional strategies. Researchers have achieved breakthroughs in enhancing conductivity, inhibiting structural collapse, and accelerating reaction kinetics via strategies such as hybridization, structural engineering, and doping regulation. However, challenges related to cyclic stability and large-scale fabrication remain for practical applications. Future research needs to further explore the synergistic optimization mechanism between the intrinsic properties of materials and device operating conditions, and achieve a dynamic balance between charge transport and structural stability through multi-scale structural design. Furthermore, developing green and low-cost large-scale preparation processes and constructing a full-chain optimization system encompassing materials, electrodes, and devices will be the key paths to facilitate the practical utilization of zinc-based energy storage devices.

### Battery-Type Anode Zn

As the core anode of ZC-ZICs, the zinc electrode faces multiple challenges in practical applications: Uncontrollable dendrite growth causes the risk of short circuits, HER results in a reduction of CE, and excessive zinc usage results in kinetic imbalance. Despite its high theoretical capacity and redox potential close to that of metallic lithium, these inherent drawbacks severely restrict the long-cycle performance of the devices. Therefore, this paper classifies the problems faced by zinc anodes into two categories and summarizes various corresponding strategies. Some examples of strategies to solve the zinc anode problem are summarized in Table 3.

**Table 3:** Comparative evaluation of the challenges, stabilization strategies, and electrochemical performance of zinc anodes in ZICs

Problem	Strategy	Anode	Cathode	Electrolyte	*V*_w_	*E*_d_	*P*_d_	*C*_R_ (*C*_n,_ *C*_d_)	References
Inhibition of zinc dendrite growth and occurrence of side reactions	Interfacial layer regulation	Zn	AC	BBI + ZnSO_4_	0–1.8	80 Wh kg^−1^	9 kW kg^−1^	82.4% (300,000, 10 A g^−1^)	[108]
		ZnPTA Zn	AC	ZnSO_4_	0.2–1.8	NA	NA	77.9% (5,500, 2 A g^−1^)	[109]
		Zn	Carbon materials	B_6_ + ZnSO_4_	0.2–1.7	88.2 Wh kg^−1^	26.92 kW kg^−1^	NA (200,000, 5 A g^−1^)	[110]
		PBA Zn	AC	ZnSO_4_	0–1.8	33.7 Wh kg^−1^	1.6 kW kg^−1^	92.41% (10,000, 1 A g^−1^)	[111]
		Zn ZnAl	AC	ZnSO_4_	NA	NA	NA	90% (10,000, NA)	[112]
		Zn ZIF-8-110	AC	ZnSO_4_	NA	NA	NA	NA (3,500, 0.1 A g^−1^)	[113]
	Electrolyte additive	Zn	AC	ZnSO_4_ + CMC-Na	0.2–1.8	70.7 Wh kg^−1^	4.70 kW kg^−1^	96% (30,000, 5 A g^−1^)	[114]
		Zn	AC	ZnSO_4_ + CMC	NA	NA	NA	100% (18,000, 5 A g^−1^)	[115]
Inhibition of zinc dendrite growth and occurrence of side reactions	Electrolyte additive	Zn	AC	ZnSO_4_ + MTC	0.2–1.8	NA	NA	94.6% (28,000, 5 A g^−1^)	[116]
		Zn	YEC-8	ZnSO_4_ + TPAB	0–1.8	NA	NA	NA (10,000, 5 A g^−1^)	[117]
		Zn	NPHC	DT + ZnSO_4_	0.2–1.8	NA	NA	90% (20,000, 5 A g^−1^)	[118]
	Cathode material modification	Zn	BM-NSC-2	Zn(CF_3_SO_3_)_2_/ DMF	0.2–1.8	94 Wh kg^−1^	33.53 kW kg^−1^	100% (10,000, 5 A g^−1^)	[119]
	Crystal plane regulation	MG-Zn(002)	AC	Zn(OTf)₂	NA	84.4 Wh kg^−1^	8.5 kW kg^−1^	88% (10,000, 10 A g^−1^)	[120]
	Hydrogel confinement	Zn	AC	COOH-f-CellPZ_2.0_-gel	0.2–2.0	101 Wh kg^−1^	16 kW kg^−1^	91% (70,000, 5 A g^−1^)	[123]
		Zn	AC	ZnSO_4_ + PAB	NA	NA	NA	NA (7,500, NA)	[125]
		Zn	C	SZHE	NA	169.7 Wh kg^−1^	NA	96.5% (50,000, 5 A g^−1^)	[126]
	Functional separator design	Zn	AC	ZnSO_4_	NA	NA	NA	NA (5,000, 1 A g^−1^)	[127]
		Zn	AC	ZnSO_4_	0–1.6	NA	NA	NA (10,000, 1 A g^−1^)	[128]
Zinc excess	Enhancing cathode capacity	Zn	LAC	Zn(CF_3_SO_3_)_2_/ZnBr_2_/PEG	0–2.2	353.1 Wh kg^−1^	NA	94.8% (5,000, 5 A g^−1^)	[133]
		Zn	YP-50F	Zn(OTf)_2_ + Cu(OTf)_2_ in acetonitrile	0–2.0	41 Wh kg^−1^	3.5 kW kg^−1^	NA (22,000, 25 mA cm^−2^)	[134]
	Improving zinc utilization efficiency	Zn	GPCN-2	CEEZ	0–1.8	124.88 μWh cm^−2^	8.1 mW cm^−2^	97.2% (10,000, 20 mA cm^−2^)	[124]
		Zn PCCF	PCCF	LiCl + ZnCl_2_	NA	2.02 mWh cm^−2^	11.47 mW cm^−2^	NA (30,000, 20 mA cm^−2^)	[135]
		Zn on Cu foil	Cl_2_-activated AC	ZnCl_2_	0–2.0	192 ± 0.56 Wh kg^−1^	NA	84% ~ 92% (50,000, 25 mA cm^−2^)	[132]

*V*_w_, voltage window (V); *E*_d_, energy density; *P*_d_, power density; *C*_R_, capacity retention (%); *C*_n_, cycle number; *C*_d_, current density; NA, not available

#### Inhibition of Zinc Dendrite Growth and Occurrence of Side Reactions

1. Interfacial layer Regulation Strategy

Interfacial layer regulation is a key strategy to optimize the electrode–electrolyte interface behavior by establishing a functional protective layer on the zinc foil surface. Its core lies in synergistically addressing issues including dendritic growth, HER, and corrosion through means like physical isolation, chemical passivation, and ion transport guidance.

**Fig. 9:**
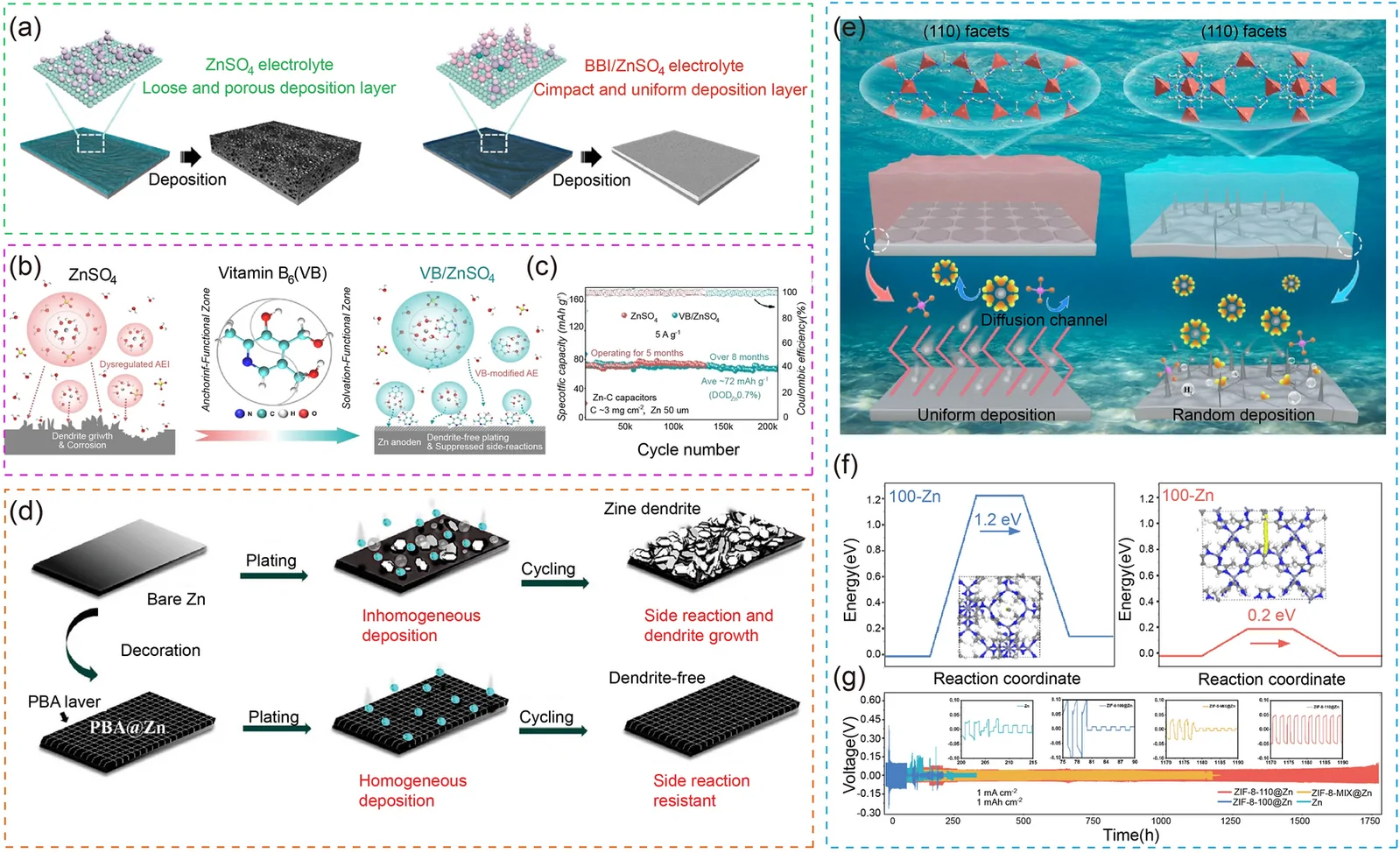
**a** Schematic illustration of Zn deposition behaviors in two electrolytes [108]. **b** Schematic illustration showing the role of VB-modified functional anode–electrolyte interface in regulating Zn deposition and side reactions. **c** Long-term cycling performance of Zn−C capacitors at 5.0 A g^−1^ [110]. **d** Schematic of the structural cycle of Zn and PBA@Zn coatings [111]. **e** Comparison of reactions between the (110) crystal surface and (100) crystal surface in ZIF-8. **f** Migration energy barriers of Zn^2+^ at the (100) and (110) crystalline surfaces. **g** Long-term cycling performance of Zn//Zn symmetric batteries with the anode of bare Zn and ZIF-8-X@Zn at 1 mA cm^−2^ for 1 mAh cm^−2^ [113]

Among them, interface engineering schemes represented by the diphenyl sulfonylimide (BBI) additive have shown significant advantages [108]. The BBI additive forms an interfacial layer on zinc metal through chemical adsorption. Its regulatory mechanism is as follows: BBI anions undergo strong chemical adsorption with zinc atoms, replacing the original polarized water molecule layer and converting the original water-based EDL into a water-poor EDL (Fig. 9a). This transformation introduces an additional Zn^2+^ migration barrier, increasing the desolvation activation energy of Zn^2+^ and thereby significantly enhancing the zinc nucleation overpotential. The increased nucleation overpotential promotes uniform nucleation of Zn^2+^ on the electrode surface instead of local aggregated growth, forming a dense and uniform zinc deposition layer, which effectively avoids the formation of dendrite structures. Eventually, during the cycling process, the BBI-Zn interfacial layer evolves into an SEI. Through the dual effects of physical barrier and chemical passivation, the SEI further maintains interface stability and inhibits dendrite recurrence in long-term cycling. Researchers further observed the microstructure of the zinc deposition layer via SEM and found that the zinc coating exhibits a denser and flatter crystal structure after adding BBI. This observation, in conjunction with XRD results, mutually corroborates and jointly reveals the facet-selective regulatory effect of the BBI additive on zinc deposition behavior. This strategy, leveraging the synergy of interface barrier design, deposition behavior regulation, and SEI layer, creates a unique dendrite inhibition and separator protection mechanism for ZICs, boosting device safety and cycle life (assembled ZICs boast over 300,000 cycles of ultra-long life).

Furthermore, Liu et al. pioneered the use of zinc terephthalate composite (ZnPTA) as the interfacial layer, a strategy that has significantly enhanced the stability and electrochemical performance of zinc anodes [109]. Owing to its unique structure, the ZnPTA interfacial layer can precisely regulate the flux of Zn^2+^ and the electric field intensity, enabling Zn^2+^ to exhibit a high degree of uniformity during the deposition process. This characteristic fundamentally inhibits the uncontrolled growth of zinc dendrites. Moreover, the ZnPTA layer also significantly suppresses the occurrence of side reactions. Its dense structure provides uniform nucleation sites for Zn^2+^, avoiding the disordered deposition of Zn^2+^ in the electrolyte and side reactions with other components, thereby reducing the possibility of parasitic reactions and enhancing the resistance to corrosion and cycling stability of the zinc anode. This innovative design not only prolongs the cycle life of ZICs and improves their capacity retention rate but also establishes a fundamental basis for the advancement and industrial application of zinc-based electrochemical energy storage devices.

Additionally, recent studies have made significant progress in introducing multi-functional biomolecular additives to regulate the anode–electrolyte interface. Figure 9b shows that Xu et al. realized the efficient stabilization of zinc anode by adding a trace amount of vitamin B_6_ (VB) to the traditional ZnSO_4_ electrolyte [110]. The pyridine nitrogen in VB molecules, acting as an “adsorption domain” preferentially anchors to the zinc surface, forms a dense interfacial layer that blocks the direct contact between water molecules and zinc, thereby inhibiting corrosion reactions. Meanwhile, the hydroxyl groups, serving as a “solvation domain,” regulate the coordination environment of Zn^2+^ at the interface, promoting the formation of [Zn(H_2_O)_5_VB]^2+^, reducing the activity of H_2_O molecules, and inducing uniform deposition of Zn along the (002) crystal plane. This strategy significantly enhances the cycling stability of the zinc anode. The ZICs assembled based on this strategy exhibit excellent cycling performance: devices with low-loading cathodes cycle 200,000 times at 5.0 A g^−1^ (Fig. 9c), and those with high-loading cathodes operate stably for 2000 h at an 11.6% depth of discharge. Through the synergistic effect of molecular functional groups, this work provides an innovative approach for designing high-stability zinc anodes and long-life ZICs.

Complementary to the approach of constructing an interfacial layer, constructing a chemically protective interfacial layer directly on zinc foil further blocks zinc-electrolyte side reactions through the synergistic effects of interface passivation and mechanical reinforcement, achieving a more comprehensive improvement in interface stability. Prussian blue analogs (PBA), as a typical 3D open framework material, were used by Tong’s team as an artificial interphase on zinc foil. Through the dual functions of interface regulation and mechanical support, they effectively inhibit the growth of zinc dendrites and the occurrence of side reactions (Fig. 9d) [111]. The 3D porous structure of the PBA coating significantly increases the SSA of the zinc anode, providing more nucleation sites for zinc ions. This homogenizes the flux of Zn^2+^, reduces local electric field distortion and stress concentration, thereby accomplishing the objective of suppressing the non-uniform growth of zinc dendrites. Meanwhile, the PBA coating offers ion transport channels with low-energy barriers, significantly reducing the migration resistance of zinc ions. Its activation energy is only 19.41 kJ mol^−1^, much lower than 23.49 kJ mol^−1^ of pure zinc foil, enabling Zn^2+^ to migrate rapidly and deposit uniformly. Furthermore, the 3D framework structure of the PBA coating restricts the lateral expansion of dendrites through mechanical support. Even under high-current densities, it can effectively inhibit the penetration behavior of dendrites and protect the separator from damage. In the charge–discharge cycles of ZICs, side reactions and parasitic reactions will result in a reduction in CE and deterioration of cycle stability. However, the PBA coating, relying on its large ion channels and polar groups (−CN), reduces the activation energy of Zn^2+^ and improves ionic conductivity, thus suppressing the occurrence of side reactions. Through these mechanisms, ZICs stably cycle 10,000 times at 10 A g^−1^, retaining 118.3% cycle stability with ultra-long-cycle stability.

Similarly, Li’s team proposed an antianion depletion mechanism based on the space charge theory, which involves immobilizing anions using layered double hydroxide (LDH) materials to build an antianion depletion layer on the zinc anode surface [112]. Specifically, researchers selected ZnAl-LDH as the optimal material and successfully fabricated a ZnAl-LDH coating on the zinc foil via an in situ growth strategy. During the zinc deposition process, anions near the zinc anode are continuously consumed, forming an anion depletion zone. This zone induces a strong space electric field, which interferes with Zn^2+^ deposition and thereby triggers the growth of zinc dendrites. The antianion depletion layer, however, can effectively anchor anions, eliminate or weaken the space electric field, and stabilize the deposition behavior of Zn^2+^. Meanwhile, through in situ growth engineering, the microstructure of LDH nanosheets is manipulated to arrange vertically on the surface of the zinc anode, forming rapid and uniform Zn^2+^ ion channels. This regulates the deposition flux of Zn^2+^ and further inhibits the growth of zinc dendrites. Experiments have shown that the Zn@ZnAl anode constructed using this strategy can stably cycle 5,500 times even at a high-current density of 40 mA cm^−2^, demonstrating extremely high cycle stability. By inhibiting the growth of zinc dendrites, the morphological changes of the zinc anode during charge–discharge processes are significantly reduced, thereby lowering the risk of battery short circuits caused by dendrites piercing the separator. This provides dual guarantees for the long-cycling life and safety of ZICs.

While the above strategies offer important guarantees for zinc anode stability, recent research by Xiong’s team has taken a different approach. Researchers coated zinc foil with ZIF-8 materials dominated by different crystal planes (ZIF-8–110 and ZIF-8–100) to form protective layers (ZIF-8–110@Zn), aiming to regulate Zn^2+^ deposition behavior and inhibit dendrite growth (Fig. 9e) [113]. A comparison between ZIF-8–110 and ZIF-8–100 revealed through experimental studies that the ZIF-8–110 coating, dominated by the (110) crystal plane, can form a unique Z-shaped channel structure, which significantly reduces the Zn^2+^ migration barrier from 1.2 to 0.2 eV. This effectively promotes the homogeneous plating of Zn^2+^ (Fig. 9f). The primary reasons are as follows: First, the Z-shaped channels provide exclusive ion migration paths, reducing local concentration gradients; second, the large pore size (approximately 0.8 nm) of the (110) crystal plane prevents the disordered aggregation of Zn^2+^; finally, the high positive charge on the coating surface homogenizes the Zn^2+^ flux through electrostatic repulsion, inhibiting the preferential growth of dendrite tips. Experiments showed that the ZIF-8–110@Zn device achieves a cycle life of 1750 h at 1 mA cm^−2^, which is about 9 times that of untreated zinc foil (Fig. 9g). Moreover, in situ optical microscopy observations indicated that its deposition layer is flat and dense, with no obvious dendrite formation. This chemical protective coating complements the ion transport optimization of the solid electrolyte layer, and through the synergistic effect of interface passivation and mechanical reinforcement, it provides a new path for achieving more comprehensive zinc dendrite inhibition and interface stability. Although the above strategies differ in approaches, they all share the core goal of improving the cycle stability of zinc anodes, offering important insights for the long-life design of ZICs.

(2) Electrolyte Additive Strategy

**Fig. 10:**
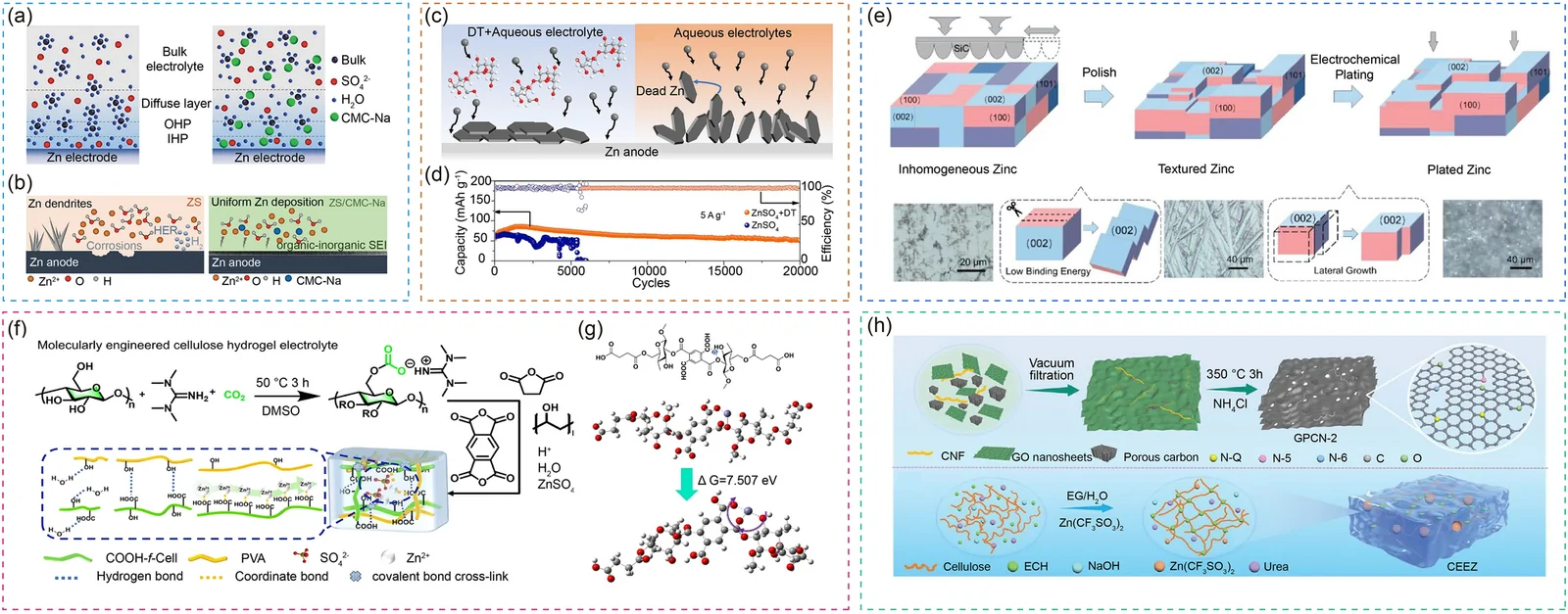
**a** Schematic illustrations of EDL structure in ZS and ZS/CMC-Na. Zn^2+^ density distribution on the Zn anode surface. **b** Schematic illustration of the underlying mechanism of Zn deposition in ZS and ZS/CMC-Na [114]. **c** Schematic diagram of Zn deposition on Zn anode with different electrolytes.** d** Long-cycle performance of Zn//NPHC ZICs with different electrolyte at 5 A g^−1^ [118]. **e** Schematic diagram of constructing (002)-textured zinc anode by MG[120].** f** Illustration of the preparation of COOH-*f*-CellPZ-gel electrolyte. **g** The optimized structure, dissociation energy, and molecular electrostatic potential of -COOH and -COO in COOH-f-Cell bound to Zn^2+^ [123]. **h** Synthetic illustration of GPCNs and CEEZ [124]

Building on the significant improvement in zinc anode stability achieved by the interfacial layer regulation strategy through chemo-mechanical synergism, researchers have further extended the optimization dimension to the intrinsic regulation of electrolytes. By introducing functional additives, they have constructed multi-path mechanisms to inhibit dendrite growth. Sodium carboxymethyl cellulose (CMC-Na), as a multi-functional additive, has continued to attract close attention from researchers in recent years. Recently, Fang et al. employed CMC-Na as an additive to realize a dendrite inhibition mechanism by regulating the EDL structure and in situ forming an organic/inorganic hybrid SEI layer (Fig. 10a) [114]. CMC-Na molecules preferentially adsorb on the surface of the zinc anode, altering the composition of the inner layer of the EDL and reducing the concentration of SO_4_^2−^, thereby lowering the probability of HER and corrosion. Furthermore, molecular dynamics simulations confirm that the introduction of CMC-Na makes the distribution of Zn^2+^ at the interface more uniform, inhibiting local electric field concentration. During cycling, CMC-Na reacts with Zn^2+^ to form a composite SEI layer composed of organic carboxyl groups and inorganic zinc sulfide. This layer possesses both high-ionic conductivity and mechanical strength, which can not only isolate the electrolyte from zinc metal but also promote the uniform deposition of Zn^2+^, avoiding the formation of dendrite tips (Fig. 10b). Experimental verification shows that ZICs using colloidal electrolytes exhibit excellent cycling stability. The capacitance retention rate is still as high as 97% after 15,000 cycles at low-current density. It is worth noting that even at a current density of up to 5 A g^−1^, the capacitance retention rate remains at 96% after 30,000 cycles.

Huang’s research team also introduced CMC-Na as an electrolyte additive, achieving effective inhibition of zinc dendrites [115]. Notably, CMC molecular chains preferentially adsorb on the (101) crystal plane of zinc, reducing the surface energy anisotropy and promoting zinc deposition dominated by the flat (101) crystal plane, thereby avoiding the formation of dendrite tips. Secondly, the coordination between CMC and Zn^2+^ alters the solvation structure, converting [Zn(H_2_O)_6_]^2+^ into [Zn(H_2_O)_4_(COO⁻)]^2+^, which reduces the content of free water and inhibits HER and corrosion reactions. Theoretical calculations further reveal that CMC adsorption decreases the zinc nucleation energy barrier from 64 to 52 mV and reduces the grain size by 1.9 times, inhibiting the kinetics of dendrite growth. Experimental verification shows that the electrolyte with 15 mg mL^−1^ CMC added enables the device to achieve a cycle life of 3700 h at 1 mA cm^−2^ with a capacity retention rate of 98.8%, and in situ optical observation reveals that the zinc deposition layer is dense without dendrites. The strategy of using CMC-Na as an electrolyte additive to inhibit zinc dendrite growth provides a new solution for the long life and high safety of ZICs.

In addition, 1,4,7,10-tetraazacyclododecane (TC), tetrapropylammonium bromide (TPAB), and D-trehalose dihydrate (DT) have all received focused attention and research from researchers. For example, Bu’s team added TC to ZnSO_4_ aqueous solution to optimize the deposition behavior of Zn^2+^ [116]. The TC molecule contains four symmetrically distributed N atoms, and its binding energy with Zn species is higher than that with H_2_O, so it is more inclined to adsorb on the Zn (002) plane. In addition, TC molecules participate in the solvation shell of Zn^2+^ to form a stable Zn(H_2_O)_6-X_(TC)_X_^2+^ structure. On the one hand, this structure reduces the content of free water and lowers the desolvation energy of Zn^2+^, thereby promoting the rapid transport of Zn^2+^. On the other hand, by increasing the initial nucleation overpotential of Zn^2+^ on the electrode surface, it promotes the three-dimensional diffusion mode and avoids dendrite-like protrusions formed due to concentration polarization. Moreover, this crystal plane has the lowest surface energy, so Zn^2+^ is more inclined to grow stably at a low rate along the Zn (002) plane, further inhibiting the formation of dendrites and the occurrence of side reactions.

Ji et al. used TPAB as an electrolyte additive. Its zinc-philic cation (TPA⁺) can specifically adsorb on the inner Helmholtz plane (IHP) of the zinc electrode, reconstructing the EDLC [117]. This strategy triggers multiple effects. TPA⁺ displaces water molecules and SO_4_^2−^ in the IHP, leading to the reconstruction of ion/molecule distribution. This promotes the uniform distribution of Zn^2+^ at the electrode/electrolyte interface, thereby slowing down the electrochemical reaction kinetics and restraining the development of zinc dendrites. Secondly, the electrostatic shielding effect of TPA⁺ regulates the crystallographic energy preference of zinc deposition, inducing the layered growth of zinc and the dominant Zn (002) texture. Finally, some of the interface-adsorbed TPA⁺ is electrochemically reduced to form an inorganic–organic hybrid SEI layer, which further enhances the corrosion resistance and stability of the zinc electrode.

Peng et al. innovatively introduced a green and low-cost electrolyte additive (DT) into the ZnSO_4_ electrolyte. By regulating the solvation sheath structure of Zn^2+^ and reconstructing the hydrogen bond network, they significantly inhibited the formation of zinc dendrites (Fig. 10c) [118]. DT molecules preferentially adsorb on the surface of the zinc anode, forming an electrostatic shielding layer that inhibits the formation of differential electric fields. This can guide the oriented deposition of Zn^2+^ on the (002) crystal plane, resulting in a flat and dense deposition layer. After optimizing the traditional ZnSO_4_ electrolyte by introducing the DT additive, the cycle stability of the zinc anode is significantly improved. Its service life exceeds 650 h, and a CE of 99% is achieved, which is nearly ten times that of the original ZnSO_4_ electrolyte system. Based on this innovative electrolyte system, ZICs constructed with hard carbon materials with an N, P dual-doped structure (NPHC) exhibit excellent comprehensive performance. They deliver a discharge capacity of 123.5 mAh g^−1^ at a current density of 0.1 A g^−1^, and maintain a capacity retention rate of over 90% after 20,000 deep cycles at a high rate of 5 A g^−1^, while maintaining a stable CE close to 100% (Fig. 10d). This breakthrough provides a new technical path for the development of high-performance zinc-based energy storage devices. The addition of organic solvents optimizes the deposition behavior of Zn^2+^, significantly improves the cycling stability and interfacial stability of the zinc anode, and provides strong guarantees for the long life and high safety of ZICs.

(3) Cathode material modification strategy

Building on the achievement of uniform Zn^2+^ deposition through the electrolyte additive strategy, researchers have further extended their optimization perspective to the core reaction interface for energy storage—the modification design of cathode materials. Among various methods, high-energy ball milling has attracted considerable attention as a cathode material modification strategy. Zhang et al. employed high-energy ball milling to introduce oxygen-containing functional groups into cathode carbon, forming C–O–Zn bonds and a ZnF_2_ protective layer, which indirectly inhibit anode dendrites [119].

(4) Crystal Plane Regulation Strategy

Through mechanical polishing, which selectively destroying high-energy crystal planes to significantly increase the exposure ratio of the (002) crystal plane is an innovative strategy proposed by Zhang’s research team in recent years (Fig. 10e) [120]. Due to the dense atomic arrangement and low surface energy of the Zn (002) plane, it exhibits a flat layered structure compared to other high-energy crystal planes, making it difficult for zinc dendrites to form on the Zn (002) plane. DFT calculations show that the (002) crystal plane has the lowest Gibbs free energy change and the lowest atomic desorption energy, which makes Zn^2+^ more inclined to grow horizontally rather than vertically on the (002) crystal plane during the deposition process. After mechanical polishing treatment, the zinc anode with (002) texture shows a higher relative texture coefficient during the zinc deposition-stripping cycle, and the relative texture coefficient remains stable throughout cycling. The (002) textured zinc anode after mechanical polishing treatment exhibits high-power density (8500 W kg^−1^) and long-cycle life (more than 10,000 cycles) in ZICs, with a CE exceeding 99.9%. In the field of zinc dendrite inhibition, Song et al. skillfully achieved the construction of the Zn (002) dominant crystal plane on the zinc foil surface and the in situ formation of an amorphous zinc phosphate protective coating through an innovative one-step annealing process. Meanwhile, this strategy also significantly inhibits parasitic reactions like HER and zinc corrosion, thereby improving the electrochemical stability of the zinc anode [121]. Meanwhile, Zou’s team has taken a different approach, developing a new method for crystal plane regulation based on potentiostatic electrodeposition [122]. By precisely controlling the electrodeposition parameters, they successfully induced the formation of high-index Zn (112) planes. Compared with common low-index crystal planes, the Zn (112) crystal plane exhibits a unique stepped edge structure. These high-energy sites serve as preferential nucleation centers for Zn^2+^ deposition, effectively regulating the fine nucleation and growth mode of zinc, and further expanding the application boundary of crystal plane engineering in zinc anode optimization.

(5) Hydrogel Confinement Strategy

In ZICs, the hydrogel confinement strategy provides an innovative solution for regulating Zn^2+^ deposition behavior and interface stability. For instance, Wang et al. effectively addressed the issue of zinc dendrite growth using the dynamic negative feedback mechanism of a self-limiting polyacrylamide hydrogel electrolyte. This hydrogel is formed preferentially at the tips of zinc dendrites through electrochemically initiated polymerization, creating a physical barrier that restricts Zn^2+^ diffusion and reduces local current density, thereby breaking the traditional “positive feedback” growth pattern [125]. The porous structure of the hydrogel and its abundant acyl groups guide the preferential deposition of Zn^2+^ along the crystal plane through coordination, forming a dense and flat plating layer. XRD analysis shows that the intensity of the (002) crystal plane is 17-foldhigher than that of the (101) plane, which effectively inhibits the three-dimensional growth of dendrite tips. Fu et al. proposed a strategy based on a supramolecular zwitterionic hydrogel electrolyte (SZHE) [126]. SZHE effectively inhibits side reactions on the surface of the zinc anode by constructing a low water activity interface, thereby reducing dendrite formation. Zwitterions, in synergy with ethylene glycol (EG), reshape the solvation structure of Zn^2+^, reduce the participation of water molecules, and guide the uniform distribution and deposition behavior of Zn^2+^. This process avoids the accumulation of local overpotential, thus inhibiting dendrite growth. In addition, the dynamic self-healing ability of SZHE can repair microcracks and interface defects caused by dendrite growth or cycling processes, significantly extending the cycle life of the device and effectively preventing short-circuit caused by separator piercing.

Notably, cellulose-based hydrogel electrolytes play a pivotal role in addressing the key challenge of zinc dendrite growth. Their unique structure and properties open up new paths for improving the performance and safety of ZICs. Chen’s team innovatively adopted a carboxylic acid-functionalized cellulose (COOH-f-Cell) hydrogel electrolyte [123]. This electrolyte introduces high-density carboxylic acid groups onto cellulose chains, forming a three-dimensional interconnected network structure (Fig. 10f). The carboxylic acid groups combine with Zn^2+^ through coordination, breaking the solvation structure of [Zn(H_2_O)_6_] ^2+^ and promoting the oriented deposition of Zn^2+^ along the (002) crystal plane to form a flat and dense plating layer. DFT calculations show that the adsorption energy between carboxylic acid groups and Zn^2+^ reaches 7.507 eV (Fig. 10g), which significantly reduces the local current density and the energy advantage of dendrite tips, inhibiting the “tip effect”. Furthermore, the capacity of ZICs remains 91% after 70,000 excellent life cycles at 5 A g^−1^. This strategy opens up a new path for using cellulose hydrogel electrolytes to regulate the deposition behavior of Zn^2+^ in high-performance zinc-based energy storage systems.

(6) Functional Separator Design

The synergistic design of functional ultra-thin separators is a key solution to effectively address the growth of zinc dendrites and prevent separator piercing. The separator adopted by Li et al. consists of a cellulose nanofiber (CNF) membrane substrate and a MOF-derived C/Cu nanocomposite decorative layer [127]. The uniform nanoporous structure of the CNF membrane substrate, in synergy with its high ion transference number, significantly reduces the concentration polarization of Zn^2+^ and inhibits the concentration of local current density. The C/Cu nanocomposite layer provides abundant low-energy zinc nucleation sites through the C/Cu interface modified with high-density defects and heteroatoms. This synergistic effect induces the preferential deposition of zinc along the (002) crystal plane, forming a dense and flat plating layer. Experiments show that the device composed of this substrate and the zinc anode achieves a cycle life of 2000 h at 1 mA cm^−2^, which is 40 times longer than that of the traditional glass fiber separator. Xu et al. optimized the pore structure, electrolyte wettability, mechanical strength, and intermolecular interactions of the CNFs separator through modification and alkali treatment, thereby significantly improving the performance and cycle stability of ZICs [128]. The prepared CNFs separators possess high mechanical strength, which can effectively curb the growth of zinc dendrites. Even when stress is generated during zinc deposition, the separators can maintain their integrity and prevent dendrites from piercing through. In addition, the interaction between the hydroxyl groups on the surface of CNFs and Zn^2+^ hydrates helps regulate the zinc deposition behavior, promoting dense and uniform zinc deposition, thereby reducing dendrite formation. In terms of preventing separator damage, this strategy also shows significant advantages. The CNFs separators prepared through genetic modification and alkali treatment have high porosity and high electrolyte absorption capacity. The high porosity allows more electrolyte to penetrate into the separators, increasing the ionic conductivity of the separators and reducing the resistance to ion migration. At the same time, the electrolyte absorption capacity ensures that the separators remain moist during long-term cycling, preventing them from drying out and cracking due to insufficient electrolyte. Moreover, the prepared CNFs separators exhibit excellent thermal stability and can maintain their structural integrity even at high temperatures, thus preventing deformation or melting of the separators under high-temperature operating conditions.

In the field of ZICs, strategies for inhibiting zinc dendrite growth and separator piercing have formed a multi-dimensional synergistic optimization technology system. These strategies, through the coupling mechanisms of chemical adsorption, physical confinement, energy barrier regulation, and dynamic repair, collectively construct a precise regulatory network for Zn^2+^ deposition behavior. Future investigations should concentrate on in-depth analysis of the dynamic synergistic mechanisms of multiple strategies and the development of bionic intelligent materials, aiming to break through the application bottlenecks of ZICs in terms of high-energy density and extreme working conditions. This will promote their advancement toward next-generation energy storage systems featuring low-cost, high-safety, and ultra-long service life.

#### Zinc Excess

In the structure of ZICs, due to the generally low utilization efficiency of zinc, a large zinc reservoir is often required to achieve acceptable cycling stability [129]. Meanwhile, the limited capacity of traditional carbon-based cathodes reduces the extent to which Zn^2+^ participates in reversible reactions, leaving a considerable portion of metallic zinc in an electrochemically idle state during cycling. Previous studies have shown that metallic zinc has a high theoretical capacity of 820 mAh g^−1^, whereas the practical capacities of various porous carbon cathodes are usually only 80–318 mAh g^−1^ [130]. However, in the actual device assembly, the mass loading of the zinc anode is often higher than that of the cathode, causing the negative-to-positive capacity ratio (N/P ratio) to deviate significantly from the reasonable range, which manifests as an obvious excessive zinc configuration [131]. Based on the above issues, this section will discuss strategies to alleviate excessive zinc from two complementary perspectives: first, improving zinc utilization efficiency to reduce the required zinc reservoir; second, enhancing cathode capacity to achieve a more rational capacity matching with the zinc anode [132].

From the perspective of enhancing cathode capacity, the high-voltage active aqueous electrolyte proposed by Wei et al. offers an effective approach to mitigating zinc excess and N/P imbalance in ZICs [133]. In this study, by introducing a Br^−^/Br_3_^−^ redox couple into the electrolyte, a reversible pseudocapacitive reaction system was constructed on the surface of the carbon cathode, thereby significantly enhancing the cathode capacity. During the charging process, Br^−^ is oxidized stepwise to form Br_3_^−^, which is strongly adsorbed on the surface of activated carbon. This allows Br_3_⁻ to continuously participate in efficient and reversible Faradaic reactions at the interface. During discharge, Br_3_^−^ is reduced back to Br^−^, enabling cyclic utilization. Compared with traditional carbon materials that rely solely on electric double-layer storage, this redox couple provides an additional reversible charge storage pathway, greatly improving the specific capacity of the cathode. This strategy elevates the capacity of activated carbon to approximately 325 mAh g^−1^—about 2–3 times higher than that of conventional carbons—substantially narrowing the capacity gap between the zinc anode and the carbon cathode. The enhanced cathode capacity directly alleviates the need for oversized zinc loading and enables ZICs to operate under a more rational N/P ratio. Such pseudocapacitance-enhanced cathode design represents a fundamental pathway for addressing the issue of zinc excess.

Similarly, the Zn–Cu dual-ion electrolyte system maintains the use of conventional activated carbon cathodes while improving zinc plating/stripping reversibility through controlled Zn^2+^/Cu^2+^ codeposition [134]. This increases the degree of zinc participation during cycling, allowing stable long-term operation even with reduced zinc loading. In addition, increasing the mass loading of the carbon cathode further raises its total capacity, improving capacity matching with the zinc anode. This system reduces the N/P ratio from the conventional 5–10 range to approximately 3.1, while still achieving over 6,700 stable cycles. These results demonstrate that the combination of enhanced zinc utilization and increased cathode loading can effectively reduce the dependence on excessive zinc. This capacity-matching-based strategy likewise provides a feasible route toward more balanced N/P ratios in ZICs.

From the viewpoint of improving zinc utilization, the N-doped holey graphene–carbon nanofiber composite cathode coupled with a high-ionic-conductivity hydrogel electrolyte enhances Zn^2+^ transport dynamics and interfacial stability, enabling highly reversible zinc deposition/stripping even at low temperatures [124]. Benefiting from uninterrupted Zn^2+^ pathways and suppressed parasitic reactions, the device maintains high CE and significantly increases the amount of zinc that can be reversibly cycled per unit area. By improving zinc participation and cycling efficiency, the system operates stably under more reasonable N/P ratios, thereby alleviating the need for excessive zinc caused by poor zinc utilization.

In another study, a strategy combining porous graphitized carbon fabric (PGCF) electrodes with a Li_2_ZnCl_4_·9H_2_O concentrated chloride electrolyte achieves a substantial improvement in zinc utilization, effectively addressing the prevalent issue of zinc excess in ZICs [135]. This electrolyte enables more than 93% reversible zinc deposition/stripping in Zn//PGCF half-cells and significantly lowers the zinc nucleation overpotential, promoting denser and more stable zinc deposition. The high reversibility translates to greater accessible capacity per unit mass of zinc, reducing the zinc inventory required for capacity balance and increasing the “effective participation” of zinc. Unlike systems that rely on thick zinc foils to maintain cycling stability, this system enables long-term operation (> 1200 h) using much thinner zinc layers and delivers nearly degradation-free performance over 30,000 cycles in full cells. These improvements clearly show that enhanced zinc utilization markedly reduces the need for excessive zinc loading.

Furthermore, engineered control over zinc nucleation orientation to achieve highly oriented Zn(002) deposition markedly improves Zn plating/stripping reversibility and Coulombic efficiency [132]. Higher zinc utilization reduces the amount of zinc required to achieve a given capacity, enabling stable operation with thin zinc layers. In parallel, Cl_2_-activated AC cathodes exhibit increased porosity and higher capacity, further reducing the amount of zinc needed to maintain capacity balance. Through the combined effects of improved zinc utilization and enhanced cathode capacity, this system compresses the N/P ratio far below conventional levels and still retains 84% of its capacity after 50,000 cycles. These results further confirm that improving zinc utilization is a key strategy for reducing zinc excess and achieving rational N/P ratios in ZICs.

In summary, these studies indicate that addressing the issue of zinc excess in ZICs requires simultaneous attention to the design of both electrodes and electrolytes. Enhancing cathode capacity—whether by introducing redox-active species or increasing cathode loading—can directly alleviate the capacity mismatch problem [133, 134]. Meanwhile, strategies to improve zinc utilization (including electrolyte molecular engineering, interface regulation, and crystallographic orientation control) enable thinner zinc layers to participate in reversible cycling more effectively, thereby further compressing the N/P ratio to a more practical and material-efficient range [124, 135]. By integrating these complementary approaches, ZICs can achieve a balanced capacity configuration and extend cycling stability, providing a clear pathway for the development of high-performance, low-cost, and scalable ZICs.

## Bottleneck and Modification Strategy of CB-ZICs

CB-ZICs consist of battery-type materials as the cathode and capacitive materials as the anode. The battery-type electrode materials in CB-ZICs mainly include vanadium-based compounds and manganese-based compounds. Compared with capacitive materials that rely on EDLC, these materials exhibit higher specific capacitance through the reversible intercalation/deintercalation reactions of Zn^2+^ in the transition-metal lattice. However, the intrinsic defects of vanadium-based and manganese-based compounds, including narrow interlayer spacing and structural collapse that easily occurs during cycling, seriously restrict their practical applications. Therefore, the modification of vanadium-based/manganese-based compounds has become an important research direction [136]. It is worth noting that the energy storage mechanism of capacitive cathode materials in CB-ZICs is similar to that in ZC-ZICs, which will not be repeated here.

It is worth adding that although the role of separators in CB-ZICs is significantly weaker than that in ZC-ZICs, their influence cannot be completely ignored. As a key medium for electrolyte distribution and ion transport, separators can mitigate capacity decay caused by transition-metal dissolution and reduce interfacial polarization by improving wettability, maintaining electrolyte uniformity, and providing stable ion channels—thus enhancing cycling stability to a certain extent. While separators are not the core factor determining the electrochemical behavior of CB-ZICs, they still play an auxiliary role in stabilizing the interfacial environment.

On the premise that issues at the electrode material level are identified as the primary contradiction, this study further organizes the key bottlenecks that urgently need to be addressed for vanadium-based and manganese-based cathode materials. Additionally, it summarizes the typical modification strategies and their associated electrochemical performances in Table 4, so as to provide a systematic reference for subsequent research.

**Table 4:** Comparative summary of the challenges, mitigation strategies, and electrochemical performance of vanadium-based and manganese-based oxide materials for ZICs

Classification	Problem	Strategy	Anode	Cathode	Electrolyte	*V*_w_	*E*_d_	*P*_d_	*C*_R _(*C*_n,_ *C*_d_)	References
Vanadium-based oxide	Low conductivity	Recombination of material	AC@CC	CoVO-PANI@CC	ZnSO_4_	0–2.0	NA	NA	81.37% (2,000, 50 mA cm^−2^)	[138]
			AC	NVO	ZnSO_4_	0–2.0	27.2 Wh kg^−1^	NA	100% (5,000, NA)	[139]
	Structural collapse	Build a heterojunction	RGM	RGV	Gelatin + ZnSO_4_	0–1.6	107.2 Wh kg^−1^	2.78 kW kg^−1^	81% (10,000, 0.2 A g^−1^)	[140]
		Selenium doping	AC	NVO Se NBs	KOH	0–1.5	13.43 Wh kg^−1^	2.16 kW kg^−1^	87% (8,000, 3.15 A g^−1^)	[141]
Manganese-based compounds	Structural collapse and performance degradation	In situ pre-inserted Zn^2+^	AC	Zn_X_Mn_1-X_Se	ZnSO_4_ + MnSO_4_	0–1.6	483.3 μWh cm^−2^	12.7 mW cm^−2^	85.3% (5,000, 10 mA cm^−2^)	[146]
		Material doping	AC	ZnMO	Na_2_SO_4_	0–1.0	71.5 Wh kg^−1^	NA	92% (8,000, 8 A g^−1^)	[147]
			Zn foil	V-Mn–O	KOH + Zn(CH_3_COO)_2_	0.3–1.8	150.12 Wh kg^−1^	28.82 kW kg^−1^	95.63% (10,000, 1 A g^−1^)	[148]
Manganese-based compounds	Structural collapse and performance degradation	Composite material	AC	NMS NSSs	KOH	0–1.47	29.3 Wh kg^−1^	2.02 kW kg^−1^	97% (30,000, 15 mA cm^−2^)	[149]
			MXene/ ZnCl_2_	MnO_2_-MWCNTs	ZnSO_4_ + MnSO_4_	NA	90.1 Wh kg^−1^	9.25 kW kg^−1^	86.9% (12,000, 10 A g^−1^)	[150]
		“Amorphous engineering” and “pre-inserted cation stability”	AC	K-MnO_X_	Zn(CF_3_SO_3_)_2_ + Mn(CF_3_SO_3_)_2_	0–1.7	206.7 Wh kg^−1^	16.94 kW kg^−1^	81.8% (30,000, 5 A g^−1^)	[151]

*V*_w_, voltage window (V); *E*_d_, energy density; *P*_d_, power density; *C*_R_, capacity retention (%); *C*_n_, cycle number; *C*_d_, current density; NA, not available

### Vanadium-Based Compounds

Vanadium-based materials have become a research hotspot for cathode materials in CB-ZICs due to their multivalent redox properties, high theoretical capacity, and adjustable layered/tunnel structures. It is worth noting that its energy storage mechanism mainly relies on its own abundant vanadium redox valence states (e.g., V^2+^/V^3+^/V^4+^/V^5+^), and realizes the storage and release of charges through the reversible intercalation/deintercalation process of Zn^2+^ in its crystal structure combined with redox reactions [137]. However, vanadium-based materials face challenges such as insufficient conductivity, structural collapse, and dissolution during cycling, and kinetic sluggishness, which need to be improved through strategies like composite conductive materials, atomic doping, electrolyte optimization, and nanostructure design.

**Fig. 11:**
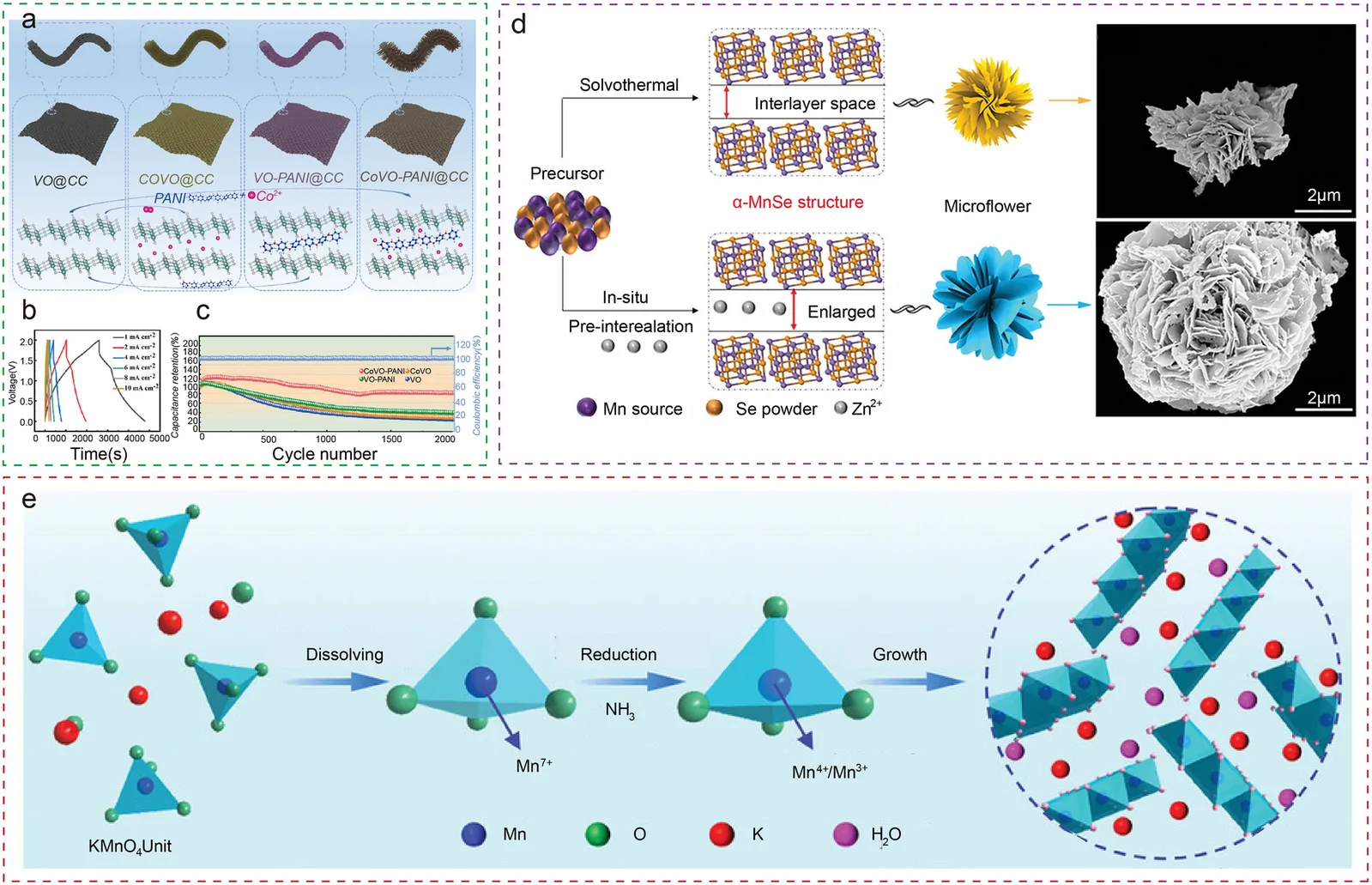
**a** Preparation schematics of VO@CC, CoVO@CC, VO-PANI@CC, and CoVO-PANI@CC are shown. **b** GCD curves of CoVO-PANI at 1–10 mA cm^−2^. **c** Cycle stability tests of CoVO-PANI, VO-PANI, CoVO, and VO at 50 mA cm^−2^ [138]. **d** Function scheme of Zn-ion in situ pre-intercalation in MnSe microflower structure [146]. **e **Schematic formation mechanism of K-MnO_X_ [151]

Among them, V_2_O_5_ has emerged as an ideal electrode material for ZICs owing to its variable valence states, low-cost, and wide-voltage window. Nevertheless, the narrow interlayer spacing and low conductivity of V_2_O_5_ limit its application in ZICs. To address this, Xu's research team reported a ZICs based on Co^2+^ and polyaniline (PANI) doped V_2_O_5_ electrode. They prepared a CoVO-PANI composite electrode by coinserting Co^2+^ and PANI into V_2_O_5_ through a simple hydrothermal method (Fig. 11a) [138]. The introduction of Co^2+^ and PANI significantly expands the interlayer distance of V_2_O_5_, thereby reducing the binding energy for Zn^2+^ insertion and minimizing diffusion barriers. The interlayer spacing of CoVO-PANI reaches 1.41 nm, much larger than the 0.44 nm of pristine V_2_O_5_, which provides greater freedom for the rapid diffusion of Zn^2+^. PANI not only offers additional zinc storage sites but also accelerates the transport of ions and electrons through its conductive network, thus improving the rate performance and cycling stability of the material. The CoVO-PANI with a feather-like nanoarray structure achieves 847.3 mF cm^−2^ at 1 mA cm^−2^, and after 20,000 charge–discharge cycles, it maintains a capacity retention rate of 81.37% and thus demonstrates remarkable rate capability and cycle stability (Fig. 11b, c).

In addition to improving conductivity by introducing conductive polymers, researchers have compounded vanadium-based compounds with various high-conductivity materials, significantly enhancing their electron transport capability and electrochemical performance. Liang et al. studied a new type of layered structure Ni_x_V_2_O_5_⋅nH_2_O (NVO) as the cathode material for ZICs [139]. The NVO material was synthesized via a one-step hydrothermal method, and it was assembled with AC as the cathode into ZICs. The in situ intercalation of H_2_O/Ni^2+^ expands the interlayer spacing of NVO to 13.3 Å, which is larger than that of other reported vanadium-based compounds. This not only enhances ion diffusion capability but also improves the material’s conductivity and cycle stability. In addition, the combination of NVO and AC leverages the rapid EDL ion adsorption/desorption characteristics of AC, further boosting the overall performance of ZICs. Research results show that the nanoflower-like NVO, as the cathode material of ZICs, achieves a specific capacity of 52 mAh g^−1^ at 0.1 A g^−1^, with a power density of 51.6 W kg^−1^ and an energy density of 27.2 Wh kg^−1^. Meanwhile, the ZICs exhibit no obvious capacity decay after 5,000 cycles, demonstrating excellent cycle stability. In ZICs, the intrinsic low conductivity of vanadium-based materials not only limits electron transport efficiency but also causes significant local polarization effects during charge–discharge processes. This polarization phenomenon can lead to uneven stress distribution during Zn^2+^ intercalation/extraction, accelerating the collapse of the layered structure. This issue of structural collapse caused by insufficient conductivity has been addressed by Wang's research team [140]. The researchers developed an aqueous flexible ZICs by constructing a self-supported RGO-V_2_O_5_ (RGV)/RGO-MXene heterojunction. A self-supported RGV cathode was prepared by compositing layered V_2_O_5_ nanorods with RGO. The high conductivity and flexible substrate of RGO were utilized to stabilize the layered structure of V_2_O_5_, inhibit volume expansion during cycling, and achieve a high specific capacity of 323.9 mAh g^−1^ at 0.1 A g^−1^ along with excellent rate performance. The incorporation of RGO not only provides a highly conductive substrate but also stabilizes V_2_O_5_ through its layered structure, preventing its collapse. This design significantly enhances the ion diffusion capability and electrochemical performance of the material. Based on this, the ZICs achieve a specific capacitance of 175 F g^−1^ at 0.5 mV s^−1^ within a wide-voltage window of 1.6 V, with an energy density of 107.2 Wh kg^−1^, a power density of 321.6 W kg^−1^, and a capacity retention rate of 81% after 10,000 cycles. This study provides a new strategy for addressing the issues of conductivity, structural stability, and interface dynamics of vanadium-based materials, and its self-supported flexible design offers important references for the integrated energy storage of wearable electronic devices.

In addition, doping is also an important means to improve electrochemical performance. Shanthappa et al. studied selenium-doped sodium vanadate (Na_2_V_6_O_16_@Se, NVO@Se) nanobelts as electrode materials for aqueous ZICs [141]. After synthesis via a hydrothermal method followed by calcination under a nitrogen flow, the NVO@Se nanobelts exhibit excellent electrochemical performance. Selenium doping not only modifies the crystal structure but also enhances the electrochemical reversibility of the material, thereby boosting its holistic performance. In addition, the nanobelt structure of NVO@Se NBs provides more ion transport channels and shortens the diffusion path of Zn^2+^, thus increasing ion diffusion efficiency. In ZICs, the combination of NVO@Se and AC demonstrates a high-energy density (13.43 Wh kg^−1^), a high-power density (2163.46 W kg^−1^), and good cycle stability (with a capacity retention rate of 87% after 8000 cycles). This optimization strategy not only improves the electrochemical performance of the material but also provides new ideas for the practical application of ZICs. Through selenium doping and structural optimization, NVO@Se nanobelts show broad application prospects in ZICs, offering important references for the development of high-performance energy storage devices.

In summary, vanadium-based materials have exhibited notable potential in the realm of ZICs cathode materials because of their multivalent redox properties and adjustable structures. In recent years, researchers have mainly adopted strategies such as introducing doping, conductive polymers, and composite high-conductive substrates, successfully overcoming the inherent defects of materials like V_2_O_5_ including narrow interlayer spacing, insufficient conductivity, and poor cycle stability. These strategies have not only greatly improved the specific capacity and cycle stability of the materials but also provided a firm foundation for the practical implementation of ZICs. However, key challenges remain in future research, such as accurately controlling doping distribution in large-scale preparation, optimizing interface compatibility, and developing low-cost and environmentally friendly processes. Future work can further explore material design guided by multi-scale theoretical simulations, the development of multi-functional electrolytes, and device integration technologies to promote the commercialization of high-energy-power density ZICs in wearable electronics and grid energy storage.

### Manganese-Based Compounds

In the cathode material system of ZICs, manganese-based compounds have attracted much attention due to their high theoretical capacity, natural abundance, low-cost, and environmental friendliness, and are regarded as highly potential cathode candidates [142]. It is worth noting that there are still differing views in the academic community regarding the energy storage mechanism of manganese-based compounds. Sun et al. were among the first to propose that in a zinc sulfate electrolyte, ε-MnO_2_ adopts an energy storage mechanism involving the cointercalation of H^+^ and Zn^2+^ [143]. However, subsequent studies have revealed a more complex reaction process. Pan et al.’s research based on a Zn(TFSI)_2_ electrolyte showed that during the discharge process of δ-MnO_2_, Zn^2+^ intercalation with pseudocapacitance-like behavior occurs first, followed by the conversion reaction of H^+^ [144]. Zhang et al. further proposed a hybrid energy storage mechanism in which both cointercalation and coconversion of Zn^2+^/H^+^ take place during the discharge of δ-MnO_2_ [145]. Furthermore, their practical application still faces significant challenges: On the one hand, the poor intrinsic conductivity of manganese oxides leads to sluggish charge transport kinetics, limiting rate performance and power density; on the other hand, the crystal structure distortion and dissolution caused by the Jahn–Teller effect during charge–discharge processes easily result in pulverization of active materials and capacity fading, seriously affecting cycle stability.

To address these issues, recent studies have focused on material compounding and structural optimization strategies to further improve the structural stability of electrodes synergistically. These innovative strategies not only effectively balance the capacity advantages and inherent defects of manganese-based materials but also provide critical technical support for the development of high-energy density and long-life ZICs. Among them, manganese selenide (MnSe) has become a research hotspot in the field of supercapacitors and energy storage devices. An article written by Fang et al. reported a Zn_X_Mn_1-X_Se nanosheet microflower structure prepared by in situ pre-intercalation of Zn^2+^ as the cathode material for ZICs, which effectively overcomes the limitations of traditional cathode materials and improves the performance of ZICs (Fig. 11d) [146]. Researchers adjusted the structure by pre-intercalating metal ions, and pre-inserting K^+^, Na^+^, and Zn^2+^ into the MnO_2_ structure improved the structural stability and capacitive performance to a certain extent. However, excessive pre-intercalation of metal ions would destroy the MnO_2_ structure and affect its performance. In the research on MnSe, by means of in situ Zn^2+^ pre-intercalation, the layered porous structure of Zn_X_Mn_1-X_Se was successfully expanded and stabilized by trying different proportions. As interlayer pillars, Zn^2+^ expanded the interlayer spacing, formed more hierarchical microflower structures composed of micropores and mesopores, increased active sites, improved ion transport rate, and reduced volume changes during charge–discharge processes, thereby significantly enhancing electrochemical performance. Experimental results show that the Zn_0.14_Mn_0.86_Se electrode exhibits ultra-high areal capacitance at a specific current density, and the flexible ZICs assembled based on it also show high-energy density, good cycle stability, and excellent rate performance. The Shakeel Abbas team also significantly improved the electrochemical performance of α-MnO_2_ through the Zn^2+^ doping strategy [147]. They found that the insertion of Zn^2+^ can induce the formation of oxygen vacancy defects, thereby increasing the quantity of active sites in the material and significantly improving its electrical conductivity. This structural optimization not only enhances the specific capacity and rate performance of α-MnO_2_ but also enables it to exhibit excellent potential within the field of electrode materials for supercapacitors, making it a highly competitive candidate.

Similarly, vanadium doping, as an effective modification method, has also been proven to significantly improve the performance of MnO_2_. Iqra Ashraf team introduced a type of ZICs based on vanadium-doped manganese oxide aerogels (V-Mn–O), aiming to address the limitations of manganese-based compounds in electrochemical performance [148]. Through vanadium doping, researchers successfully expanded the interlayer spacing of MnO_2_ from 0.69 to 0.73 nm, thereby significantly enhancing the diffusion kinetics of Zn^2+^. In addition, after vanadium doping, the SSA of the material increased from 43 to 119 m^2^ g^−1^, providing more active sites while improving charge transfer reactions and cycle stability. Experimental results show that the V-Mn–O//Zn capacitor achieves an energy density of 150.12 Wh kg^−1^ at a power density of 900.72 W kg^−1^, and can still maintain an energy density of 96.08 Wh kg^−1^ even at an ultra-high-power density of 28,823 W kg^−1^.

In the research on optimizing electrode materials for ZICs, doping strategies have proven to be an effective method, which regulates the electronic structure and physicochemical properties of materials by introducing different elements. However, a single doping strategy may not fully unleash the potential of materials. Therefore, researchers have further adopted composite strategies to construct composite materials, aiming to achieve more significant performance improvements. The composite strategy, by combining the synergistic effects of two or more materials, not only retains their respective advantages but also makes up for the deficiencies of single materials. For instance, the B.N. Vamsi Krishna team prepared metal molybdenum selenide (MSe/Mo_3_Se_4_, where M is Zn, Mn, or Ni) electrode materials through a simple hydrothermal method, and investigated the impacts of different metal ions on the material structure, morphology, and electrochemical performance [149]. The results show that the nickel molybdenum selenide nanosheet spheres (NMS NSSs) electrode material has a unique hierarchical porous and interconnected structure, with a SSA of 16.35 m g^−2^ and a pore size distribution concentrated around 25.59 nm. This porous structure provides more channels for the penetration and diffusion of electrolyte ions, thereby improving the material's ion diffusion capability and electrochemical reaction activity. At a current density of 1 A g^−1^, the specific capacity of NMS NSSs reaches as high as 252 mAh g^−1^, and after 80,000 cycles, the capacity retention rate is 80% with a CE of 99%. The ZICs assembled with NMS NSSs as the cathode and AC as the anode exhibit high-energy density, high-power density, and excellent cycle stability, providing a promising electrode material option for ultra-long-life energy storage applications. Similarly, the composite strategy has also achieved significant results in the research of other energy storage materials. Mao's team reported a degradable ZICs based on a Zn^2+^ pre-intercalated MXene anode (MXene/ZnCl_2_) and a MnO_2_-multi-walled carbon nanotubes (MWCNTs) cathode [150]. For manganese-based cathode materials, this study constructed a composite cathode (MnO_2_-MWCNTs) by introducing highly conductive MWCNTs, which effectively reduces electrode resistance and enhances rate performance. Combined with the microinterdigital electrode structure prepared by laser direct writing technology, the ion diffusion path and mechanical flexibility were further optimized. DMZHSC exhibits a high-energy density of 90.1 Wh kg^−1^, a capacity retention rate of 86.9% after 12,000 cycles, and environmentally friendly characteristics of rapid degradation within 2 h. This work provides an effective solution to address the issues of insufficient conductivity and structural degradation of manganese-based materials, and promotes the advancement of high-energy density, long-cycling, and degradable energy storage devices.

In particularly, Wang et al. further explored the application potential of amorphous materials in zinc-ion energy storage devices. The team studied an amorphous K-buserite microsphere (K-MnO_X_) as the cathode material for aqueous ZICs [151]. The K-MnO_X_ prepared by the ammonia reduction coprecipitation method irreversibly transforms into amorphous Zn-buserite during the first discharge process (Fig. 11e). This transformed material exhibits excellent energy storage performance, including high specific capacitance, rapid Zn^2+^ diffusion, and a large pseudocapacitive contribution. Amorphous K-MnO_X_ has abundant medium/short-range ordered structural units, which provide a large number of active sites, facilitating the adsorption and storage of Zn^2+^. In addition, the disordered nature of the amorphous structure shortens the Zn^2+^ diffusion pathway, thereby significantly improving the diffusion efficiency of Zn^2+^. This structural characteristic enables Zn^2+^ to be rapidly inserted into and extracted from the electrode material, thus enhancing the material's conductivity and electrochemical performance. The transformed Zn-buserite retains its amorphous nature during cycling, avoiding material collapse or performance degradation caused by changes in the crystal structure. This structural stability once again ensures the high performance of the material during long-term cycling. Experimental studies show that K-MnO_X_ exhibits a specific capacitance of 515.0 F g^−1^ and a cycle stability of 92.9% in ZICs. Furthermore, the K-MnO_X_//AC device can achieve an energy density of 206.7 Wh kg^−1^ and a power density of 16.94 kW kg^−1^, demonstrating excellent electrochemical performance.

In summary, by introducing amorphous K-buserite microspheres and utilizing their irreversible transformation characteristics, the problems of poor conductivity and insufficient cycle stability of manganese-based compounds in zinc-ion energy storage devices are effectively solved, providing new ideas for the design of high-performance ZICs. In the field of ZICs, research on manganese-based compounds as cathode materials has made significant progress through innovative strategies such as pre-intercalation, ion doping, composite structure design, and amorphous transformation. These strategies have not only effectively overcome the inherent defects of manganese oxides, such as poor intrinsic conductivity, lattice distortion caused by the Jahn–Teller effect, and dissolution of active materials, but also significantly improved the electrochemical performance of the materials by optimizing interlayer spacing, constructing conductive networks, introducing porous structures, and enhancing pseudocapacitance contributions. Future research needs to further explore low-cost large-scale preparation technologies, new composite systems, and electrolyte–electrode interface optimization to address challenges such as long-cycle decay, material costs, and adaptability to actual working conditions. Through interdisciplinary integration and engineering design, manganese-based cathode materials are expected to promote the evolution of ZICs toward high-energy density, ultra-long lifespan, and environmental friendliness, laying a scientific foundation for next-generation sustainable energy storage technologies.

## Electrolyte

As a core component of ZICs, the physicochemical properties of electrolyte systems directly determine the key performance indicators of the devices, including cycle life, energy/power density, and safety reliability. However, traditional aqueous electrolytes generally face challenges such as electrode dissolution, dendrite growth, limited voltage window, performance degradation at low temperatures, and potential safety hazards. To break through these bottlenecks, researchers have focused on innovative modification strategies for electrolytes, covering multiple dimensions such as component regulation, structural design, and interface optimization. This section will systematically review the latest research progress and design ideas in inhibiting the dissolution of electrode materials, improving electrolyte stability, kinetic hysteresis optimization, voltage window broadening, low-temperature performance improvement, and safety improvement. Therefore, this section classifies the key challenges faced by electrolyte systems into six groups, and systematically summarizes the corresponding modification strategies and performance improvement pathways. Table 5 comprehensively compares the optimization strategies and electrochemical performance of different electrolyte systems.

**Table 5:** Key electrolyte challenges in ZICs, associated optimization strategies, and representative electrochemical performance

Problem	Strategy	Anode	Cathode	Electrolyte	*V*_w_	*E*_d_	*P*_d_	*C*_R _(*C*_n,_ *C*_d_)	References
Dissolution of electrode material	Regulating electrolyte pH	Zn foil	a-WO_3_·0.5H_2_O	ZnSO_4_ (PH = 2.6)	0–1.4	NA	NA	51.4% (1,000, 1.8 mA cm^−2^)	[152]
		Ti_3_C_2_T_x_	AC	ZnSO_4_ + Al_2_(SO_4_)_3_ (PH = 2.0)	0–2.0	37.7 Wh kg^−1^	4.02 kW kg^−1^	93.9% (10,000, 8 A g^−1^)	[153]
Poor electrochemical stability	Adding additives	Zn foil	AC	Zn(ClO_4_)_2_·6H_2_O + H_2_O/D + DMSO	0.2–1.9	NA	NA	100% (30,000, 2 A g^−1^)	[154]
		Zn foil	AC	ZnSO_4_ + VOSO_4_	0.3–1.9	340.2 Wh kg^−1^	16 kW kg^−1^	91.2% (10,000, 10 A g^−1^)	[155]
		Zn foil	AC-paper	B-PVA/NFC/ZnSO_4_	NA	180 μWh cm^−2^	6.22 mW cm^−2^	95.3% (5,000, 2 mA cm^−2^)	[157]
		Zn foil	AC	PZAIWZ-50(Zn(ClO_4_)_2_)	0–1.8	38.2 Wh kg^−1^	0.9 kW kg^−1^	36.4% (2,500, 0.1 A g^−1^)	[158]
Dynamical hysteresis	Design a new electrolyte system	Zn metal	PFC	Zn(OTF)_2_ + NH_4_OTF	NA	147 Wh kg^−1^	61.1 kW kg^−1^	98.59% (400,000, 30 A g^−1^)	[159]
		Zn foil	AC	Zn(Ac)_2_ + Fad	NA	NA	NA	88.9% (1,000,1 A g^−1^)	[160]
Narrow voltage window	Weak solvation effect	Zn foil	AC	Zn(NTf_2_)_2_/AN:TMS	NA	135 Wh kg^−1^	14.25 kW kg^−1^	81.2% (9000, 15 A g^−1^)	[161]
Narrow voltage window	Interface preferential adsorption and ion channel regulation	Zn foil	AC/carbon cloth	AA + DMAPS + SL + CNC	0.2–1.8	NA	NA	91% (10,000, 5 A g^−1^)	[162]
		Zn foil	AC	SBMA/polyacrylamide/ZnSO_4_	NA	110 Wh kg^−1^	5.6 kW kg^−1^	72% (5,000, 1 A g^−1^)	[163]
Poor low-temperature performance	Adding antifreezes	Zn foil	AC	Zn_3_K_4_–HEE/3H _2_O	0–2.2	343.2 Wh kg^−1^	2.10 kW kg^−1^	97.5% (40,000, NA)	[164]
		Zn metal	CNTs	PAAm-CSA/ZnCl_2_-LiCl	NA	104 Wh kg^−1^	39 kW kg^−1^	98.7% (10,000, 0.2 A g^−1^)	[165]
		Zn foil	AC/CC	PMPG-25 GPE	0.2–1.8	250 Wh kg^−1^	NA	93.1% (−20 °C) (5,000, 5 A g^−1^) 96.6% (25 °C) (5,000, 5 A g^−1^) 84.2% (60 °C) (5,000, 5 A g^−1^)	[166]
		Zn foil	AC	Zn(ClO_4_)_2_ + SL	NA	NA	NA	99.7% (55,000, 5 A g^−1^)	[168]
	Construction of double-network polymer structure	Zn foil	AC/CC	SPHZL-4	0–2.0	230 Wh kg^−1^	19.9 kW kg^−1^	86.7% (20 °C) (20,000, 5 A g^−1^) 76.3% (−30 °C) (20,000, 5 A g^−1^)	[167]
Security risk	Using hydrogel electrolyte	Zn foil	AC	PAM/LA/PSBMA	0–1.75	110.1 Wh kg^−1^	0.44 kW kg^−1^	81% (3500, NA)	[169]
	Interface engineering strengthening	Zn	C	Gel-BT electrolyte	NA	151.6 μWh cm^−2^	NA	83.7% (12,000, 5 mA cm^−2^)	[170]
	Using solid electrolyte	Zn GMHT	GMHT	Gelatin/ZnSO_4_	0.2–1.8	167.3 μWh cm^−2^	5988.1 μW cm^−2^	84.6% (5000, NA)	[171]

*V*_w_, voltage window (V); *E*_d_; energy density; *P*_d_, power density; *C*_R_, capacity retention (%); *C*_n_, cycle number; *C*_d_, current density; NA, not available

### Dissolution of Electrode Material

Traditional zinc-ion electrolytes cause dissolution of electrode materials due to their high pH value, thereby affecting the electrochemical performance of devices. However, the low pH optimization strategy proposed in this paper provides an important idea to solve this problem. By adjusting the pH value of the electrolyte, not only can the dissolution of electrode materials be inhibited, but also the adsorption and desolvation capabilities of Zn^2+^ can be enhanced, thereby significantly improving the ion storage capacity and cycle stability.

Zhuang et al. studied a method to significantly improve the electrochromic and ion storage properties by adjusting the structural water content in amorphous hydrated tungsten oxide films and the pH value of Zn^2+^ aqueous electrolytes [152]. It was found that the introduction of structural water can trigger surface-dominated pseudocapacitive behavior, thereby significantly enhancing the electrochemical activity and kinetic performance of the film. Meanwhile, adjusting the pH value of the electrolyte to below 2.6 can ensure the chemical stability of the hydrated tungsten oxide film, avoid ion trapping and structural damage, and thus achieve a cycle stability of more than 13,000 cycles. Based on this optimization strategy, the research team successfully constructed a high-capacity and stable zinc-ion electrochromic energy storage device. In the above research, the strategy of optimizing the performance of zinc-ion devices by adjusting the electrolyte pH has shown significant effects. At the same time, Ping et al. proposed a strategy to optimize the ZnSO_4_ electrolyte by using Al^3+^ as an acid-regulating dynamic pillar to improve the performance of ZICs [153]. Studies have found that the hydrolysis of Al^3+^ can increase the acidity of the electrolyte, thereby triggering proton pseudocapacitance and improving the transport efficiency of Zn^2+^ by dynamically expanding the interlayer spacing of MXene. Experiments show that this strategy significantly enhances the specific capacitance and energy density of MXene while maintaining good cycle stability. Specifically, the optimized electrolyte enables Ti_3_C_2_T_X_ to achieve a specific capacitance as high as 277.9 F g^−1^ and an energy density of 37.7 Wh kg^−1^ in ZnSO_4_ solution with 0.2 M Al^3+^ added, and retains 93.9% of its capacity after 10,000 cycles. In addition, the ZICs constructed using this strategy exhibit an excellent energy density of 37.7 Wh kg^−1^, which is a significant improvement compared to traditional acidic and neutral electrolytes.

### Poor Electrochemical Stability

Aqueous electrolytes exhibit great application potential in the energy storage field due to their advantages such as low-cost, high-safety, environmental friendliness, and high-ionic conductivity. However, conventional aqueous electrolytes suffer from poor electrochemical stability, which results in low operating voltage and energy density, thereby limiting their application in high-performance energy storage devices. In recent years, researchers have explored diverse methods ranging from acidic regulation to ion synergistic effects by introducing additives into aqueous electrolytes, aiming to solve the aforementioned-issues and further improve the electrochemical performance of ZICs. These additives can not only optimize the chemical and physical properties of the electrolyte but also significantly enhance the comprehensive performance of ZICs by regulating ion transport kinetics, inhibiting the dissolution of electrode materials, and improving the electrode/electrolyte interface interaction.

**Fig. 12:**
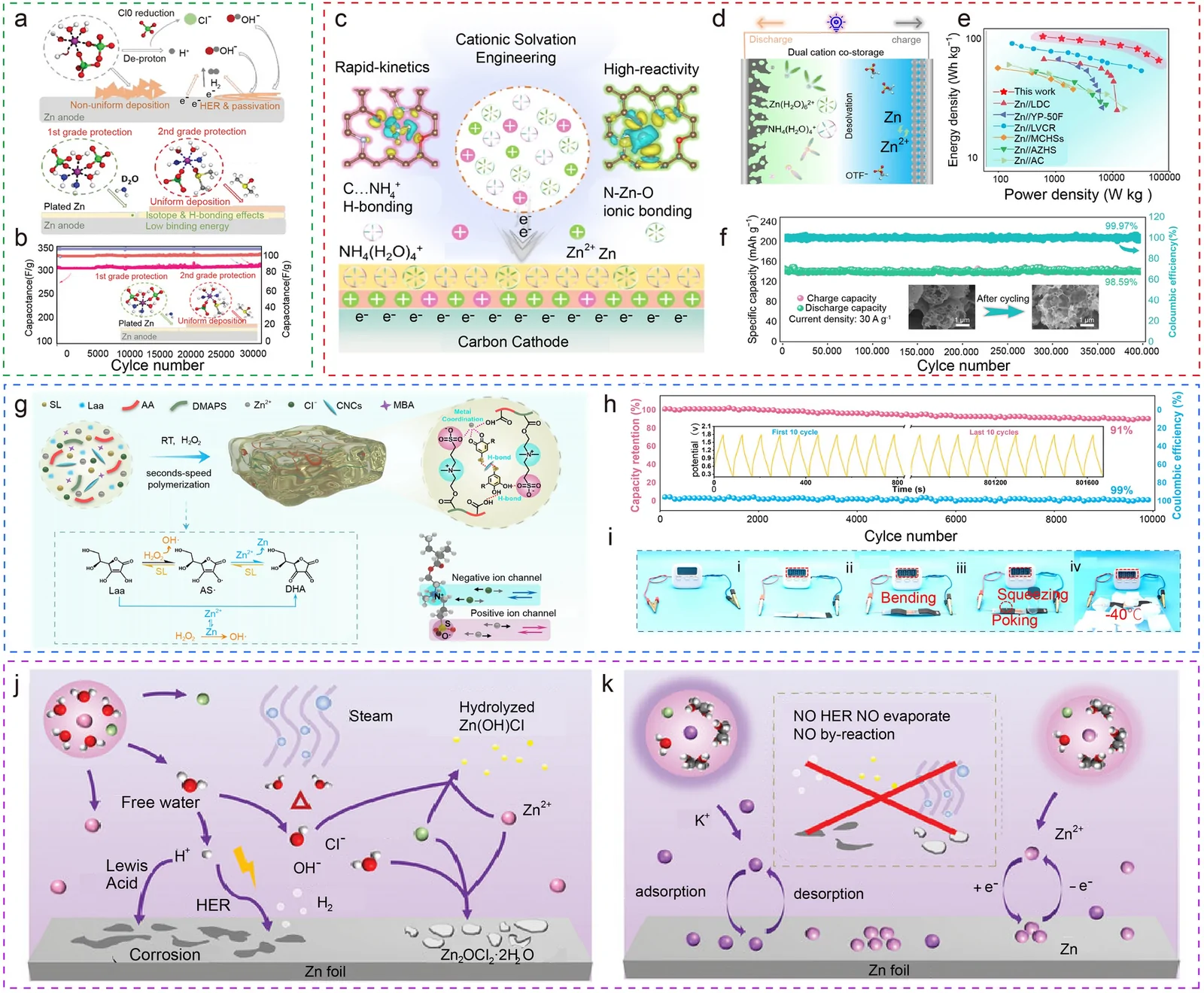
**a** Schematic illustration of Zn^2+^ solvation structure and corresponding interfacial reactions in 5Zn_H (upper) and DIEEs (bottom), respectively. **b** Cycling performance of the scaling-up one-layer pouch cell using the 1Zn_H-D/DMSO electrolyte at 2 A g^−1^ [154]. **c** Schematic diagram of the mechanism for NH_4_⁺-regulated cation solvation to optimize Cathode space charge distribution. **d** Schematic of Zn//PFC capacitors. **e** Ragone plots of Zn//PFC capacitors compared with reported ZICs. **f** Cycling performance of Zn//PFC capacitors [159]. **g** Schematic of the preparation of PAD@SC hydrogel electrolytes. **h** Cyclic stability test of FZICs at 5 A g^−1^ (The insect shows the cycle curves for the initial 10 and the last 10 cycles). **i** Photographs of FZICs powering a timer under different conditions [162]. Schematic illustration of zinc anode reactions showing the different solvation structures and interfacial interaction schemes in **j** 2 M ZnCl_2_ solution and **k** Zn_3_K_4_-HEE/3H_2_O [164]

For instance, a “dual-insurance” eutectic electrolyte (DIEE) was proposed by Lu’s team. By introducing heavy water (D_2_O) and dimethyl sulfoxide (DMSO) into the aqueous electrolyte, the performance of ZICs was significantly improved (Fig. 12a) [154]. Studies have found that the isotopic effect of D_2_O enhances the stability of the hydrogen bond network and reduces the activity of free water molecules, thereby inhibiting side reactions on the surface of the zinc anode. Meanwhile, the addition of DMSO further regulates the solvation structure of Zn^2+^, reduces the activity of free water molecules, and promotes the uniform deposition of Zn^2+^. This dual-solvent protection mechanism not only improves the chemical and electrochemical stability of the electrolyte but also significantly enhances the cycle life and low-temperature performance of ZICs. Experimental results show that the optimized DIEE maintains a capacity retention rate close to 100% over 30,000 cycles and can still work normally at − 20 °C (Fig. 12b). Via molecular dynamics simulations and DFT calculations, the solvation structure of Zn^2+^ and ion transport mechanism in DIEE were revealed, proving contact ion pairs and ion aggregates formation is key to inhibiting side reactions and enhancing performance.

Lee et al. proposed a strategy of adding VOSO_4_ to ZnSO_4_ electrolyte, which utilizes vanadium ions as redox-active species to in situ form a vanadium-zinc hydrate (ZVO) layer rich in oxygen vacancies on the electrode surface [155]. The ZVO layer not only improves the interfacial wettability between the electrolyte and the electrode, reducing the interfacial resistance, but also provides additional active sites for Zn^2+^ intercalation/extraction through oxygen vacancies, significantly enhancing the specific capacity and rate performance of the electrode. Furthermore, during the charge–discharge process, vanadium elements are in situ oxidized and deposited into V_2_O_5_, which further undergoes a reversible intercalation reaction with Zn^2+^ to form a ZVO interface layer with oxygen vacancies, thereby significantly enhancing the pseudocapacitive contribution. After electrochemical activation, the abundant oxygen vacancies in ZVO can serve as fast transport channels for electrons and Zn^2+^, accelerating interfacial charge transfer. Meanwhile, vanadium elements undergo reversible multivalent state transitions (V^5^⁺/V^4^⁺/V^3+^) during cycling, providing additional Faradaic redox reaction sites and expanding the originally limited active area of the carbon electrode. The multi-stage Zn^2+^ intercalation/deintercalation behavior is closely coupled with the stepwise conversion of V^5^⁺ → V^4^ → V^3+^, which significantly improves the specific capacity and reaction kinetics of the electrode. In addition, the ZVO layer acts as a protective layer on the surface of the zinc anode, effectively inhibiting the formation of zinc dendrites, thereby significantly improving the cycle stability of the device (with a capacity retention rate of 91.2% after 10,000 cycles at 10 A g^−1^). Moreover, the specific capacity of the assembled device is increased to 212.6 mAh g⁻^1^ at 0.5 A g^−1^, and the energy density reaches 340.2 Wh kg^−1^. The introduction of such redox-mediated functional additives simultaneously optimizes the ion transport kinetics of the electrolyte and the stability of the electrode/electrolyte interface. This not only provides a crucial insight for addressing the limitations of traditional aqueous electrolytes but also points out a new direction for the high-performance design of ZICs [156]. Although the aforementioned strategies have significantly improved electrolyte stability, interfacial damage and structural mismatch may still accumulate during long-term cycling and in complex mechanical environments, further impairing the electrochemical stability of the electrolyte. Therefore, “dynamically reconstructable” advanced electrolytes, which can achieve adaptive structural repair during operation, have gradually attracted attention. The core highlight of such electrolytes lies in not only inhibiting side reactions or optimizing ion transport, but also proactively maintaining interfacial integrity through the dynamic regulation of physical or chemically reversible bonds, thereby fundamentally enhancing the electrochemical stability of the system.

For example, Chen et al. reported a borax-cross-linked PVA (polyvinyl alcohol)/nanocellulose (NFC) hydrogel electrolyte [157]. Its internal structure consists of a 3D network formed by dynamic cross-linking of reversible borate ester bonds between B(OH)_4_⁻ and –OH groups. This hydrogel electrolyte can rapidly self-heal within approximately 20 s after fracture, with the tensile strength and elongation at break recovering to approximately 98%–99%. This dynamically reconstructable network not only enhances the structural integrity of the electrolyte during deformation or damage but also spontaneously repairs microcracks at the electrode/electrolyte interface during cycling. Consequently, it maintains a continuous transport pathway for Zn^2+^ and stabilizes the interfacial impedance. The quasi-solid-state ZICs constructed based on this electrolyte can still recover most of their capacity after cutting-healing, demonstrating excellent interfacial repair capability and cycling reliability.

Recent studies have also achieved structurally adaptive regulation of electrolytes by constructing multi-level ion-polymer interactions. For instance, the synergistic effect between polyoxometalates (POMs) and ionic liquids (ILs) in a polyampholyte network can prevent macroscopic phase separation in traditional POM-IL systems and form a worm-like microphase-separated structure, thereby constructing continuous ion channels [158]. This network is maintained by electrostatic interactions, hydrogen bonds, and segmental rearrangement, endowing the hydrogel with high mechanical strength and antifreeze properties, while also exhibiting self-healing and dynamic reconstruction capabilities. After interfacial damage occurs, the hydrogel can restore structural integrity through the migration of local segments. ZICs constructed based on this ionic hydrogel achieve a specific capacitance of 160 F g^−1^ at a current density of 0.1 A g^−1^, and retain approximately 36.4% of their capacity even after 2 ,500 cycles in an open environment. These results further demonstrate the advantages of structurally adaptive electrolytes in enhancing long-term cycling stability.

In summary, dynamically reconstructable or self-healing electrolytes can be regarded as another effective approach to address the insufficient electrochemical stability of aqueous electrolytes. By adaptively regulating the structure and repairing interfacial microdamage during operation, they fundamentally enhance the safety, durability, and environmental stability of both ZC-ZICs and CB-ZICs, providing a new design concept for constructing high-stability aqueous energy storage systems.

### Kinetic Retardation

In the ZICs system, although traditional Zn^2+^ carriers possess high intrinsic reaction activity, their strong hydration properties lead to the formation of a bulky solvated structure ([Zn(H_2_O)_6_]^2+^) with a high desolvation energy barrier of up to 14.90 eV. This significantly increases ion migration resistance and slows down the interfacial reaction kinetics. Specifically, at high-current densities, Zn^2+^ struggles to rapidly desolvate and intercalate/extract at the electrode interface, causing concentration polarization and uneven zinc deposition. Eventually, this accelerates dendrite growth and deteriorates cycle stability. This kinetic bottleneck has become a core challenge restricting the improvement of power density in zinc-based energy storage devices, and it is urgent to achieve a breakthrough through the reconstruction of electrolyte solvation structures or the regulation of interfacial energy barriers.

As shown in Fig. 12c, Chen’s team constructed a hierarchical solvated structure by codissolving NH_4_^+^ (with a small hydrated size and a low desolvation energy of 5.81 eV) and Zn^2+^ in a Zn(CF_3_SO_3_)_2_-NH_4_CF_3_SO_3_ mixed electrolyte [159]. This successfully reconstructed the Helmholtz plane at the cathode interface, optimized the space charge distribution, and increased the capacitance density by 20%. The mixed electrolyte exhibited higher ionic conductivity and wettability, and enhanced adsorption of positive charges at the interface was verified by zeta potential tests. In addition, the flexible tetrahedral structure of NH_4_^+^ and its hydrogen-bonding interactions provided more adsorption sites, enhancing charge storage capacity, while Zn^2+^ formed stable N–Zn–O bonds through strong coupling, further improving charge storage density. Experiments showed that the synergistic effect of Zn^2+^ and NH_4_^+^ not only improved capacity through physical adsorption and redox reactions but also provided the device with excellent rate performance (130 mAh g^−1^@50 A g^−1^) and ultra-long-cycle life (98.59% capacity retention after 400,000 cycles) due to the low desolvation energy of NH_4_^+^ (Fig. 12d–f). Although the introduction of NH_4_⁺ significantly enhanced Zn^2+^ kinetics, there is still room for improvement in electrolyte viscosity and ion migration efficiency. The strategy by Chen’s team provides an important reference for optimizing Zn^2+^ kinetics. However, to further break through the kinetic bottleneck, researchers have proposed an innovative solution based on quasi-eutectic electrolytes (quasi-EE) [160]. Researchers have innovatively addressed the core issue of sluggish electrolyte kinetics in ZICs by designing a novel quasi-EE system. An “intrinsic decoupling” strategy is proposed, which constructs a quasi-eutectic structure with both a deep eutectic stable framework and low-concentration electrolyte (LCE) microdomains by regulating the molar ratio of anhydrous zinc acetate (Zn(Ac)_2_) to formamide (Fad). In this structure, free molecules not involved in the eutectic network form dispersed LCE microdomains, significantly reducing the system viscosity and accelerating Zn^2+^ migration through a mixed transport mechanism. Molecular dynamics simulations further reveal that the Zn^2+^ solvation shell in quasi-EE is composed of [Zn(Fad)_2_(Ac-)_2_]. The gradient distribution of coordination strength reduces the size of ion clusters, improves spatial dispersion, exposes free Zn^2+^, and lowers the desolvation energy barrier. In addition, LCE microdomains achieve dense and uniform zinc deposition by enhancing the adsorption capacity of the electrode interface and optimizing nucleation kinetics, thereby inhibiting dendrite growth. This strategy, which synergistically optimizes through local structure decoupling and transport mechanism regulation, provides a new paradigm for breaking through the electrolyte kinetic bottleneck in high-energy storage devices.

### Narrow Voltage Window

The practical application of ZICs is limited by their narrow voltage window (usually < 2.0 V), and the critical flaws in electrolyte systems are the core bottleneck restricting this performance. In traditional aqueous electrolytes, the inherently low decomposition voltage of water molecules easily triggers HER and oxygen evolution reaction (OER), leading to instability at the electrode/electrolyte interface and frequent side reactions, which severely compress the effective operating voltage of the device. How to break through the “voltage ceiling” of water decomposition through electrolyte engineering while synergistically optimizing interface stability has become a key challenge in promoting ZICs to enter the field of high-energy density energy storage.

Recently, Chen’s team proposed a weak solvating zinc-electrolyte system based on a mixed solvent of acetonitrile (AN) and tetramethylene sulfone (TMS), achieving a wide-voltage window for ZICs [161]. Molecular dynamics simulations show that the coordination distance between Zn^2+^ and AN/TMS molecules is extended to 3.3–3.8 Å, which significantly reduces the solvation energy barrier, thereby achieving high reversibility of zinc deposition/stripping. Combined with FT-IR spectroscopy and DFT calculations, it is found that the strong adsorption of oxygen atoms in TMS on the zinc electrode surface promotes the preferential growth of the (001) crystal plane and inhibits dendrite formation. ZICs devices based on this electrolyte achieve a wide-voltage window of 2.0–2.5 V, with an energy density of 135 Wh kg^−1^ (at 0.5 A g^−1^). Even at an ultra-high-current density of 15 A g^−1^, they still maintain an energy density of 29.3 Wh kg^−1^ and a capacity retention rate of 81.2% after 9000 cycles. This weak solvation electrolyte design provides new ideas for solving key challenges in zinc-based energy storage systems. Traditional methods relying on organic solvents or additives can broaden the ESW but often lead to reduced ionic conductivity or safety hazards.

In addition, improving the device's voltage window through electrolyte gelation strategies is also a commonly used method. For example, Liu et al. reported a polyampholyte hydrogel electrolyte (PAD@SC) prepared by an ultra-rapid autocatalytic gelation strategy [162]. The study constructed an autocatalytic redox system using L-ascorbic acid, ZnCl_2_, and H_2_O_2_, which rapidly initiated the copolymerization of acrylic acid and sulfobetaine monomer within 50 s at room temperature. Combined with the reconstruction of hydrogen bond networks using bio-based components sodium lignosulfonate and cellulose nanocrystals, the mechanical strength and interfacial adhesion of the hydrogel were significantly enhanced (Fig. 12g). The zwitterionic structure forms independent high-speed ion channels, endowing the electrolyte with a high Zn^2+^ transference number and ionic conductivity, and inhibiting dendrite growth by regulating the Zn^2+^ solvation structure. In addition, density functional theory calculations show that the strong binding energy between polymer chains and Zn^2+^ preferentially captures Zn^2+^, reduces water molecule contact, and broadens the ESW to 2.4 V. Flexible ZICs assembled based on PAD@SC exhibit high specific capacity (85.4 mAh g^−1^@1 A g^−1^), excellent rate performance (44.1 mAh g^−1^ at 10 A g^−1^), and ultra-long-cycle life (91% capacity retention after 10,000 cycles), while also possessing antidehydration (only 5% weight loss in 120 h) and low-temperature resistance (−40 °C) properties (Fig. 12h, i). Zeng et al. reported a design strategy based on sulfonic acid-based zwitterion (SBMA) hydrogel electrolytes [163]. Studies have found that the unique double-charge structure of zwitterions can strongly bind water molecules through electrostatically induced hydration, and trigger the stretch of chain structures in salt solutions due to the ion shielding effect, thereby inhibiting hydrogen/oxygen evolution reactions. This strategy significantly expands the ESW to 2.58 V. The SBMA hydrogel electrolyte designed based on this strategy possesses both high-ionic conductivity and a wide ESW. The assembled ZICs achieve an operating voltage of 2.1 V (compared to 1.6 V in traditional systems), a capacity of 188.9 mAh g^−1^, and an energy density of 110 Wh kg^−1^, which is significantly better than that of the CBMA system (82.1 Wh kg^−1^). This research provides a new paradigm for designing high-performance electrolytes by regulating the zwitterion APE effect, promoting the development of high-energy density energy storage devices.

### Poor Low-Temperature Performance

The temperature sensitivity of traditional aqueous electrolyte systems severely restricts the practical application of ZICs: At low temperatures, the electrolyte is prone to freezing, leading to blocked ion transport; at high temperatures, intensified side reactions trigger zinc dendrite growth and interface failure, resulting in a sharp decline in device performance in extreme environments. The core to breaking this “temperature adaptability shackle” lies in the design and modification of electrolytes—by regulating solvent–solute interactions, constructing stable interfaces, and inhibiting thermally induced side reactions, the operating temperature range of devices can be significantly broadened.

In recent years, innovative strategies based on deep eutectic solvents and functionalized gel electrolytes have provided new ideas for the temperature universality of ZICs. Nan's team designed a hydrogel electrolyte with both high adhesion and antifreeze performance [165]. By regulating the concentration of ZnCl_2_ and LiCl mixed salts to 5:12, the study utilized a synergistic solvation effect to break the hydrogen bond network of water molecules, lowering the freezing point of the hydrogel to below −80 °C while maintaining ionic conductivity at −60 °C. Molecular dynamics simulations showed that the introduction of Li⁺ reduced the agglomeration of Zn^2+^ and Cl^−^_,_ optimizing ion transport pathways. Zinc/carbon nanotube hybrid capacitors based on this electrolyte retained an energy density of 39 Wh kg^−1^ and a capacity retention rate of 98.7% after 10,000 cycles at −60 °C, and could withstand dynamic deformation. Their performance was significantly superior to traditional low-temperature aqueous energy storage devices. Wen designed a unique bimetallic solvation structure using EG and bimetallic salts (ZnCl_2_/KCl). Zn^2+^ and K^+^ formed solvation shells of [Zn(EG)_3_H_2_OCl]⁺ and [K(EG)_3_H_2_OCl], respectively, reducing the stepwise desolvation energy to 1.78 eV (Zn^2+^) and 1.57 eV (K^+^), thereby promoting rapid ion migration [164]. Experiments showed that this electrolyte maintained high-ionic conductivity even at 100 °C, enabling ZICs to achieve a wide ESW of 2.1 V, an energy density of 343.2 Wh kg^−1^, along with a capacity retention rate of 97.5% following 40,000 cycles. Its excellent performance stems from the fact that EG forms a hydrogen bond network with water to bind free water, inhibiting water decomposition and zinc corrosion. Moreover, the introduction of K⁺ optimizes the solvation structure, reduces the agglomeration of Zn^2+^ and Cl⁻, and enhances reaction kinetics. In addition, the stable hydrogen bond network at high temperatures maintains the fluidity of the electrolyte, ensuring applicability over a wide temperature range. This research provides new ideas for the design of electrolytes for wide-voltage and high-temperature-resistant ZICs (Fig. 12j, k). Surprisingly, Wan et al. designed a gel polymer electrolyte (GPE) with high adhesiveness, thermal stability, and antifreeze properties based on a polyanion-induced Zn^2+^ interpenetrating network structure, which is used for ZICs with a wide temperature range and interface stability [166]. This GPE utilizes the sulfonic acid groups of 2-acrylamido-2-methylpropanesulfonic acid as active sites to form stable metal–ligand coordination with Zn^2+^, which improves the mechanical strength and interfacial adhesion of the electrolyte while inhibiting the growth of zinc dendrites. The introduced EG and high-concentration ZnCl_2_ further enhance the ionic conductivity and wide temperature range adaptability of the electrolyte. The assembled ZICs exhibit excellent electrochemical performance, cycle stability, and interfacial compatibility over a wide temperature range.

In addition to modifying electrolytes using deep eutectic solvent strategies to broaden the operating temperature range of ZICs, many researchers have adopted other modification approaches. For instance, Gao’s research team achieved excellent mechanical flexibility and antifreeze performance by constructing a double-network polymer structure, which can maintain high-ionic conductivity over a wide temperature range from −30 to 60 °C [167]. Peng et al. proposed a hydrogen bond-anchored electrolyte design strategy, which reconstructs the hydrogen bond network of water molecules by introducing sulfolane, thereby significantly improving the stability and temperature adaptability of ZICs [168]. This hydrogen bond-anchored electrolyte not only enhances the reversibility and stability of the zinc anode but also enables ZICs to exhibit excellent cycle stability and capacity retention at extreme temperatures (−20 to 60 °C).

### Security Risks

Compared with systems such as lithium-ion batteries that rely on flammable organic electrolytes, ZICs, with water-based or solid electrolytes as their core, fundamentally solve safety hazards such as combustion, liquid leakage, and dendrite short circuits. Through the regulation of electrolyte composition, interface optimization, and innovation in physical form, this technology not only achieves “intrinsic safety” but also shows significant advantages in cycle life and environmental adaptability, providing reliable energy storage solutions for fields such as flexible electronics and grid energy storage.

Recently, hydrogel electrolytes have attracted increasing attention. Cui et al. designed a multi-functional polyampholyte hydrogel electrolyte to address the safety hazards in ZICs. The polar functional groups in the hydrogel (–COO^−^, –SO_3_^−^, –C=O, and -NHCO-) significantly reduce the activity of water by forming a large number of hydrogen bonds with water molecules, thereby inhibiting side reactions and ice crystal formation, while enhancing the safety of the device under extreme temperatures [169]. In addition, the coordination of polar functional groups with Zn^2+^ homogenizes the electric field distribution, effectively inhibiting the growth of zinc dendrites and reducing the risk of device short circuits. The low water content and high mechanical strength of the hydrogel prevent the leakage of liquid electrolytes, while its flexibility and self-adhesiveness ensure close contact between the electrode and the electrolyte, further enhancing the mechanical stability and environmental adaptability of the device. Through these mechanisms, the hydrogel electrolyte boosts the safety, cycle life, and operating temperature range of zinc-based energy storage devices.

Furthermore, Liu et al. designed a hydrogel electrolyte with a betaine (BT) additive. BT molecules are preferentially adsorbed on the zinc surface to form a hydrophobic EDL, and construct a gradient organic/inorganic electrolyte interface through synergistic interaction with Zn(BF_4_)_2_ [170]. This structure effectively isolates water molecules, reduces water-related side reactions, and promotes the uniform deposition of Zn^2+^. The adsorption of BT molecules and the derived organic interface lower the desolvation energy of Zn^2+^, accelerate the diffusion and deposition kinetics of Zn^2+^, thereby inhibiting dendrite growth and preventing dendrite-induced short circuits in ZICs. This strategy provides an effective approach for optimizing the zinc anode interface and improving the safety and reliability of zinc-based energy storage systems.

Hydrogel electrolytes enhance safety by inhibiting the growth of zinc dendrites and other means. However, hydrogel electrolytes still have some limitations. To further improve safety, researchers have begun to explore the use of solid electrolytes to replace hydrogel electrolytes [171]. One study addressed safety hazards in traditional zinc-ion energy storage devices by designing an all-solid-state fibrous ZICs based on a “scabbard” structure. By using gelatin/ZnSO_4_ as the solid electrolyte, the device avoids the risk of liquid electrolyte leakage. Meanwhile, it optimizes the contact area between the electrolyte and the electrode, reducing electrolyte exposure and the occurrence of side reactions. In addition, the “scabbard”-like structure remarkably shortens the ion transport pathway and boosts the contact area between the electrode and the electrolyte, thereby improving ion diffusion efficiency and electrochemical stability. This design not only enhances the mechanical flexibility and cycle stability of the device but also reduces safety hazards by minimizing water-related side reactions. Ultimately, the device maintains stable electrochemical performance under bending conditions, demonstrating excellent mechanical stability and safety, and providing a reliable energy storage solution for flexible wearable electronic devices.

## Conclusion and Prospect

### Conclusion

ZICs have emerged as a highly promising candidate for next-generation energy storage, effectively bridging the gap between batteries and supercapacitors by offering a unique combination of high-energy density, high-power density, and exceptional cycle life. Their advantages, including intrinsic safety, low-cost, and environmental friendliness, further bolster their potential for large-scale applications. This review systematically categorizes ZICs into two primary configurations based on their working principles: ZC-ZICs and CB-ZICs. For each configuration, the energy storage mechanisms are thoroughly analyzed, and the key challenges are identified alongside corresponding strategic solutions. A significant focus is placed on the critical role of electrode materials. For capacitive cathodes, particularly carbon-based materials, strategies to combat limitations like insufficient SSA, limited charge storage mechanisms, and slow ion diffusion kinetics are discussed, including heteroatom doping, pore structure engineering, and composite material design. The challenges and modifications for battery-type anodes and cathodes (e.g., vanadium-based and manganese-based compounds) are also elaborated upon, highlighting approaches to inhibit dendrite growth, side reactions, and structural degradation. Furthermore, the pivotal role of the electrolyte is emphasized, as its properties directly dictate the device’s voltage window, kinetics, and low-temperature performance. The review summarizes progress in optimizing electrolytes through pH regulation, functional additives, and the development of novel systems like hydrogels and eutectic electrolytes to address issues such as hydrogen evolution, zinc dendrites, and freezing. Ultimately, this work provides valuable insights and guidance for the rational design of high-performance ZICs, paving the way for their future integration into advanced energy storage systems.

### Prospect

**Fig. 13:**
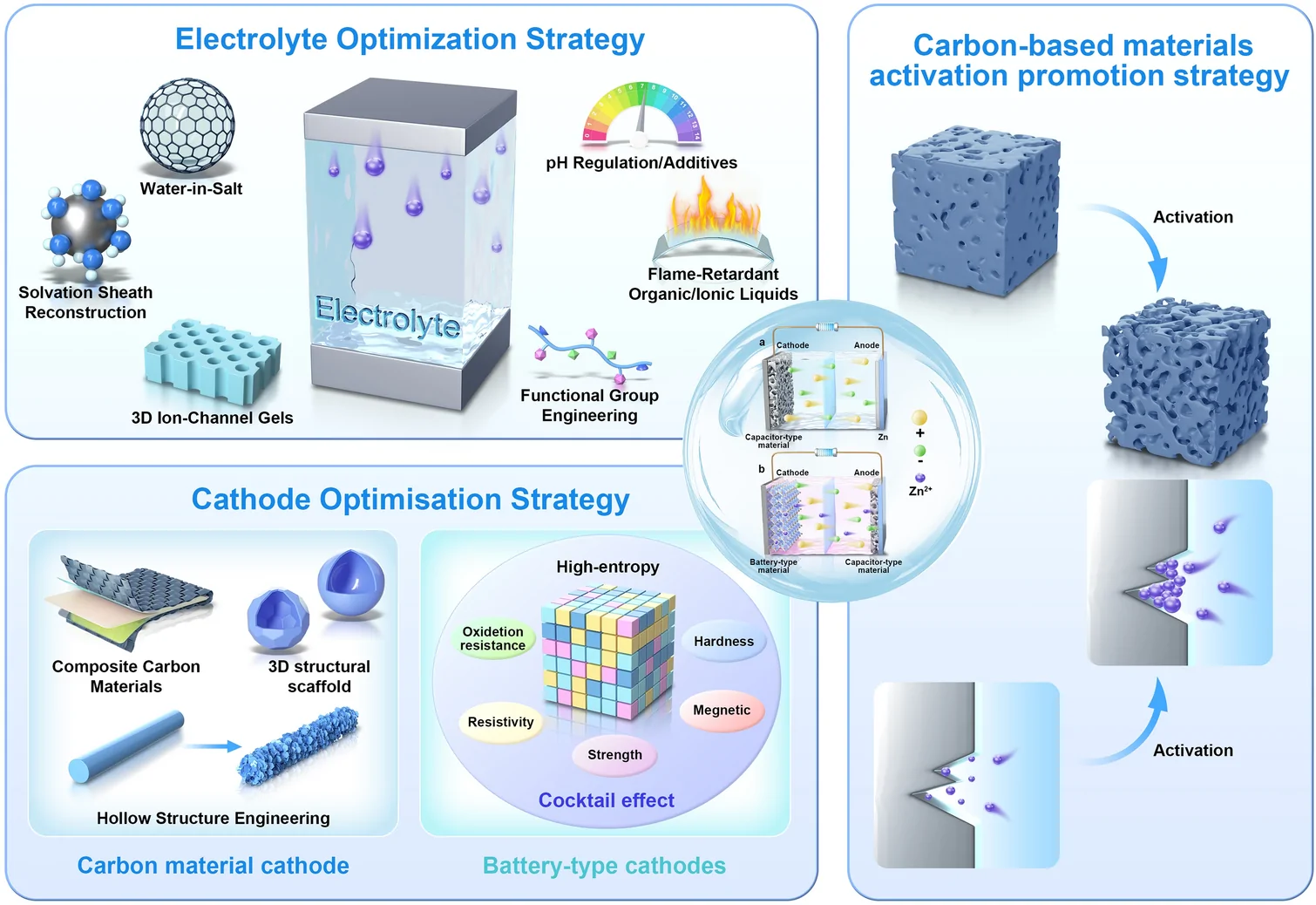
The future developing directions of ZICs

While the above content summarizes numerous significant advances, it is evident that the current development of ZICs still fails to meet the requirements for practical large-scale energy storage. Issues such as capacity mismatch between the cathode and zinc anode, insufficient reversibility of zinc plating/stripping, and the inherent limitations of existing electrolyte systems collectively restrict further improvements in energy density, power performance, and long-term reliability. These bottlenecks indicate that optimizing individual components alone is no longer sufficient to advance the technology; instead, there is an urgent need to achieve system-level breakthroughs that integrate electrode activation, structural reconstruction, and electrolyte innovation. Based on these insights, the following outlook section will discuss several highly promising research directions. These directions directly address the aforementioned fundamental issues and may provide guidance for the next major leap in ZICs technology (Fig. 13).

#### Activation Promotion Strategy of Carbon-Based Materials

Increasing the specific capacity of carbon-based materials is one of the core approaches to enhancing the energy density of ZICs, and it is also a key step in addressing the widely existing N/P imbalance issue in current ZICs. Recent studies have shown that electrochemical “activation” strategies, especially pulse voltage or high-current long-cycle methods, can reshape the microstructure of carbon materials and effectively improve their energy storage capability. For instance, some studies have reported that the application of pulse voltage can significantly recover or enhance the capacity of carbon electrodes [172]. Therefore, based on the high plasticity of carbon electrode materials, pulse voltage activation is regarded as a powerful technical pathway to improve the capacity of carbon-based materials and thereby promote the enhancement of ZICs’ energy density.

Pulse voltage helps to improve the capacity of carbon-based materials. The performance of carbon-based materials is often limited by their pore structure and SSA, preventing them from achieving high specific capacity and excellent energy density. Previous studies have reported that some voltage or long-cycle strategies can improve the structure of materials, and then construct special ion transport channels to optimize the performance of energy storage devices. For instance, Cui et al. reconnect the isolated active material in the electrode material back to the conductive network by using a pulse voltage, achieving a capacity recovery of 30% [172]. In addition, some energy storage devices will have a capacity improvement mechanism after a long cycle of high current. The corresponding reason may be that some structures collapse during the cycle and expose more active surfaces. Based on this, this study believes that pulse voltage and long cycle are very promising strategies to enhance the capacitance of carbon-based materials. The use of pulse voltage or long cycle is expected to ameliorate the structure of carbon materials. On the one hand, it can force the pores in the carbon material that are not conducive to transmission to gradually evolve into pores that are conducive to ion transmission during the long-term transmission of high voltage and ions. On the other hand, high voltage and long cycle can stimulate the structure of carbon materials, drive its structural reorganization, reveal a broader active SSA, and achieve high performance of ZICs. For example, Fan et al. constructed a high-performance Zn//activated carbon micro-ZIC on planar gold electrodes using microfabrication technology. This device maintains stable energy output and rapid charge response even under extremely high-current densities, demonstrating the great potential of carbon-based electrodes in the directions of high-power density and miniaturization. Such studies indicate that through the synergistic effect of electrochemical “activation” and microscale structural optimization, carbon-based materials can achieve fast and reversible ion migration under high pulse currents, providing new design insights for electric vehicle power modules and rapid-response energy storage systems [173]. This is particularly crucial for ZICs that need to withstand high-current pulses (> 10 A g^−1^), which can be applied in rapid-response scenarios such as grid buffering or electric vehicles. Looking ahead, rationally designed electrochemical “activation” protocols are expected to serve as pre-conditioning strategies, endowing carbon electrodes with the ability to maintain structural stability and rapid kinetic response—thereby enabling high-performance zinc-ion capacitors in extreme environments.

#### Future Cathode Development Directions for ZICs

The rational design of cathode materials will remain a core lever for breaking through the performance limits of ZICs. Besides the relatively low capacity of traditional carbon-based cathodes, which is a major bottleneck restricting energy density, the selection of cathode type fundamentally determines the working mechanism, operating voltage window, and rate characteristics of ZICs. Therefore, the future development of cathode materials must advance along differentiated yet synergistic paths, and needs to be adapted to specific ZIC configurations.

For ZC-ZICs, which rely on capacitive cathodes, the future lies in moving beyond traditional porous carbons through precise pore architecture engineering at the molecular level to optimize ion transport, advanced multi-heteroatom doping to maximize pseudocapacitive contributions, and integration with highly conductive 2D materials like MXenes to form efficient 3D networks. Concurrently, the exploration of novel “smart” carbon forms such as 3D-printed ordered scaffolds and graphdiyne will be key to achieving high-power density and mechanical flexibility. For CB-ZICs that utilize battery-type cathodes, the research frontier mainly focuses on the development of high-entropy electrode materials. High-entropy materials are typically defined as a class of multi-component materials—generally containing 5–10 principal metal elements in equimolar or near-equimolar ratios—and form a single-phase crystalline structure driven by high configurational entropy [174]. By virtue of this characteristic, such materials can exhibit unique synergistic effects. Leveraging advanced synthesis techniques like ultrafast thermal shock and electrochemical deposition, the rational incorporation of multiple metal elements into a single-phase lattice enables the generation of a synergistic “cocktail effect”. This strategy aims to enhance the device performance from three aspects: improving the structural stability of the material (suppressing phase transitions during cycling through entropy increase), boosting intrinsic conductivity (optimizing the electronic band structure via lattice distortion), and endowing the material with richer redox properties (utilizing the diverse valence state changes of multiple metal cations)—thereby significantly optimizing the capacity and cycle life of the device.

#### Development Directions for ZIC Electrolytes

The electrolyte is at the core of nearly all key challenges that constrain the practical performance of ZICs—including zinc dendrite growth, parasitic side reactions, voltage limitations, and poor low-temperature adaptability. Unlike electrodes, whose performance is largely determined by the intrinsic properties of materials, the electrolyte governs the entire electrochemical environment. It directly influences the solvation structure of Zn^2+^, interfacial chemical properties, and ion transport kinetics, and ultimately affects the reversibility of zinc plating/stripping. Therefore, advancing electrolyte design is a decisive pathway to unlocking the full potential of ZICs. Future electrolyte systems need to evolve from single-function formulations to multi-functional, environment-adaptive chemical systems, capable of stable operation across a wide temperature range, high-current densities, and diverse application scenarios.

For aqueous electrolytes, the primary goal is to break the theoretical voltage window constraint. Strategies like formulating highly concentrated “water-in-salt” electrolytes, introducing pH-regulating additives, and employing functional molecules to reconstruct the solvation sheath of Zn^2+^ ions will be crucial to suppress HER and widen the ESW, thereby boosting energy density. In high-temperature environments (> 60 °C), the aforementioned strategies must also be combined with corrosion inhibitors and thermally stable coordination structures to suppress accelerated side reactions, reduce electrolyte volatilization, and mitigate Zn corrosion—thus enabling applicability in scenarios such as photovoltaic grids or desert microgrids. Under low-temperature conditions (< − 20 °C), it is necessary to develop aqueous or hybrid electrolytes with reduced freezing points, weakened hydrogen bond networks, and tunable Zn^2+^ solvation structures. These modifications aim to alleviate issues of increased viscosity and sluggish ion transport, allowing ZICs to be competent for applications like cold-region energy storage or aerospace electronics [175]. Addressing the safety concerns of non-aqueous electrolytes (organic and ionic liquids) without sacrificing their high-voltage advantage is another key direction. This will involve developing novel, non-flammable organic solvents or ionic liquids with higher thermal stability and lower toxicity to mitigate safety risks. The most promising path forward lies in the advanced design of gel polymer electrolytes and hydrogels. Future research must solve their inherent issues of poor ionic conductivity and limited electrode contact by constructing 3D hierarchical ion transport channels, integrating nanofillers for enhanced mechanics and conductivity, and engineering functional groups (e.g., zwitterions, sulfonic groups) that guide uniform zinc deposition and improve interfacial compatibility.

Ultimately, the ideal electrolyte will be a hybrid or quasi-solid system that merges the high safety of aqueous systems, the high voltage of non-aqueous systems, and the flexibility and dendrite-inhibiting properties of advanced gels, enabling high-performance, durable, and safe ZICs for a wide range of applications. Furthermore, the electrolyte plays a critical role in the construction of novel hybrid systems, such as integrating ZICs with zinc-air batteries to create self-charging units. For such systems, the development of a stable, multi-functional aqueous electrolyte that can support both the reversible plating/stripping of zinc and the oxygen reduction/evolution reactions is a key frontier. Ultimately, the ideal electrolyte should be a hybrid or quasi-solid-state system that combines the high safety of aqueous systems, the high-voltage advantage of non-aqueous systems, as well as the flexibility, dendrite-inhibiting, and leakage-proof properties of advanced gels. Meanwhile, it should maintain sufficient ionic conductivity and interfacial stability under operating conditions of high-temperature, low-temperature, and ultra-high-current density. With the help of such electrolyte systems, ZICs are expected to achieve high performance, long lifespan, and high safety across a variety of application scenarios—from flexible wearable devices and miniaturized self-powered electronic devices to grid-scale energy storage in extreme environments.
